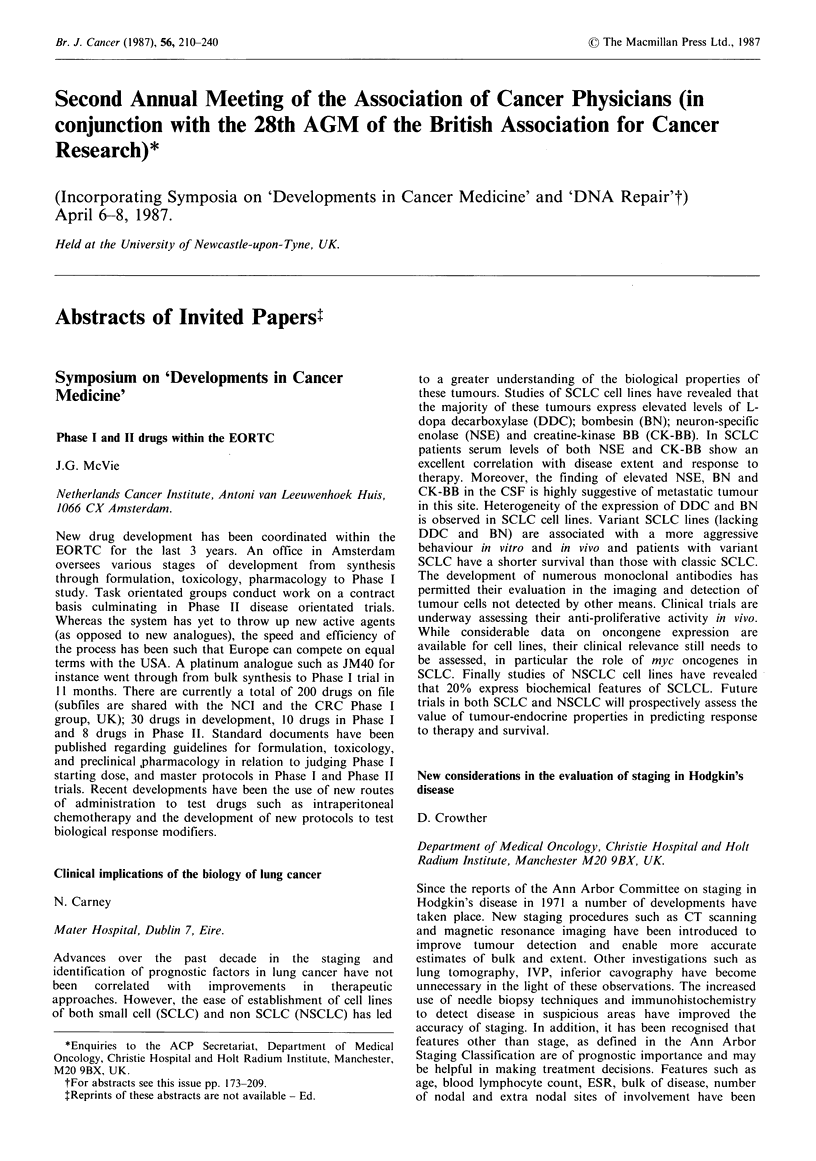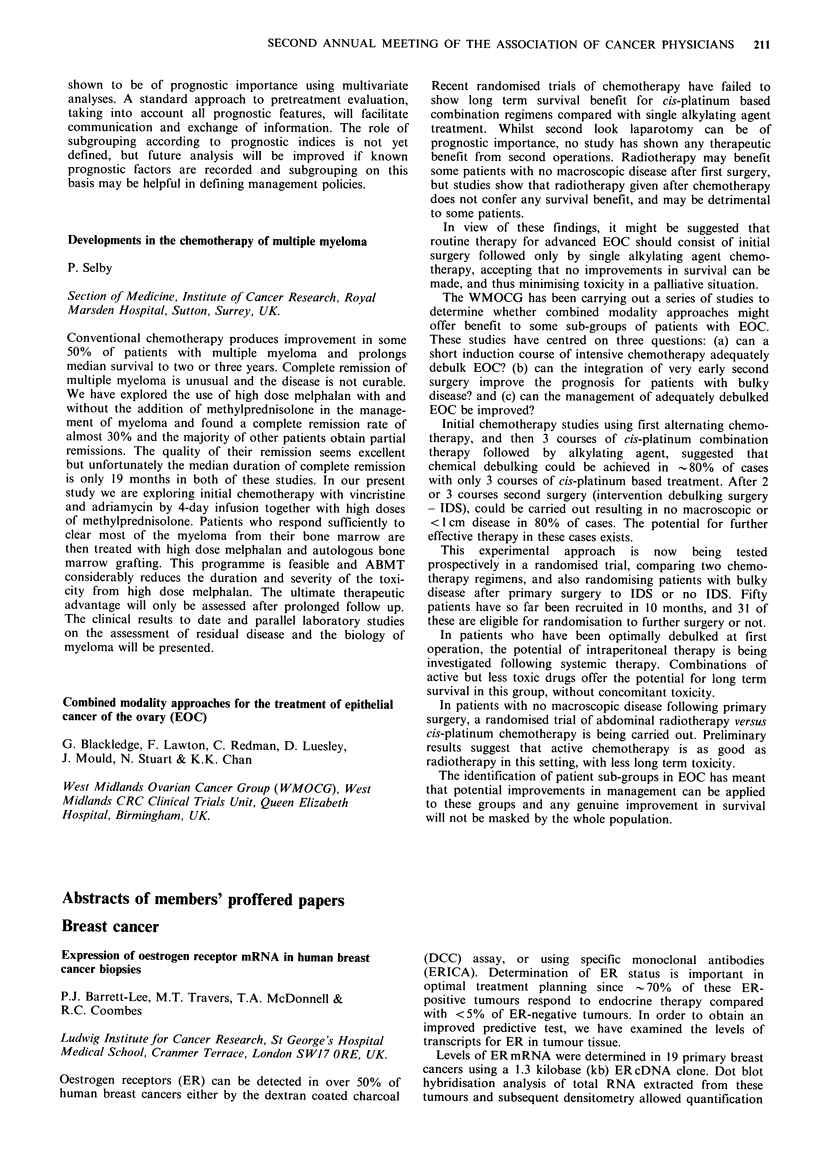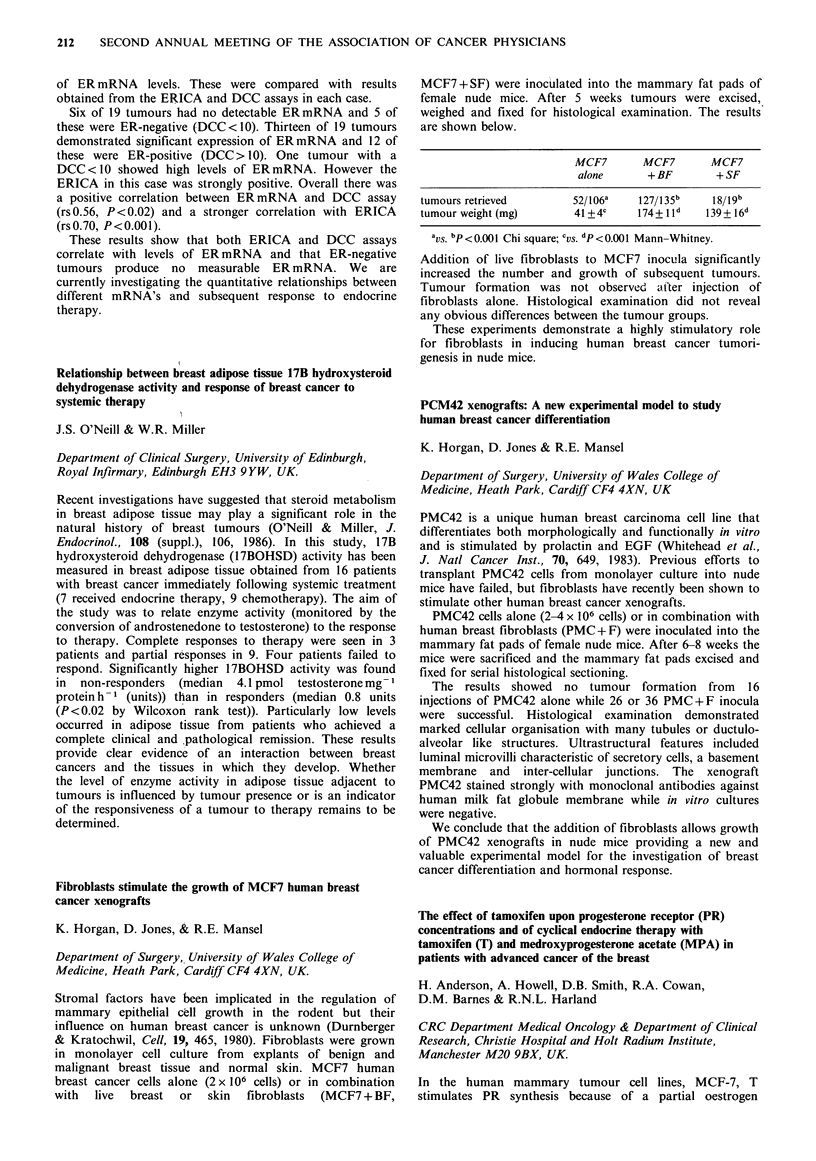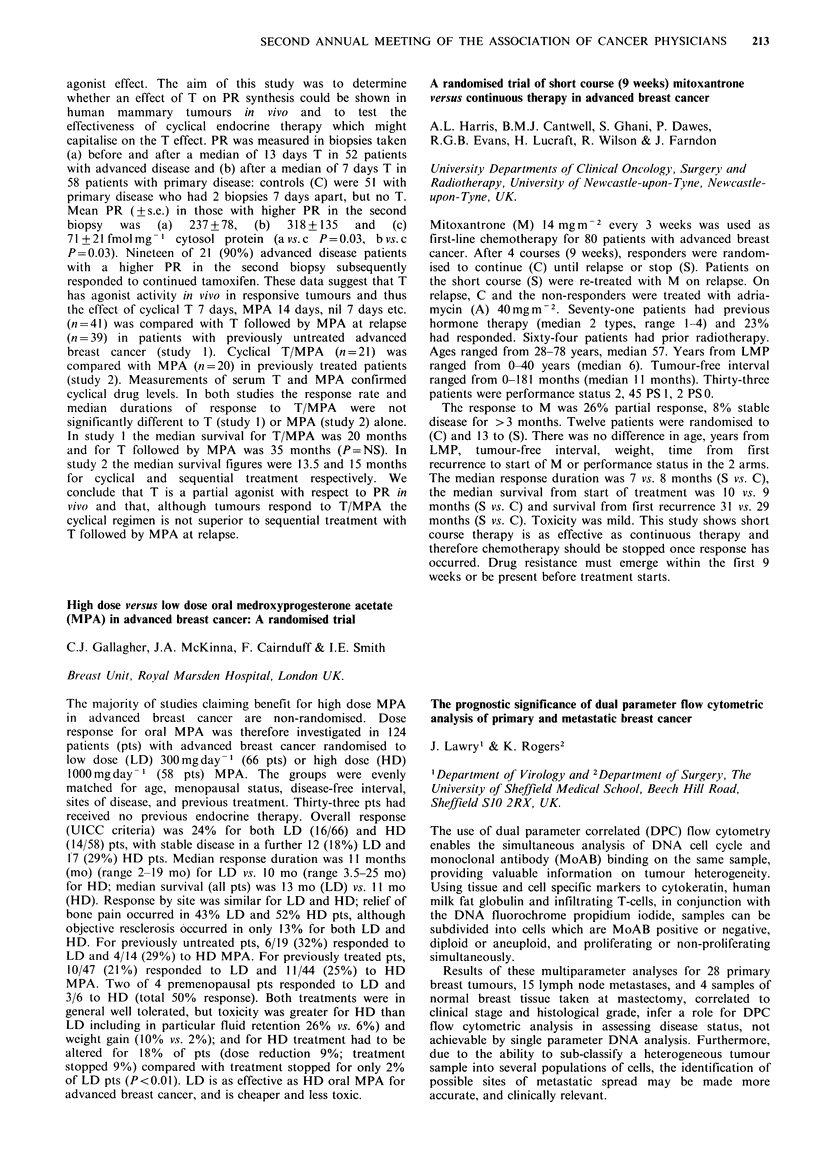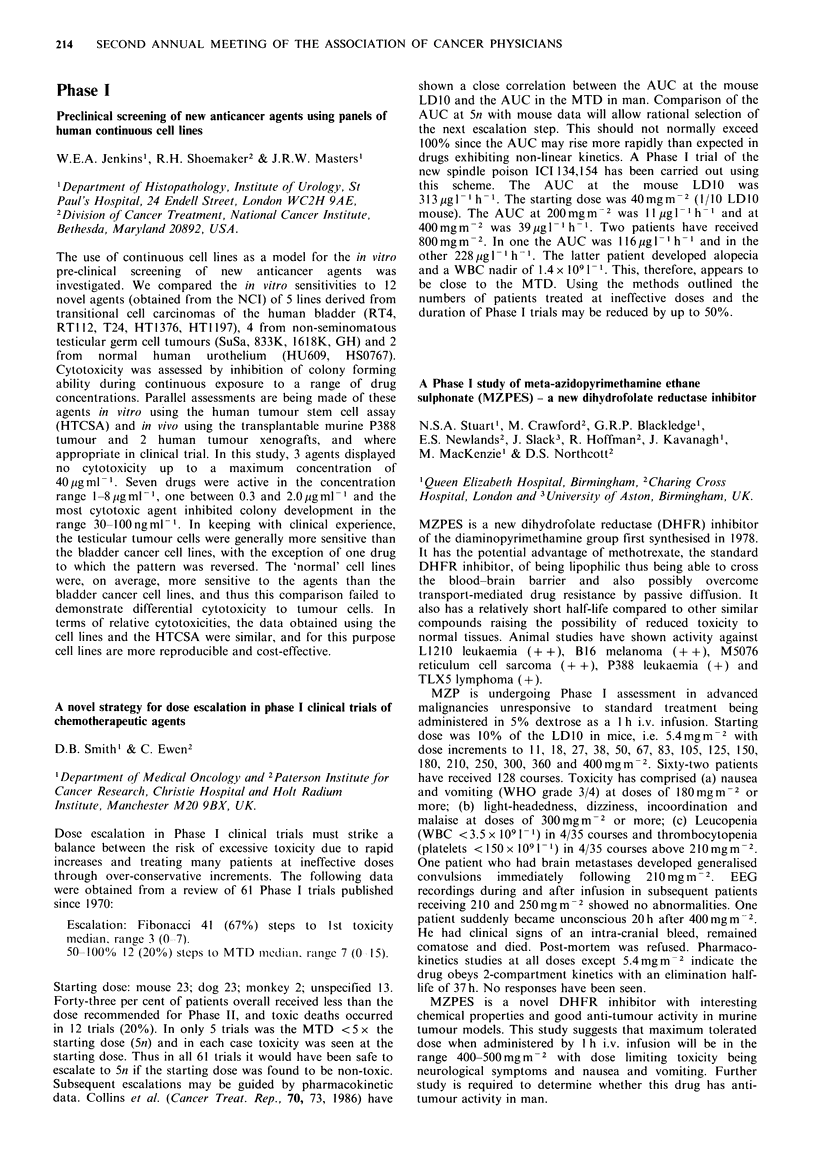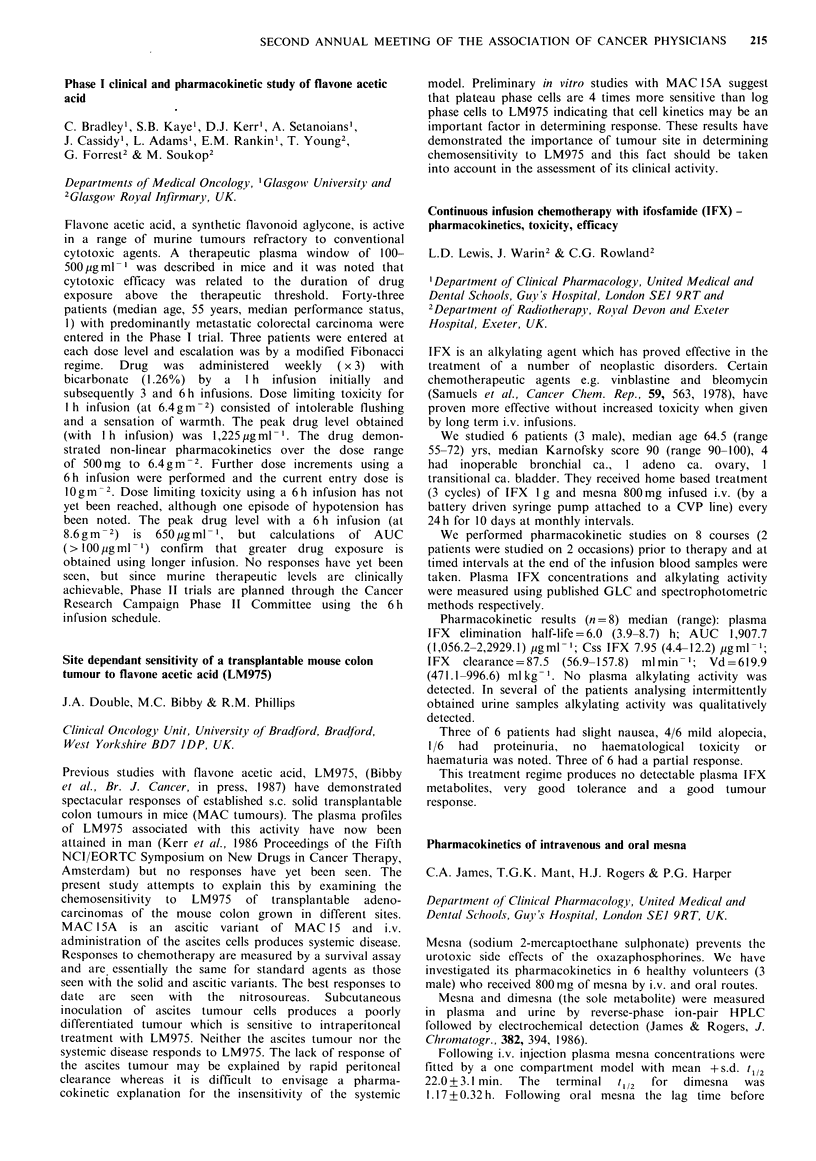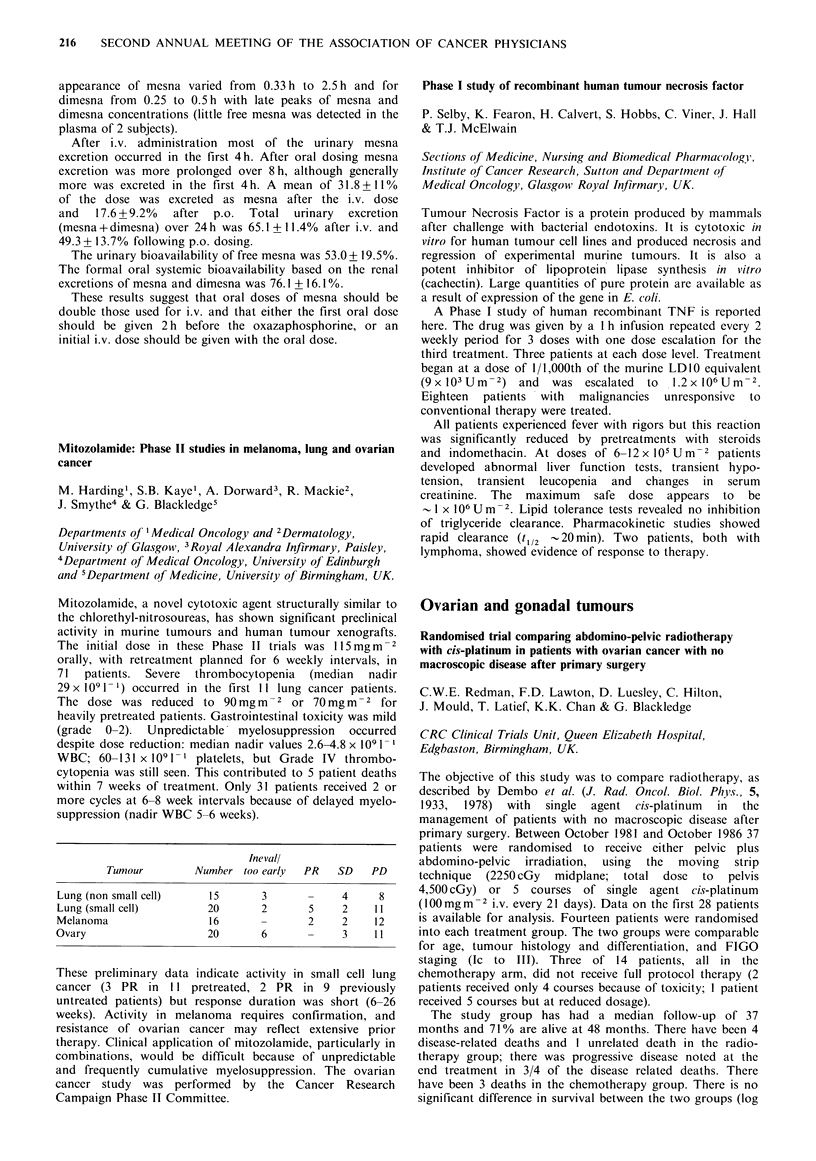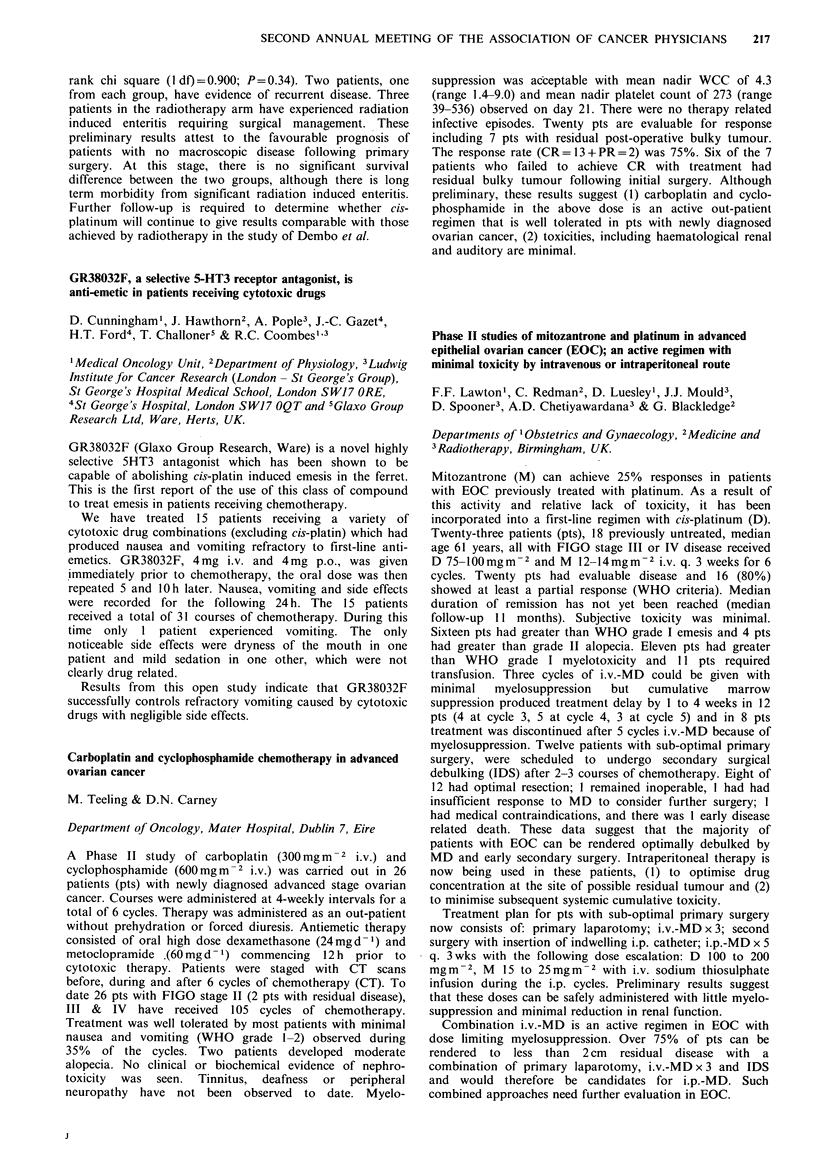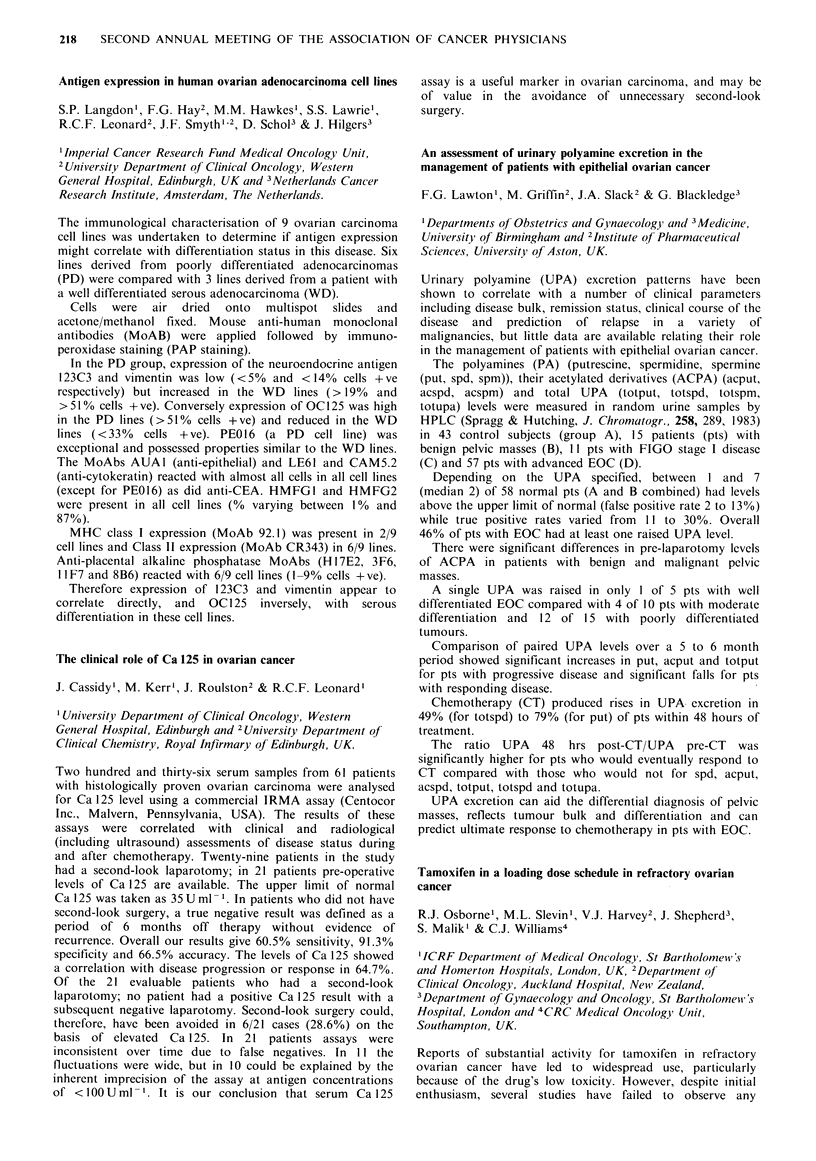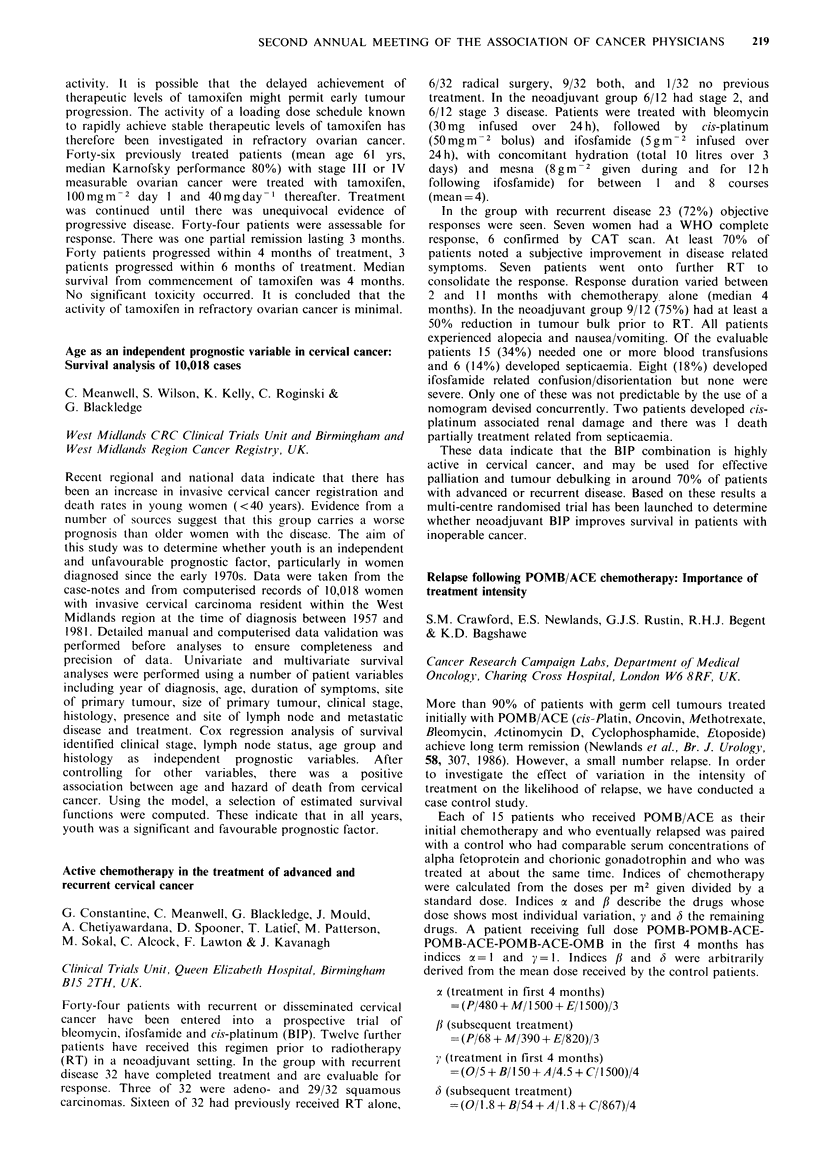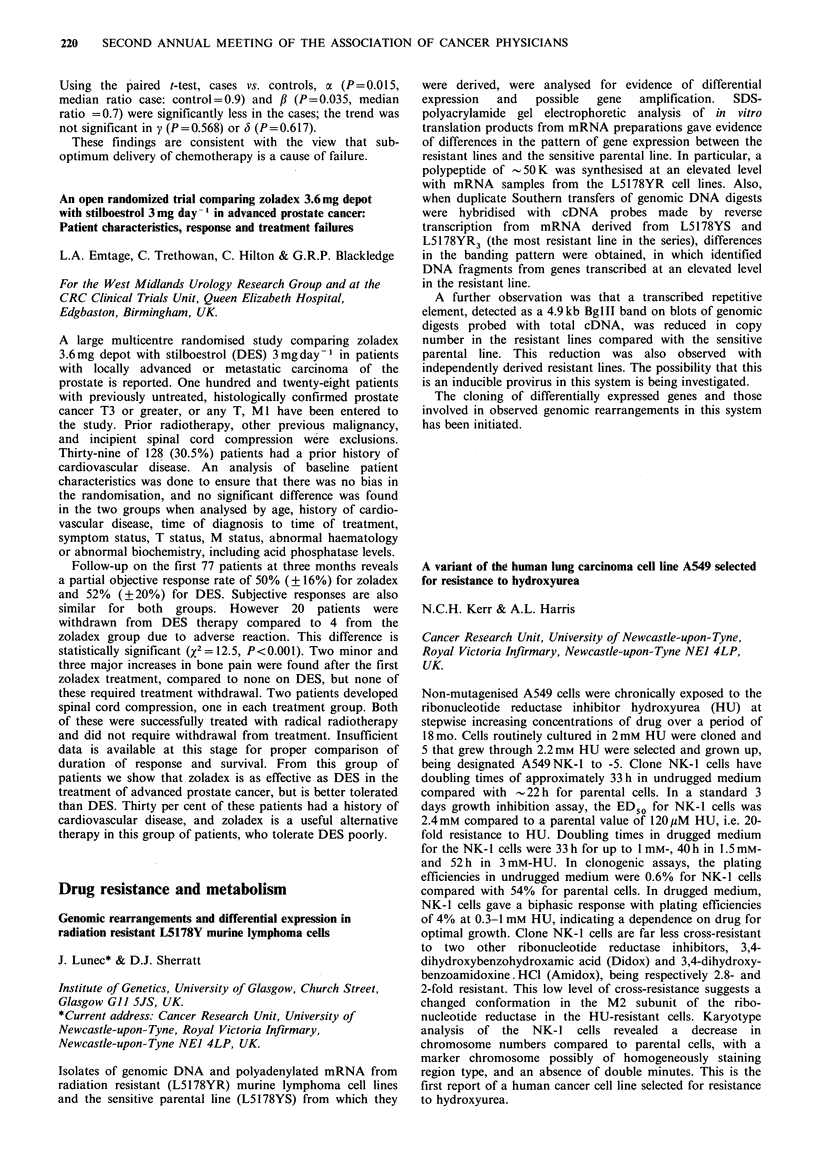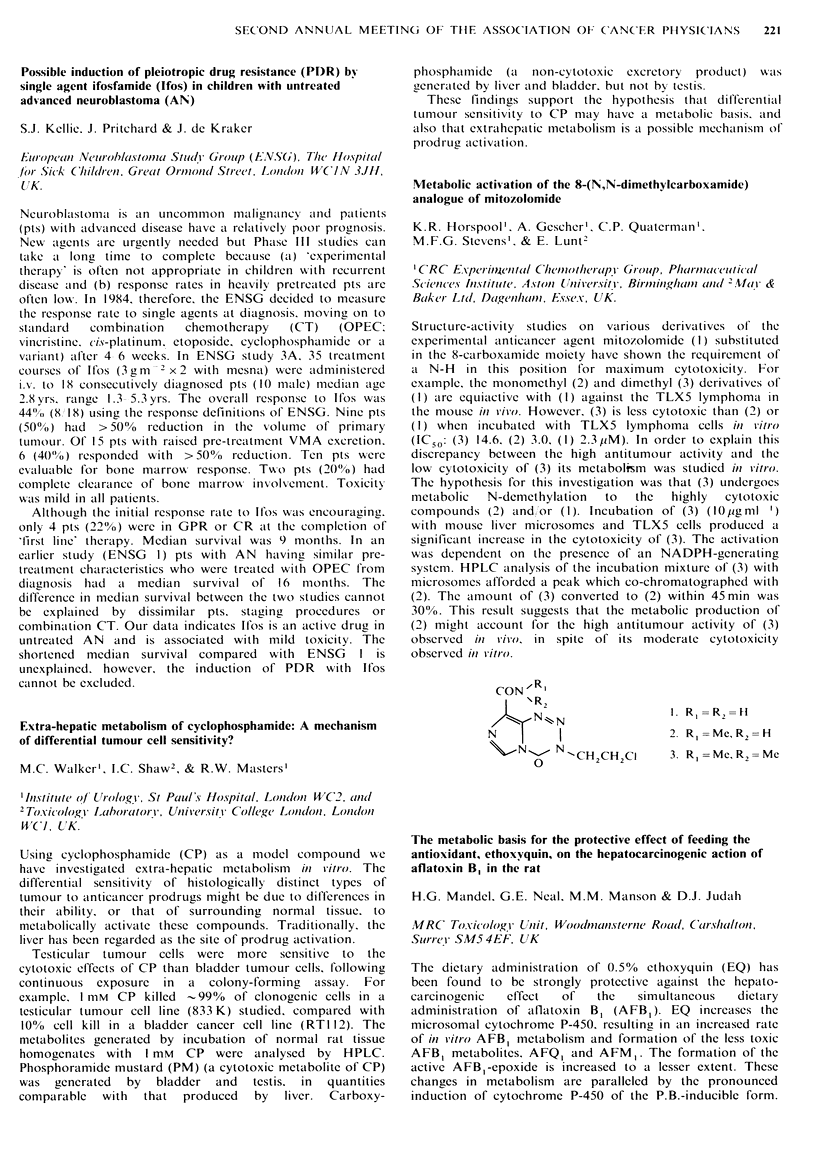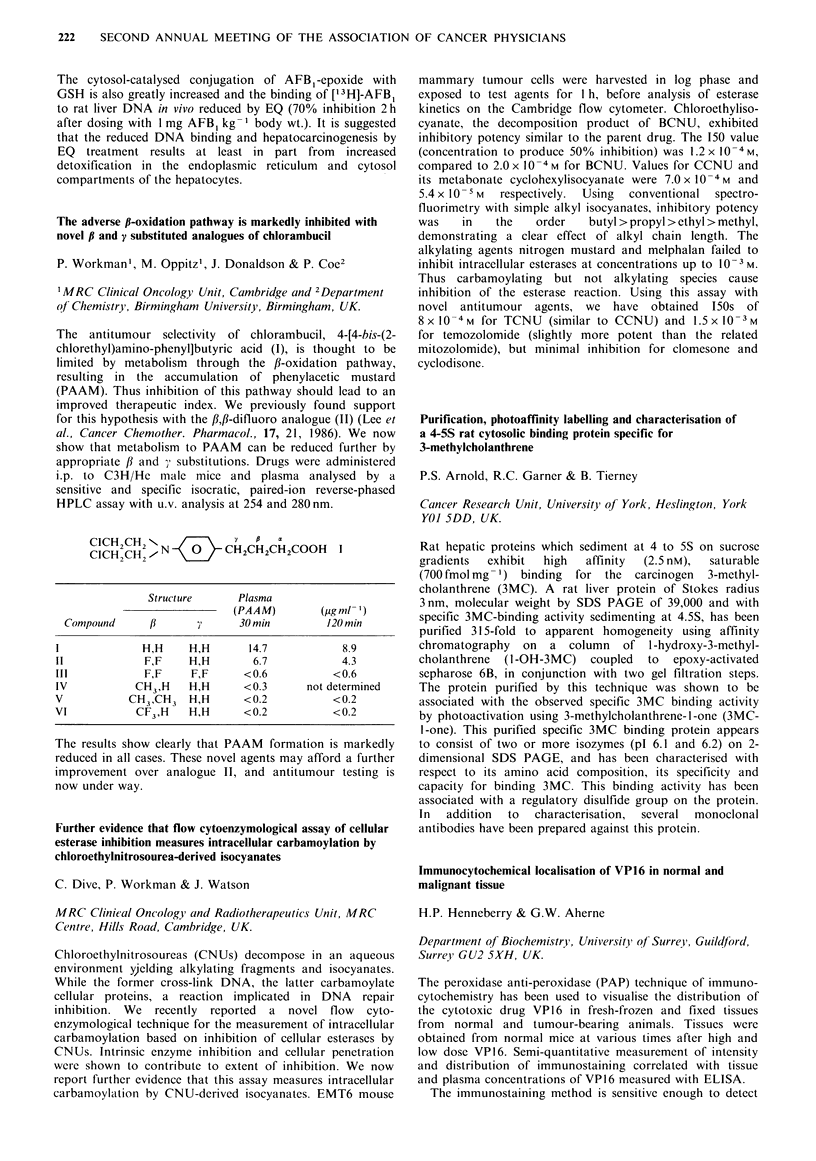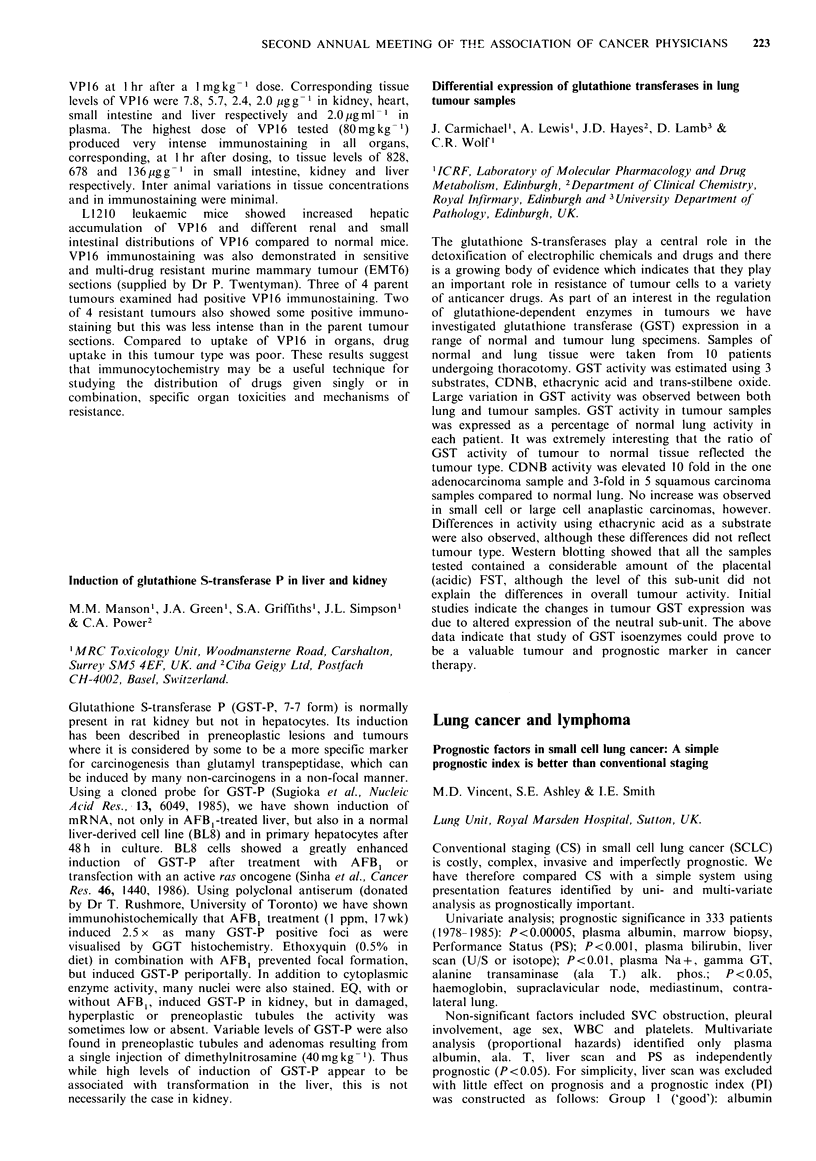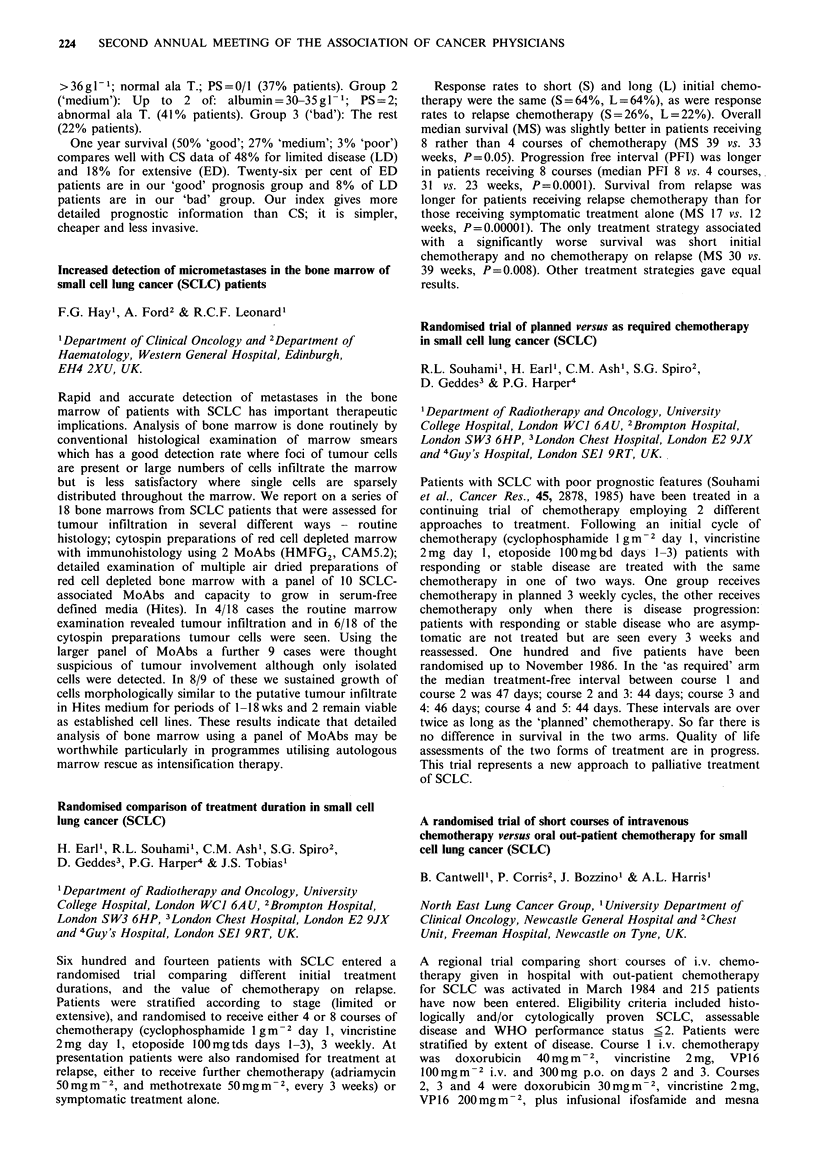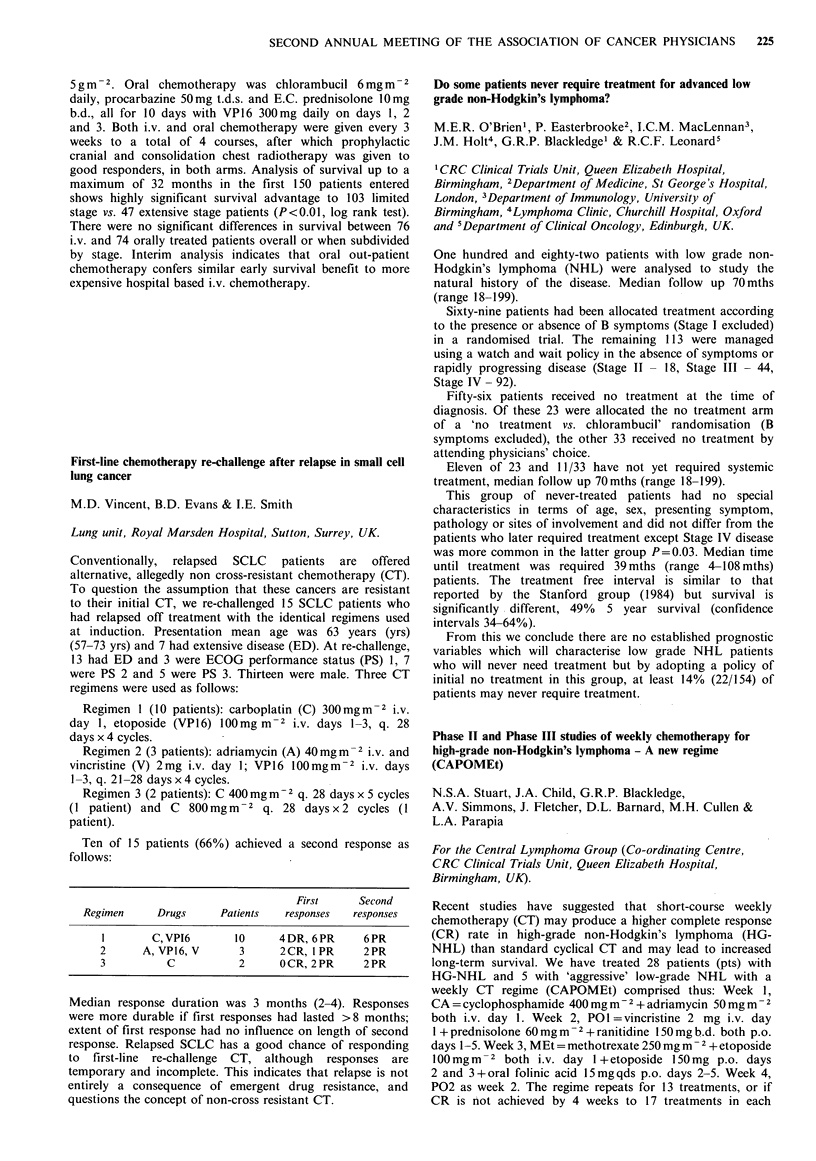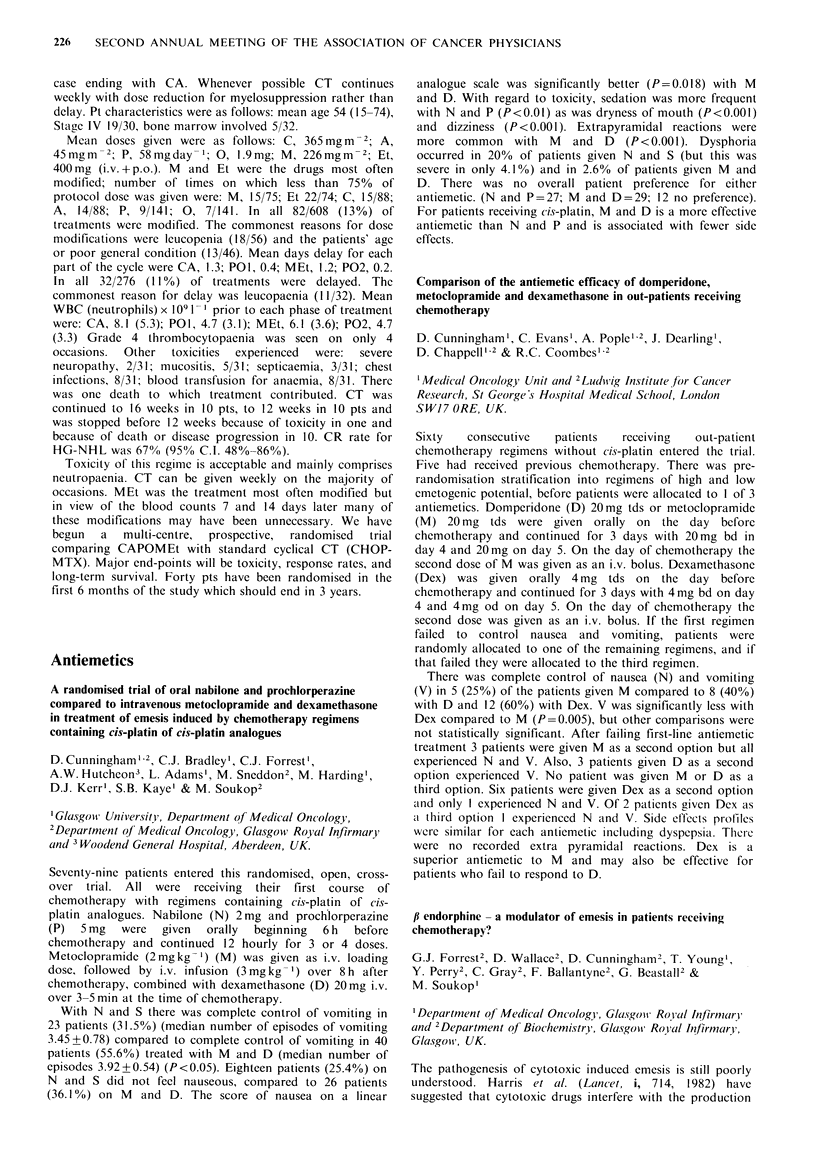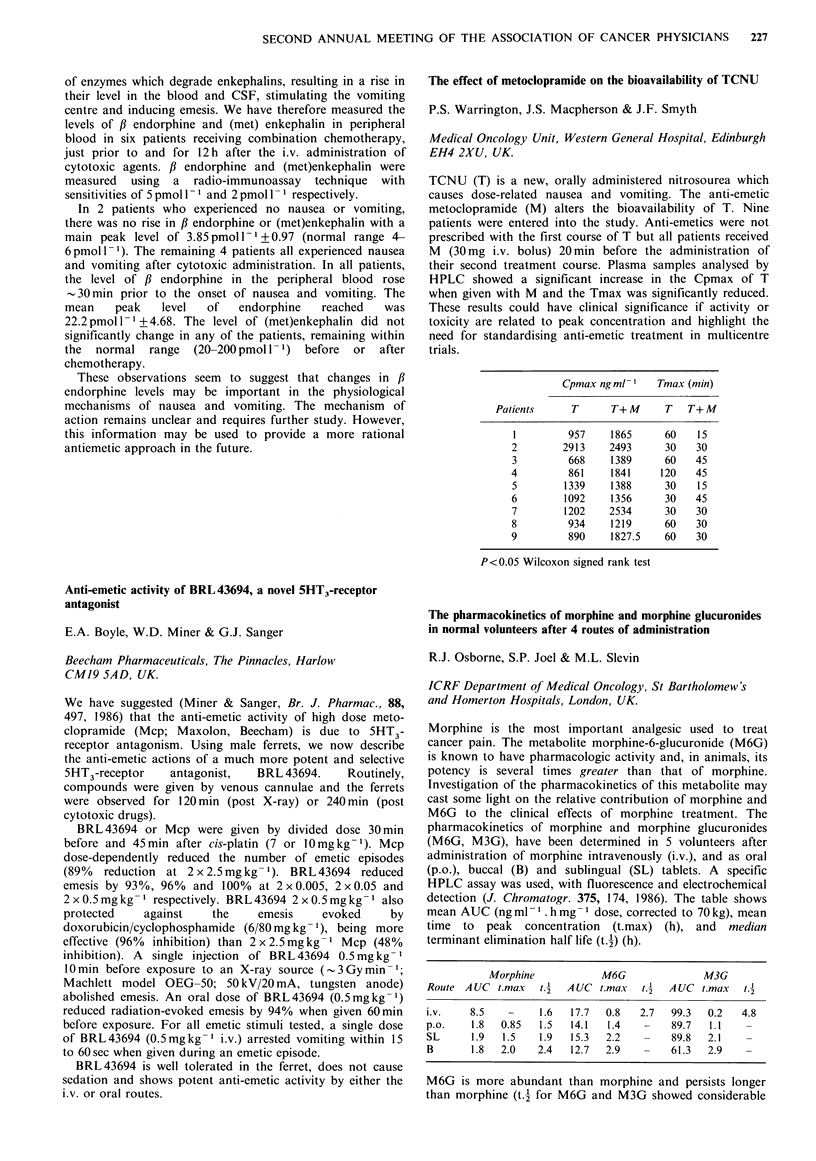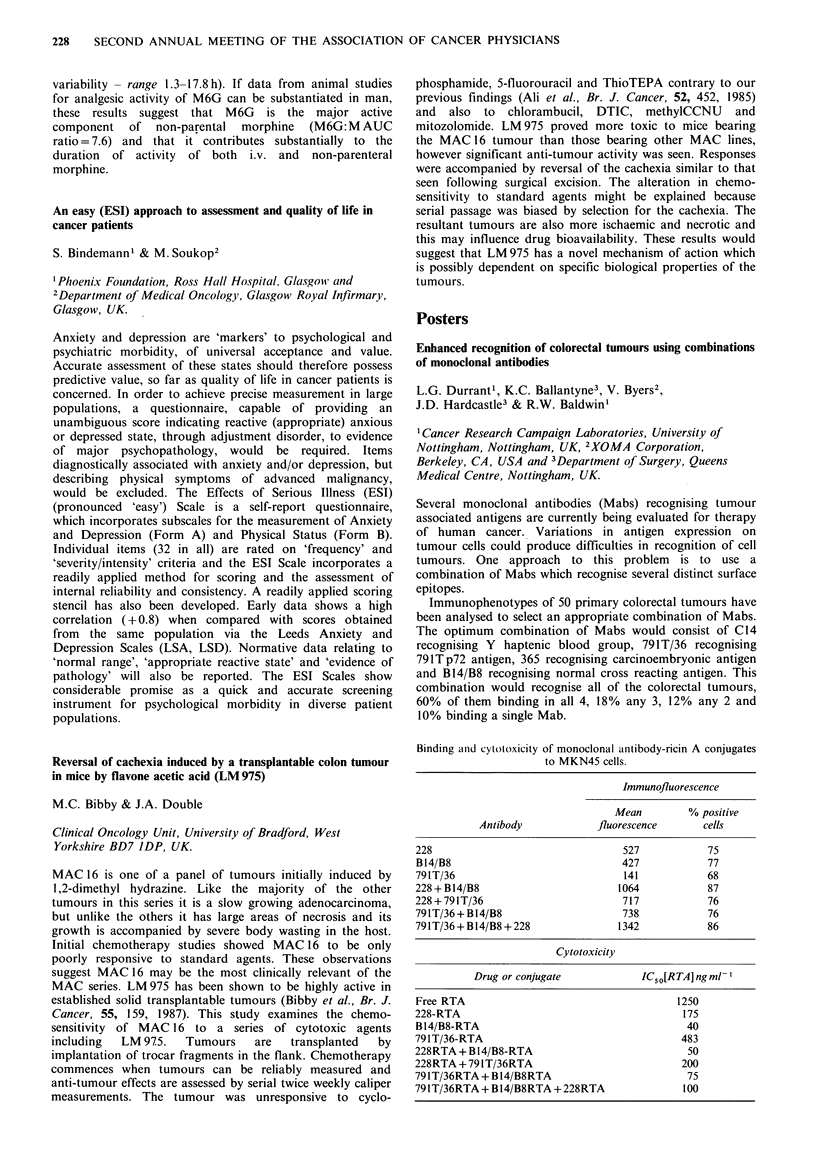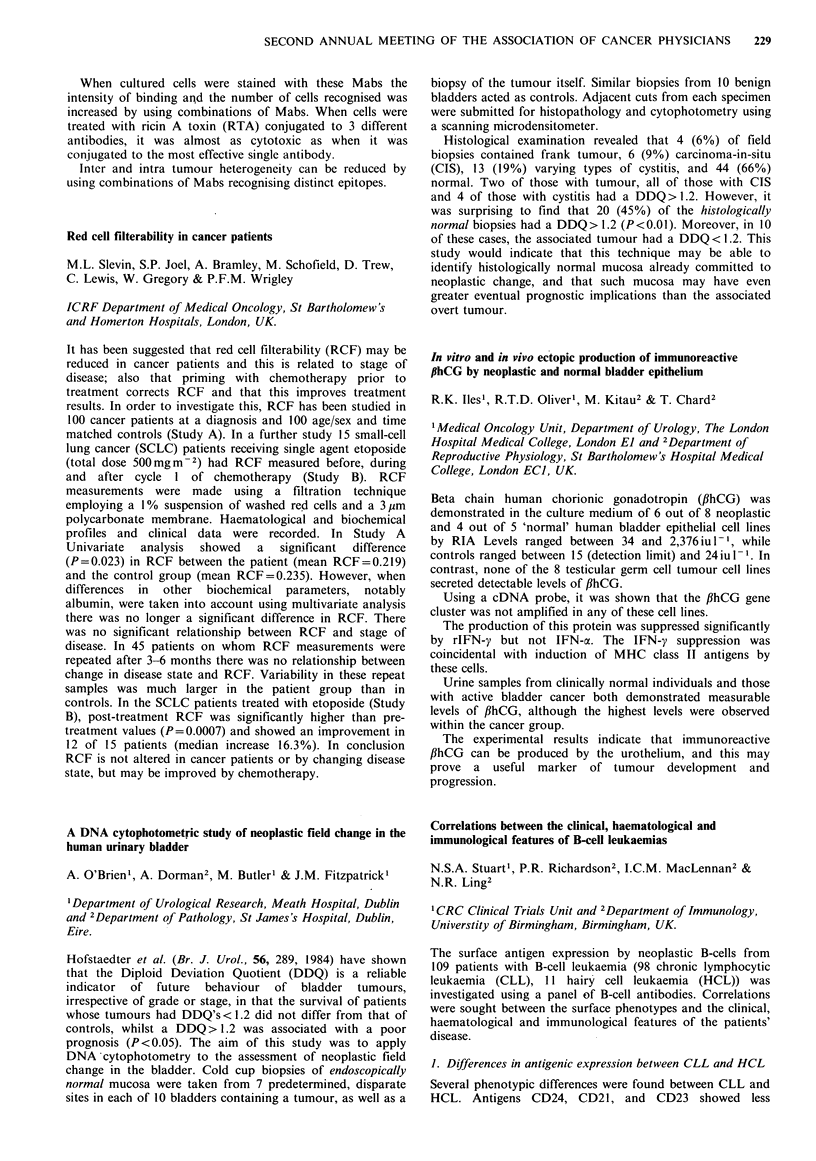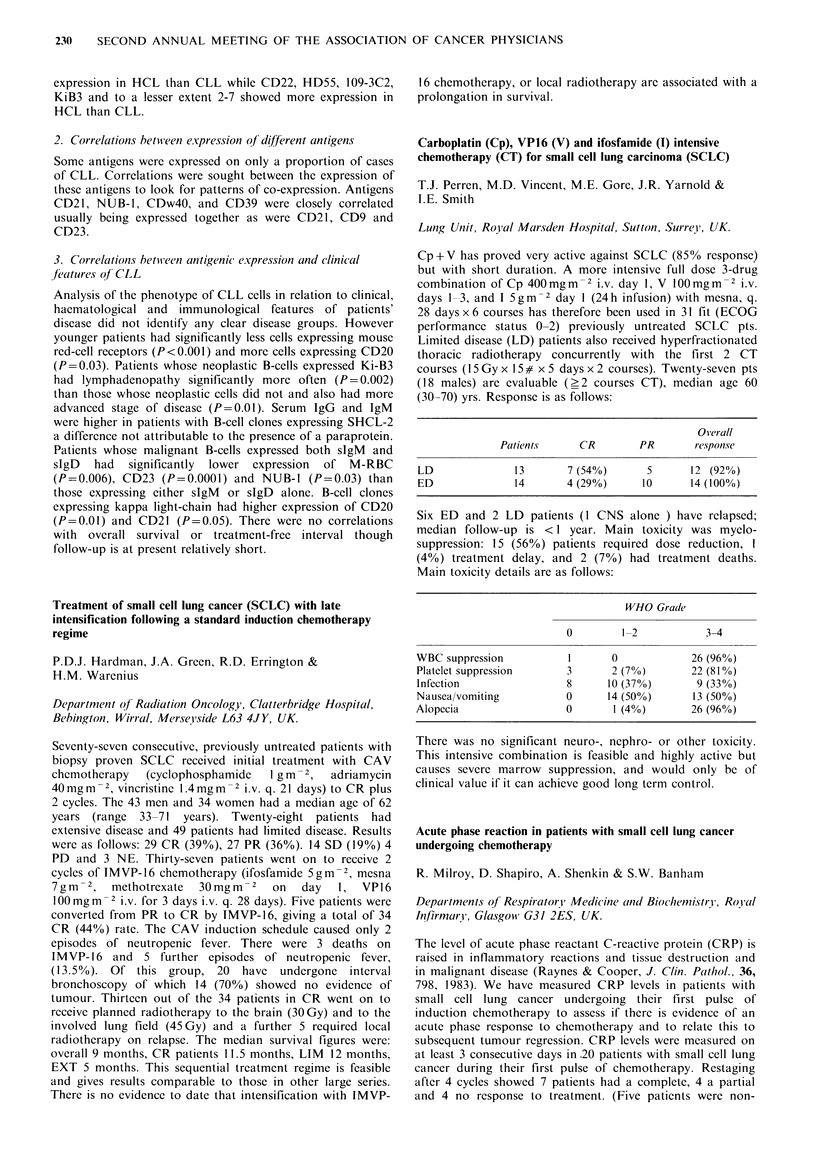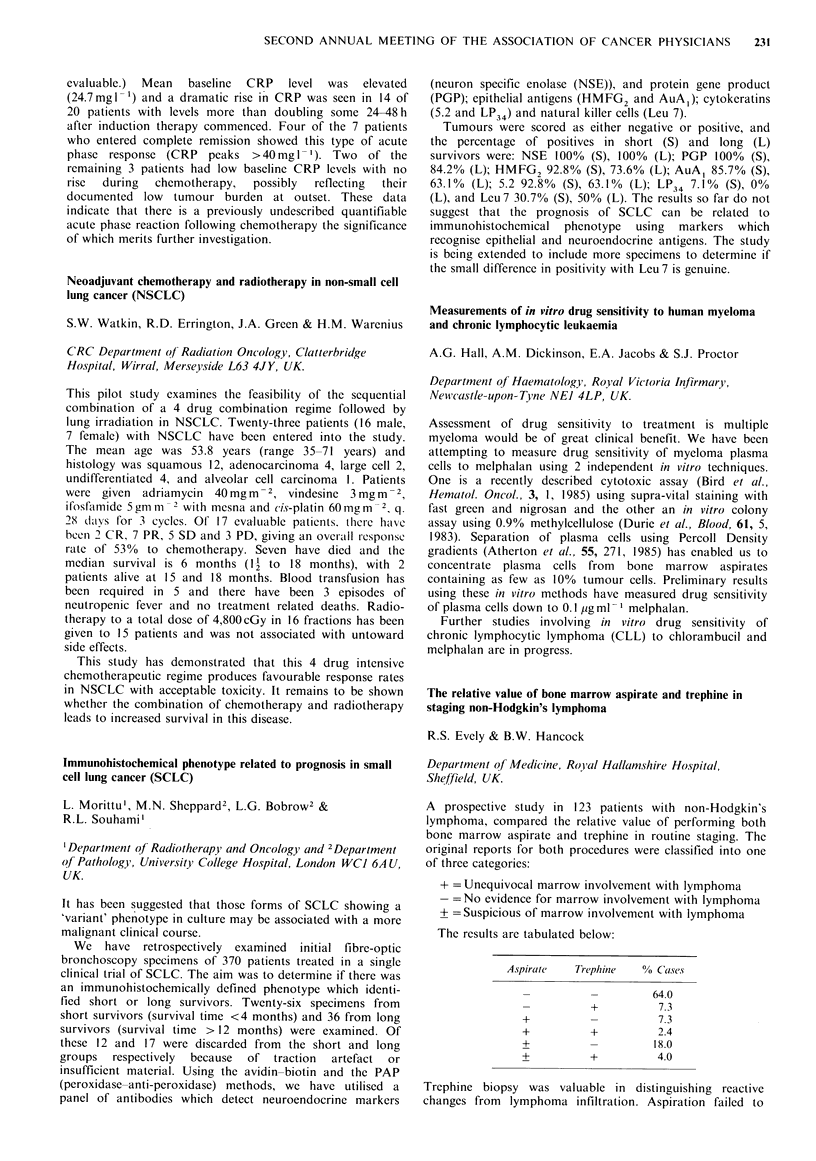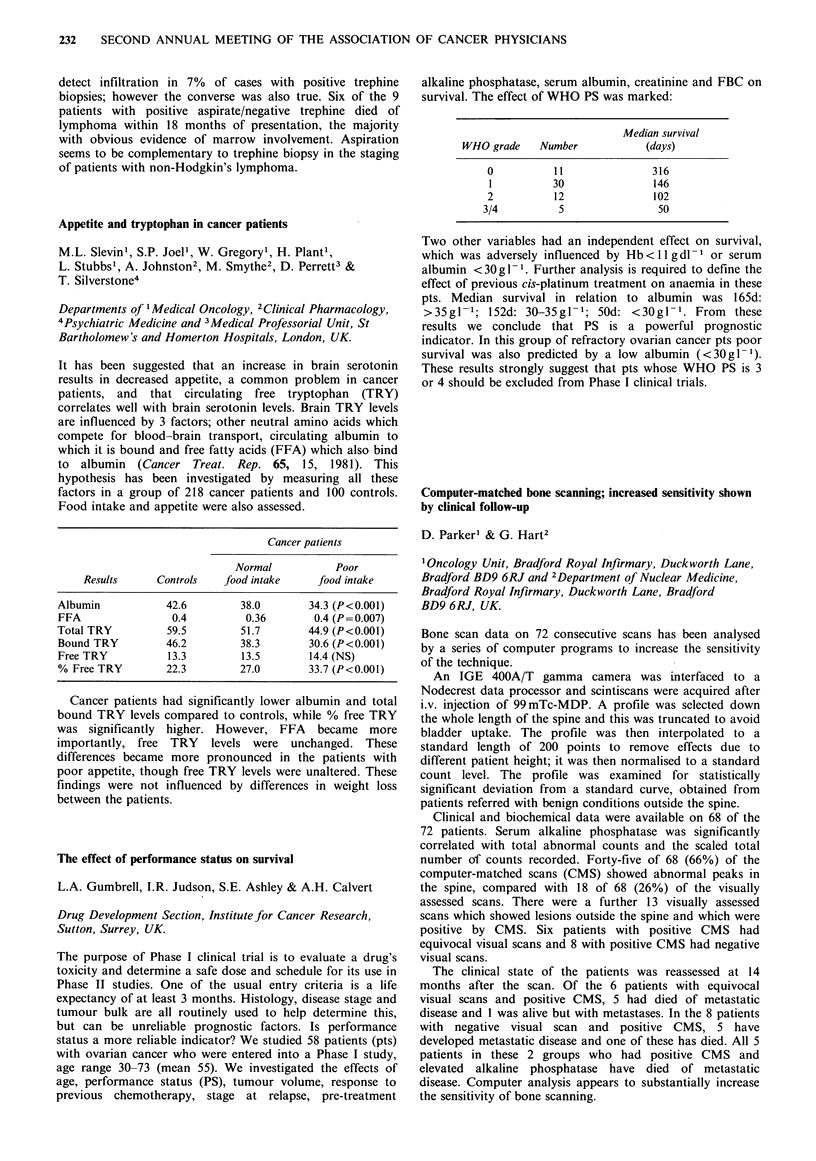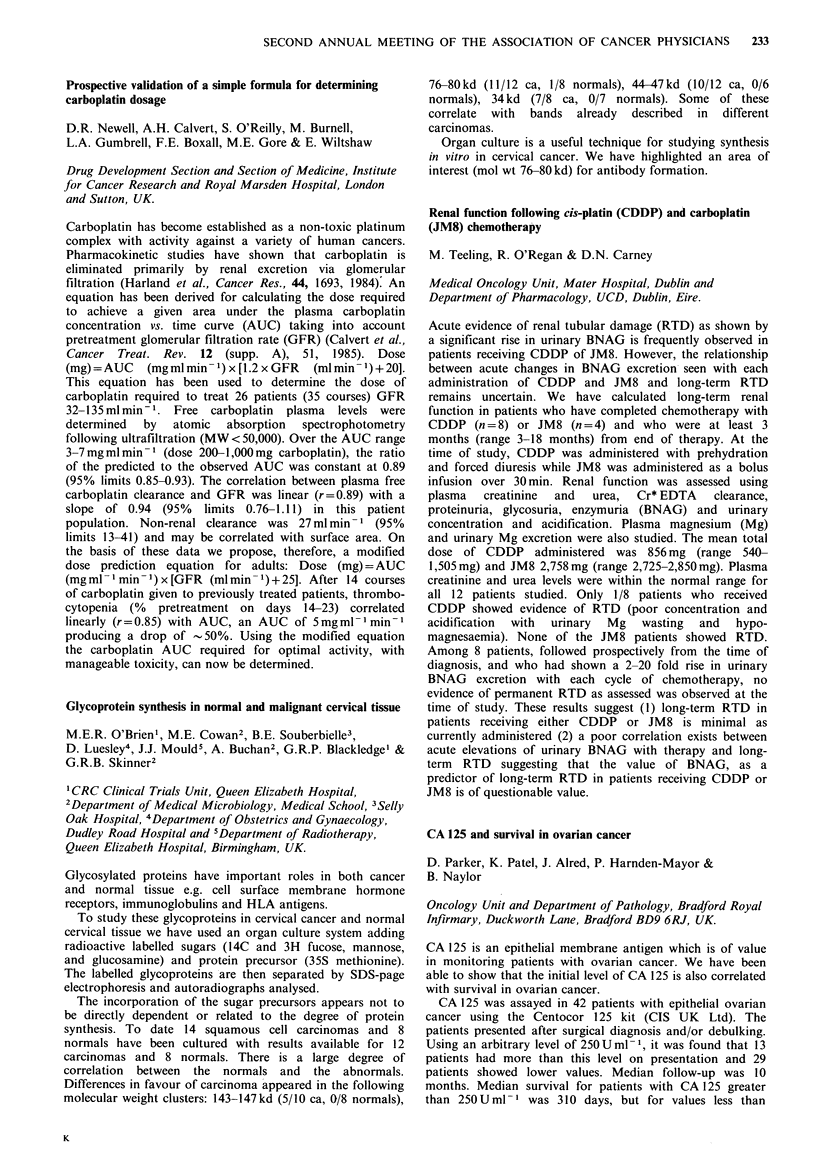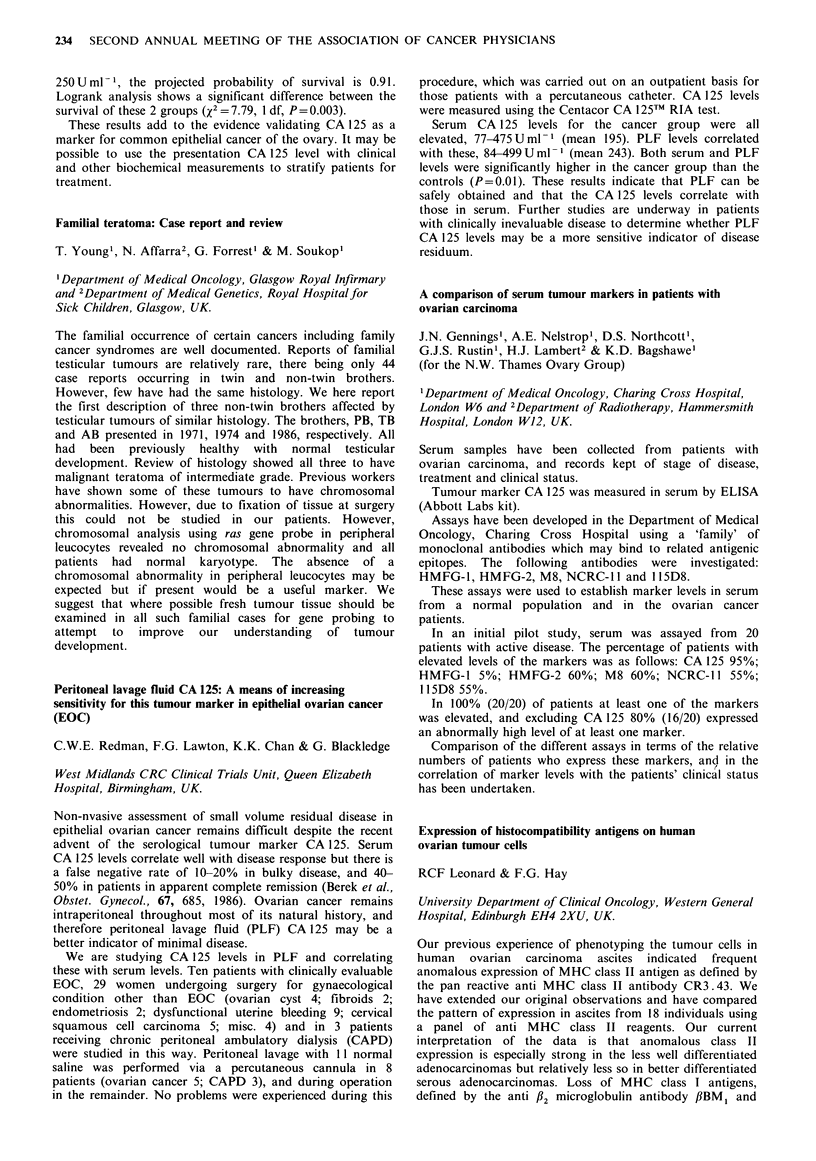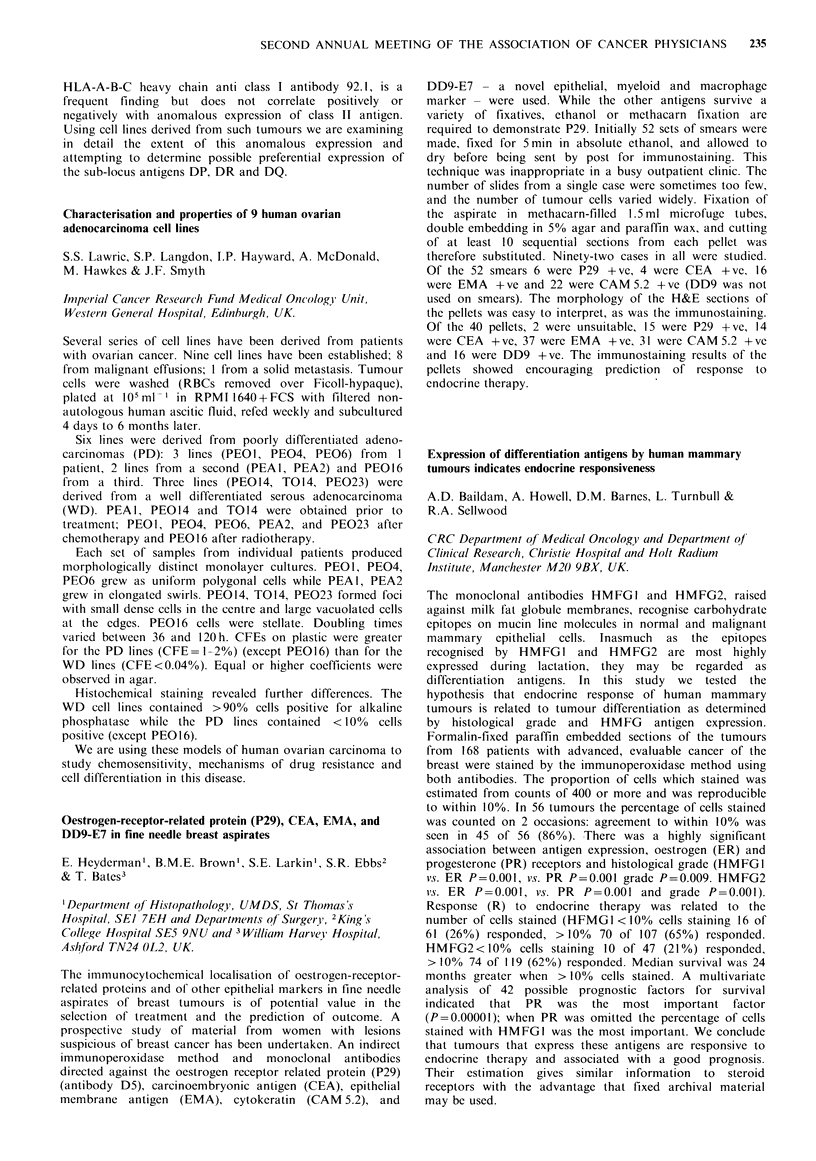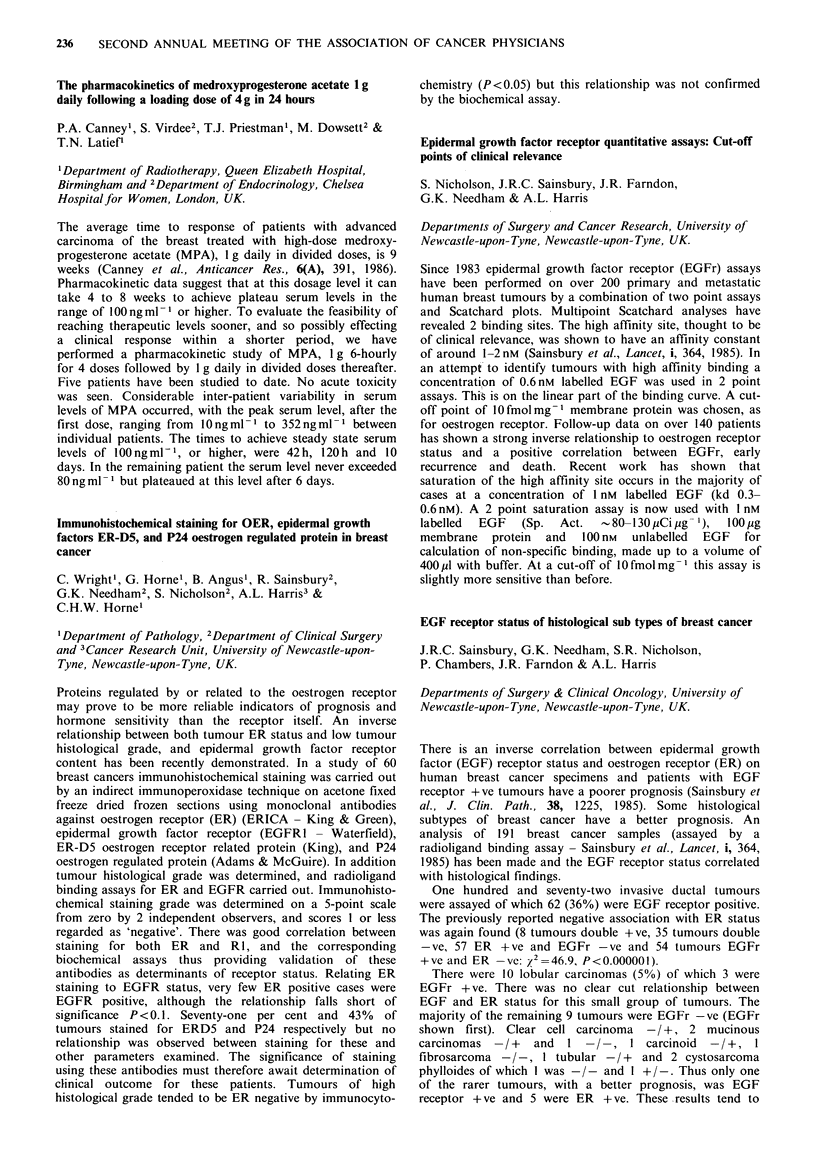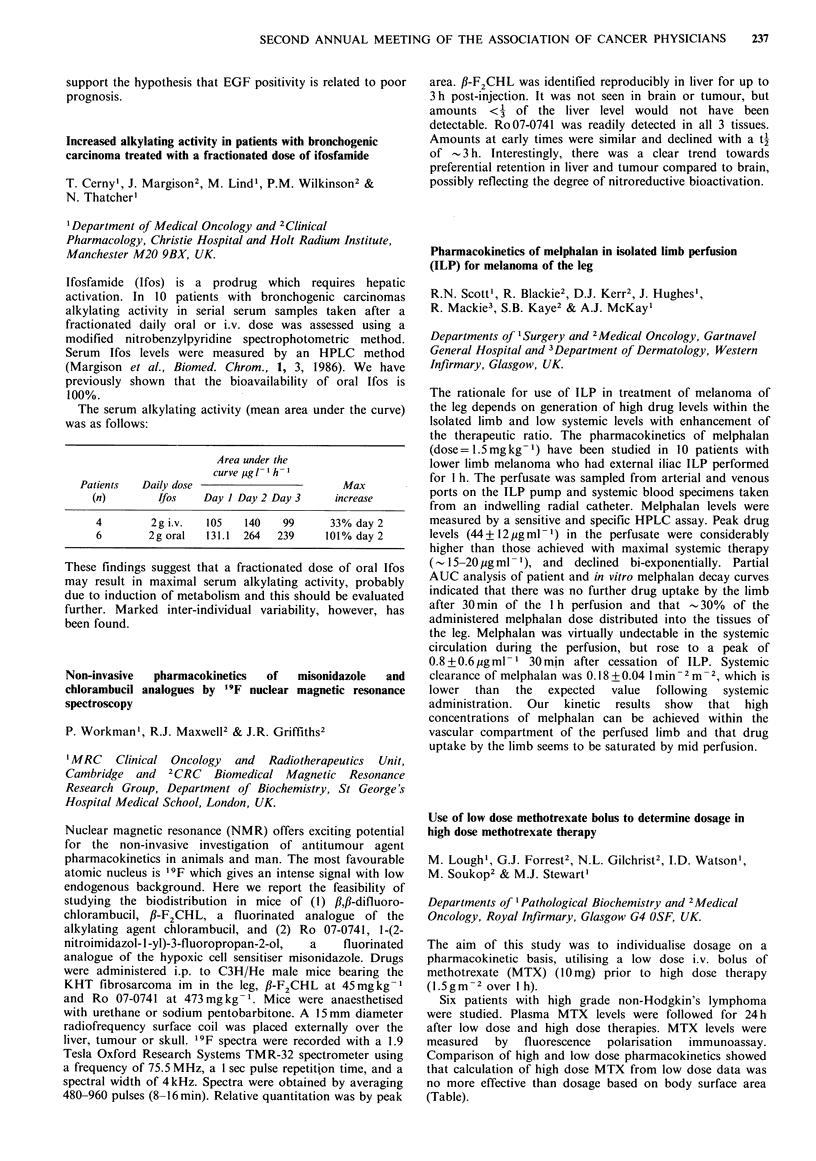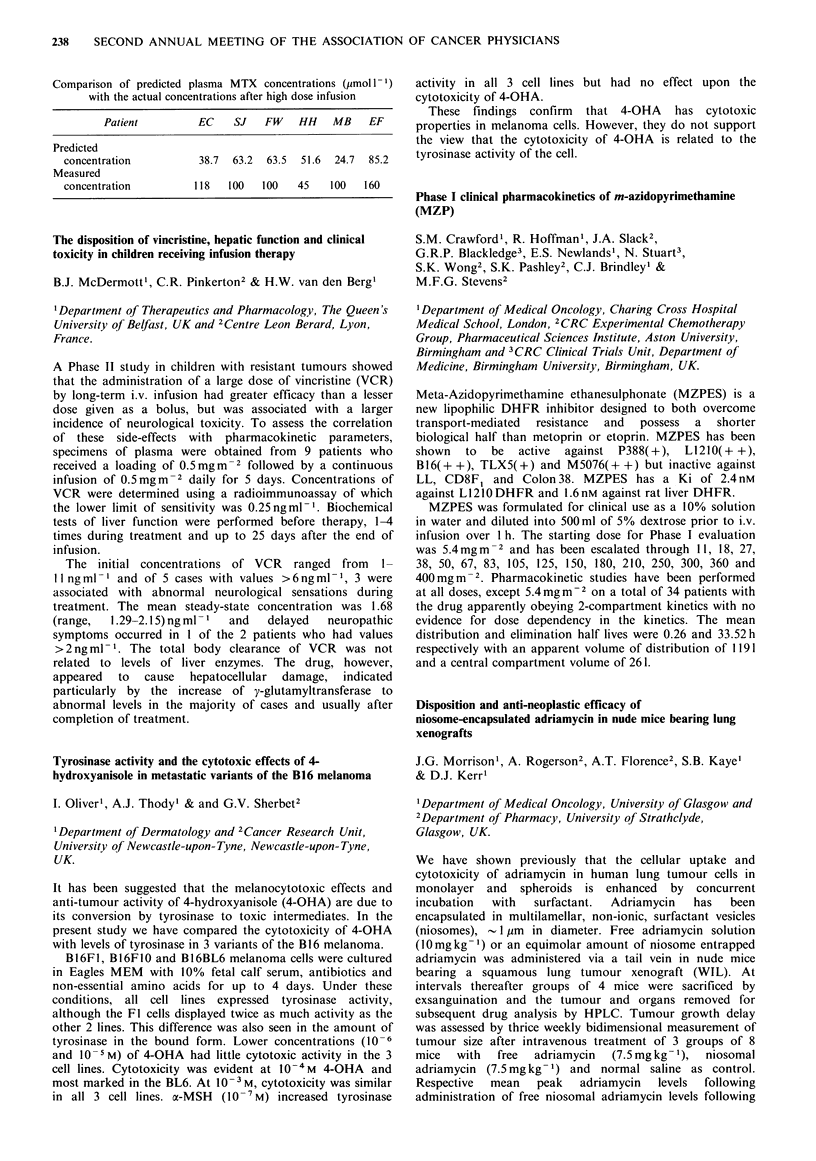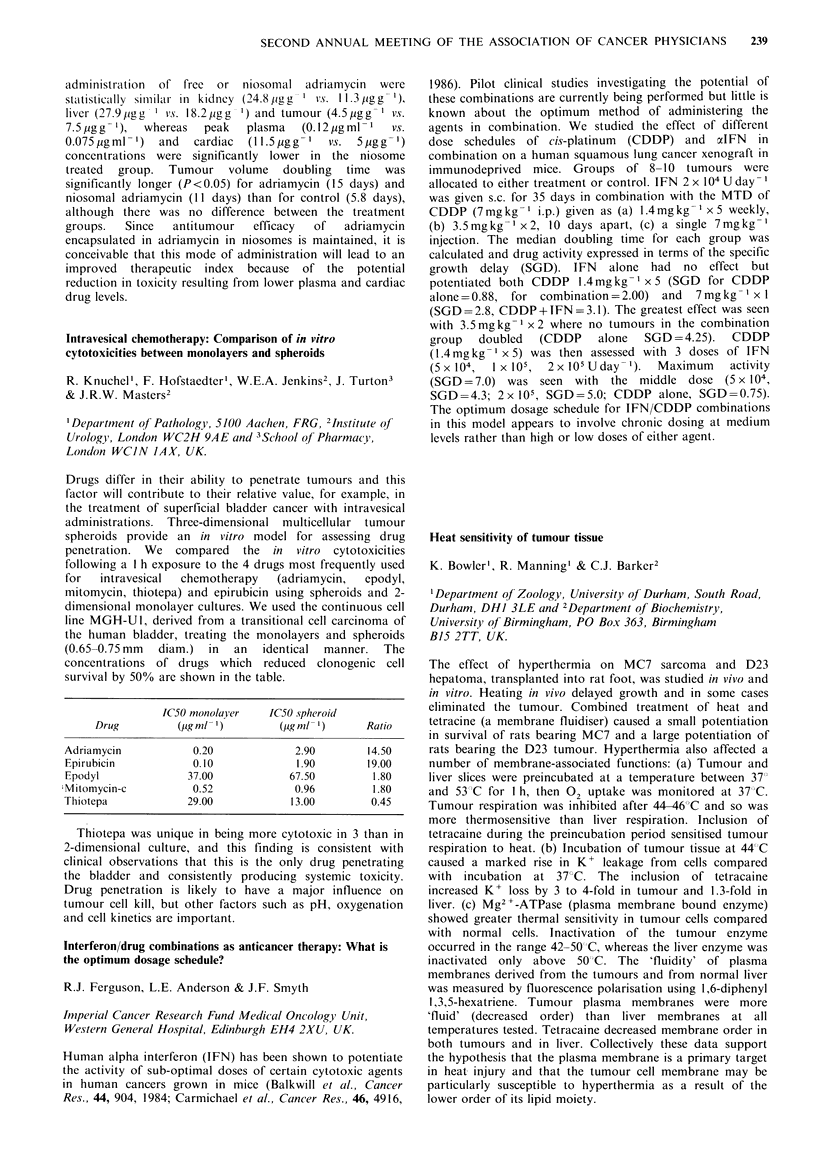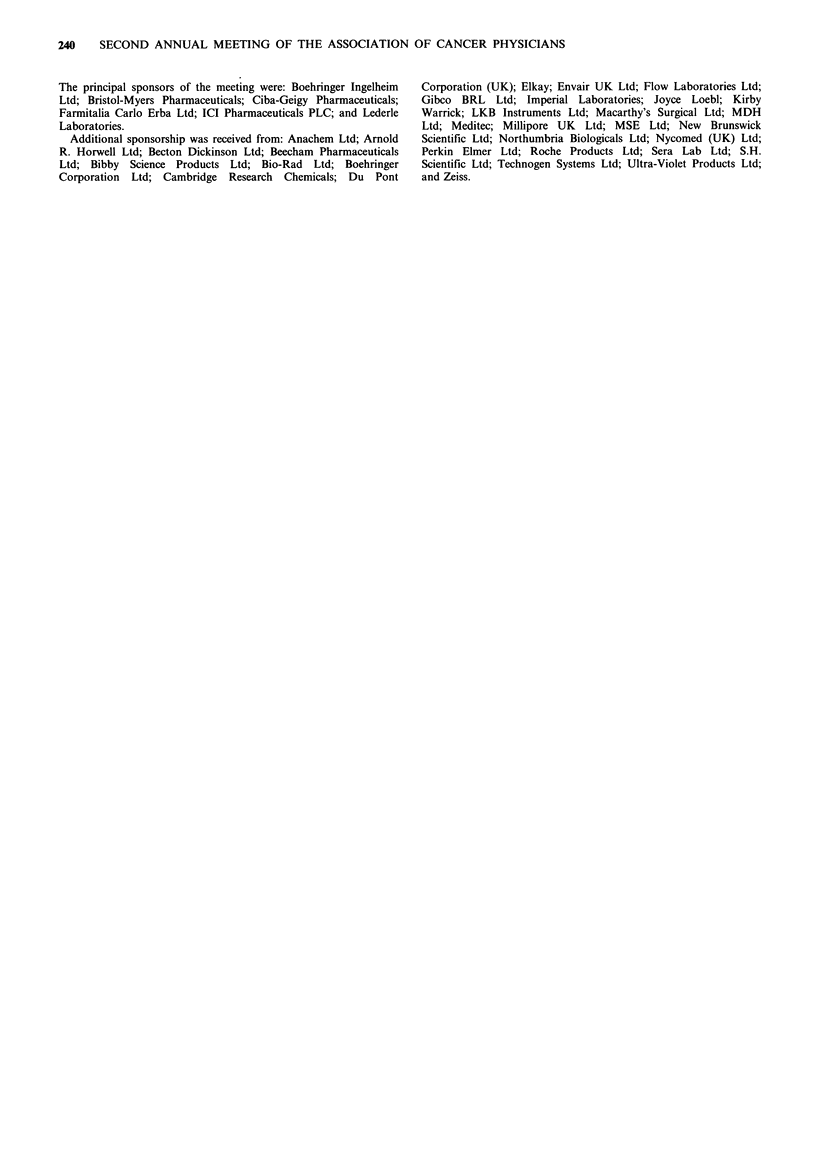# Second annual meeting of the Association of Cancer Physicians (in conjunction with the 28th AGM of the British Association for Cancer Research). April 6-8, 1987, Newcastle-upon-Tyne, UK. Abstracts.

**DOI:** 10.1038/bjc.1987.181

**Published:** 1987-08

**Authors:** 


Br. J. Cancer (1987), 56, 210–240 © The Macmillan Press Ltd., 1987

### Second Annual Meeting of the Association of Cancer Physicians (in conjunction with the 28th AGM of the British Association for Cancer Research)*

(Incorporating Symposia on 'Developments in Cancer Medicine' and 'DNA Repair'†)
April 6–8, 1987.

*Held at the University of Newcastle-upon-Tyne, UK.*

### Abstracts of Invited Papers‡

### Symposium on 'Developments in Cancer Medicine'

### Phase I and II drugs within the EORTC

J.G. McVie

*Netherlands Cancer Institute, Antoni van Leeuwenhoek Huis, 1066 CX Amsterdam.*

New drug development has been coordinated within the EORTC for the last 3 years. An office in Amsterdam oversees various stages of development from synthesis through formulation, toxicology, pharmacology to Phase I study. Task orientated groups conduct work on a contract basis culminating in Phase II disease orientated trials. Whereas the system has yet to throw up new active agents (as opposed to new analogues), the speed and efficiency of the process has been such that Europe can compete on equal terms with the USA. A platinum analogue such as JM40 for instance went through from bulk synthesis to Phase I trial in 11 months. There are currently a total of 200 drugs on file (subfiles are shared with the NCI and the CRC Phase I group, UK); 30 drugs in development, 10 drugs in Phase I and 8 drugs in Phase II. Standard documents have been published regarding guidelines for formulation, toxicology, and preclinical ,pharmacology in relation to judging Phase I starting dose, and master protocols in Phase I and Phase II trials. Recent developments have been the use of new routes of administration to test drugs such as intraperitoneal chemotherapy and the development of new protocols to test biological response modifiers.

### Clinical implications of the biology of lung cancer

N. Carney

*Mater Hospital, Dublin 7, Eire.*

Advances over the past decade in the staging and identification of prognostic factors in lung cancer have not been correlated with improvements in therapeutic approaches. However, the ease of establishment of cell lines of both small cell (SCLC) and non SCLC (NSCLC) has led to a greater understanding of the biological properties of these tumours. Studies of SCLC cell lines have revealed that the majority of these tumours express elevated levels of L-dopa decarboxylase (DDC); bombesin (BN); neuron-specific enolase (NSE) and creatine-kinase BB (CK-BB). In SCLC patients serum levels of both NSE and CK-BB show an excellent correlation with disease extent and response to therapy. Moreover, the finding of elevated NSE, BN and CK-BB in the CSF is highly suggestive of metastatic tumour in this site. Heterogeneity of the expression of DDC and BN is observed in SCLC cell lines. Variant SCLC lines (lacking DDC and BN) are associated with a more aggressive behaviour *in vitro* and *in vivo* and patients with variant SCLC have a shorter survival than those with classic SCLC. The development of numerous monoclonal antibodies has permitted their evaluation in the imaging and detection of tumour cells not detected by other means. Clinical trials are underway assessing their anti-proliferative activity *in vivo*. While considerable data on oncongene expression are available for cell lines, their clinical relevance still needs to be assessed, in particular the role of *myc* oncogenes in SCLC. Finally studies of NSCLC cell lines have revealed that 20% express biochemical features of SCLCL. Future trials in both SCLC and NSCLC will prospectively assess the value of tumour-endocrine properties in predicting response to therapy and survival.

### New considerations in the evaluation of staging in Hodgkin's disease

D. Crowther

*Department of Medical Oncology, Christie Hospital and Holt Radium Institute, Manchester M20 9BX, UK.*

Since the reports of the Ann Arbor Committee on staging in Hodgkin's disease in 1971 a number of developments have taken place. New staging procedures such as CT scanning and magnetic resonance imaging have been introduced to improve tumour detection and enable more accurate estimates of bulk and extent. Other investigations such as lung tomography, IVP, inferior cavography have become unnecessary in the light of these observations. The increased use of needle biopsy techniques and immunohistochemistry to detect disease in suspicious areas have improved the accuracy of staging. In addition, it has been recognised that features other than stage, as defined in the Ann Arbor Staging Classification are of prognostic importance and may be helpful in making treatment decisions. Features such as age, blood lymphocyte count, ESR, bulk of disease, number of nodal and extra nodal sites of involvement have been

*Enquiries to the ACP Secretariat, Department of Medical Oncology, Christie Hospital and Holt Radium Institute, Manchester, M20 9BX, UK.
†For abstracts see this issue pp. 173–209.
‡Reprints of these abstracts are not available – Ed.

shown to be of prognostic importance using multivariate analyses. A standard approach to pretreatment evaluation, taking into account all prognostic features, will facilitate communication and exchange of information. The role of subgrouping according to prognostic indices is not yet defined, but future analysis will be improved if known prognostic factors are recorded and subgrouping on this basis may be helpful in defining management policies.

### Developments in the chemotherapy of multiple myeloma

P. Selby

*Section of Medicine, Institute of Cancer Research, Royal Marsden Hospital, Sutton, Surrey, UK.*

Conventional chemotherapy produces improvement in some 50% of patients with multiple myeloma and prolongs median survival to two or three years. Complete remission of multiple myeloma is unusual and the disease is not curable. We have explored the use of high dose melphalan with and without the addition of methylprednisolone in the management of myeloma and found a complete remission rate of almost 30% and the majority of other patients obtain partial remissions. The quality of their remission seems excellent but unfortunately the median duration of complete remission is only 19 months in both of these studies. In our present study we are exploring initial chemotherapy with vincristine and adriamycin by 4-day infusion together with high doses of methylprednisolone. Patients who respond sufficiently to clear most of the myeloma from their bone marrow are then treated with high dose melphalan and autologous bone marrow grafting. This programme is feasible and ABMT considerably reduces the duration and severity of the toxicity from high dose melphalan. The ultimate therapeutic advantage will only be assessed after prolonged follow up. The clinical results to date and parallel laboratory studies on the assessment of residual disease and the biology of myeloma will be presented.

### Combined modality approaches for the treatment of epithelial cancer of the ovary (EOC)

G. Blackledge, F. Lawton, C. Redman, D. Luesley, J. Mould, N. Stuart & K.K. Chan

*West Midlands Ovarian Cancer Group (WMOCG), West Midlands CRC Clinical Trials Unit, Queen Elizabeth Hospital, Birmingham, UK.*

Recent randomised trials of chemotherapy have failed to show long term survival benefit for *cis*-platinum based combination regimens compared with single alkylating agent treatment. Whilst second look laparotomy can be of prognostic importance, no study has shown any therapeutic benefit from second operations. Radiotherapy may benefit some patients with no macroscopic disease after first surgery, but studies show that radiotherapy given after chemotherapy does not confer any survival benefit, and may be detrimental to some patients.

In view of these findings, it might be suggested that routine therapy for advanced EOC should consist of initial surgery followed only by single alkylating agent chemotherapy, accepting that no improvements in survival can be made, and thus minimising toxicity in a palliative situation.

The WMOCG has been carrying out a series of studies to determine whether combined modality approaches might offer benefit to some sub-groups of patients with EOC. These studies have centred on three questions: (a) can a short induction course of intensive chemotherapy adequately debulk EOC? (b) can the integration of very early second surgery improve the prognosis for patients with bulky disease? and (c) can the management of adequately debulked EOC be improved?

Initial chemotherapy studies using first alternating chemotherapy, and then 3 courses of *cis*-platinum combination therapy followed by alkylating agent, suggested that chemical debulking could be achieved in ~80% of cases with only 3 courses of *cis*-platinum based treatment. After 2 or 3 courses second surgery (intervention debulking surgery – IDS), could be carried out resulting in no macroscopic or <1 cm disease in 80% of cases. The potential for further effective therapy in these cases exists.

This experimental approach is now being tested prospectively in a randomised trial, comparing two chemotherapy regimens, and also randomising patients with bulky disease after primary surgery to IDS or no IDS. Fifty patients have so far been recruited in 10 months, and 31 of these are eligible for randomisation to further surgery or not.

In patients who have been optimally debulked at first operation, the potential of intraperitoneal therapy is being investigated following systemic therapy. Combinations of active but less toxic drugs offer the potential for long term survival in this group, without concomitant toxicity.

In patients with no macroscopic disease following primary surgery, a randomised trial of abdominal radiotherapy *versus cis*-platinum chemotherapy is being carried out. Preliminary results suggest that active chemotherapy is as good as radiotherapy in this setting, with less long term toxicity.

The identification of patient sub-groups in EOC has meant that potential improvements in management can be applied to these groups and any genuine improvement in survival will not be masked by the whole population.

### Abstracts of members' proffered papers

### Breast cancer

### Expression of oestrogen receptor mRNA in human breast cancer biopsies

P.J. Barrett-Lee, M.T. Travers, T.A. McDonnell & R.C. Coombes

*Ludwig Institute for Cancer Research, St George's Hospital Medical School, Cranmer Terrace, London SW17 0RE, UK.*

Oestrogen receptors (ER) can be detected in over 50% of human breast cancers either by the dextran coated charcoal (DCC) assay, or using specific monoclonal antibodies (ERICA). Determination of ER status is important in optimal treatment planning since ~70% of these ER-positive tumours respond to endocrine therapy compared with <5% of ER-negative tumours. In order to obtain an improved predictive test, we have examined the levels of transcripts for ER in tumour tissue.

Levels of ER mRNA were determined in 19 primary breast cancers using a 1.3 kilobase (kb) ER cDNA clone. Dot blot hybridisation analysis of total RNA extracted from these tumours and subsequent densitometry allowed quantification of ER mRNA levels. These were compared with results obtained from the ERICA and DCC assays in each case.

Six of 19 tumours had no detectable ER mRNA and 5 of these were ER-negative (DCC < 10). Thirteen of 19 tumours demonstrated significant expression of ER mRNA and 12 of these were ER-positive (DCC > 10). One tumour with a DCC < 10 showed high levels of ER mRNA. However the ERICA in this case was strongly positive. Overall there was a positive correlation between ER mRNA and DCC assay (rs 0.56, $P < 0.02$) and a stronger correlation with ERICA (rs 0.70, $P < 0.001$).

These results show that both ERICA and DCC assays correlate with levels of ER mRNA and that ER-negative tumours produce no measurable ER mRNA. We are currently investigating the quantitative relationships between different mRNA's and subsequent response to endocrine therapy.

### Relationship between breast adipose tissue 17B hydroxysteroid dehydrogenase activity and response of breast cancer to systemic therapy

J.S. O'Neill & W.R. Miller

*Department of Clinical Surgery, University of Edinburgh, Royal Infirmary, Edinburgh EH3 9YW, UK.*

Recent investigations have suggested that steroid metabolism in breast adipose tissue may play a significant role in the natural history of breast tumours (O'Neill & Miller, *J. Endocrinol.*, **108** (suppl.), 106, 1986). In this study, 17B hydroxysteroid dehydrogenase (17BOHSD) activity has been measured in breast adipose tissue obtained from 16 patients with breast cancer immediately following systemic treatment (7 received endocrine therapy, 9 chemotherapy). The aim of the study was to relate enzyme activity (monitored by the conversion of androstenedone to testosterone) to the response to therapy. Complete responses to therapy were seen in 3 patients and partial responses in 9. Four patients failed to respond. Significantly higher 17BOHSD activity was found in non-responders (median 4.1 pmol testosterone $mg^{-1}$ protein $h^{-1}$ (units)) than in responders (median 0.8 units ($P < 0.02$ by Wilcoxon rank test)). Particularly low levels occurred in adipose tissue from patients who achieved a complete clinical and pathological remission. These results provide clear evidence of an interaction between breast cancers and the tissues in which they develop. Whether the level of enzyme activity in adipose tissue adjacent to tumours is influenced by tumour presence or is an indicator of the responsiveness of a tumour to therapy remains to be determined.

### Fibroblasts stimulate the growth of MCF7 human breast cancer xenografts

K. Horgan, D. Jones, & R.E. Mansel

*Department of Surgery, University of Wales College of Medicine, Heath Park, Cardiff CF4 4XN, UK.*

Stromal factors have been implicated in the regulation of mammary epithelial cell growth in the rodent but their influence on human breast cancer is unknown (Durnberger & Kratochwil, *Cell*, **19**, 465, 1980). Fibroblasts were grown in monolayer cell culture from explants of benign and malignant breast tissue and normal skin. MCF7 human breast cancer cells alone ($2 \times 10^6$ cells) or in combination with live breast or skin fibroblasts (MCF7 + BF, MCF7 + SF) were inoculated into the mammary fat pads of female nude mice. After 5 weeks tumours were excised, weighed and fixed for histological examination. The results are shown below.

| | *MCF7 alone* | *MCF7 + BF* | *MCF7 + SF* |
|---|---|---|---|
| tumours retrieved | $52/106^a$ | $127/135^b$ | $18/19^b$ |
| tumour weight (mg) | $41 \pm 4^c$ | $174 \pm 11^d$ | $139 \pm 16^d$ |

$^a$*vs.* $^bP < 0.001$ Chi square; $^c$*vs.* $^dP < 0.001$ Mann-Whitney.

Addition of live fibroblasts to MCF7 inocula significantly increased the number and growth of subsequent tumours. Tumour formation was not observed after injection of fibroblasts alone. Histological examination did not reveal any obvious differences between the tumour groups.

These experiments demonstrate a highly stimulatory role for fibroblasts in inducing human breast cancer tumorigenesis in nude mice.

### PCM42 xenografts: A new experimental model to study human breast cancer differentiation

K. Horgan, D. Jones & R.E. Mansel

*Department of Surgery, University of Wales College of Medicine, Heath Park, Cardiff CF4 4XN, UK*

PMC42 is a unique human breast carcinoma cell line that differentiates both morphologically and functionally *in vitro* and is stimulated by prolactin and EGF (Whitehead *et al.*, *J. Natl Cancer Inst.*, **70**, 649, 1983). Previous efforts to transplant PMC42 cells from monolayer culture into nude mice have failed, but fibroblasts have recently been shown to stimulate other human breast cancer xenografts.

PMC42 cells alone (2–4 $\times 10^6$ cells) or in combination with human breast fibroblasts (PMC + F) were inoculated into the mammary fat pads of female nude mice. After 6–8 weeks the mice were sacrificed and the mammary fat pads excised and fixed for serial histological sectioning.

The results showed no tumour formation from 16 injections of PMC42 alone while 26 or 36 PMC + F inocula were successful. Histological examination demonstrated marked cellular organisation with many tubules or ductulo-alveolar like structures. Ultrastructural features included luminal microvilli characteristic of secretory cells, a basement membrane and inter-cellular junctions. The xenograft PMC42 stained strongly with monoclonal antibodies against human milk fat globule membrane while *in vitro* cultures were negative.

We conclude that the addition of fibroblasts allows growth of PMC42 xenografts in nude mice providing a new and valuable experimental model for the investigation of breast cancer differentiation and hormonal response.

### The effect of tamoxifen upon progesterone receptor (PR) concentrations and of cyclical endocrine therapy with tamoxifen (T) and medroxyprogesterone acetate (MPA) in patients with advanced cancer of the breast

H. Anderson, A. Howell, D.B. Smith, R.A. Cowan, D.M. Barnes & R.N.L. Harland

*CRC Department Medical Oncology & Department of Clinical Research, Christie Hospital and Holt Radium Institute, Manchester M20 9BX, UK.*

In the human mammary tumour cell lines, MCF-7, T stimulates PR synthesis because of a partial oestrogen agonist effect. The aim of this study was to determine whether an effect of T on PR synthesis could be shown in human mammary tumours *in vivo* and to test the effectiveness of cyclical endocrine therapy which might capitalise on the T effect. PR was measured in biopsies taken (a) before and after a median of 13 days T in 52 patients with advanced disease and (b) after a median of 7 days T in 58 patients with primary disease: controls (C) were 51 with primary disease who had 2 biopsies 7 days apart, but no T. Mean PR ($\pm$s.e.) in those with higher PR in the second biopsy was (a) $237 \pm 78$, (b) $318 \pm 135$ and (c) $71 \pm 21$ fmol mg$^{-1}$ cytosol protein (a *vs.* c $P = 0.03$, b *vs.* c $P = 0.03$). Nineteen of 21 (90%) advanced disease patients with a higher PR in the second biopsy subsequently responded to continued tamoxifen. These data suggest that T has agonist activity *in vivo* in responsive tumours and thus the effect of cyclical T 7 days, MPA 14 days, nil 7 days etc. ($n = 41$) was compared with T followed by MPA at relapse ($n = 39$) in patients with previously untreated advanced breast cancer (study 1). Cyclical T/MPA ($n = 21$) was compared with MPA ($n = 20$) in previously treated patients (study 2). Measurements of serum T and MPA confirmed cyclical drug levels. In both studies the response rate and median durations of response to T/MPA were not significantly different to T (study 1) or MPA (study 2) alone. In study 1 the median survival for T/MPA was 20 months and for T followed by MPA was 35 months ($P$ = NS). In study 2 the median survival figures were 13.5 and 15 months for cyclical and sequential treatment respectively. We conclude that T is a partial agonist with respect to PR *in vivo* and that, although tumours respond to T/MPA the cyclical regimen is not superior to sequential treatment with T followed by MPA at relapse.

**High dose *versus* low dose oral medroxyprogesterone acetate (MPA) in advanced breast cancer: A randomised trial**

C.J. Gallagher, J.A. McKinna, F. Cairnduff & I.E. Smith

*Breast Unit, Royal Marsden Hospital, London UK.*

The majority of studies claiming benefit for high dose MPA in advanced breast cancer are non-randomised. Dose response for oral MPA was therefore investigated in 124 patients (pts) with advanced breast cancer randomised to low dose (LD) 300 mg day$^{-1}$ (66 pts) or high dose (HD) 1000 mg day$^{-1}$ (58 pts) MPA. The groups were evenly matched for age, menopausal status, disease-free interval, sites of disease, and previous treatment. Thirty-three pts had received no previous endocrine therapy. Overall response (UICC criteria) was 24% for both LD (16/66) and HD (14/58) pts, with stable disease in a further 12 (18%) LD and 17 (29%) HD pts. Median response duration was 11 months (mo) (range 2–19 mo) for LD *vs.* 10 mo (range 3.5–25 mo) for HD; median survival (all pts) was 13 mo (LD) *vs.* 11 mo (HD). Response by site was similar for LD and HD; relief of bone pain occurred in 43% LD and 52% HD pts, although objective resclerosis occurred in only 13% for both LD and HD. For previously untreated pts, 6/19 (32%) responded to LD and 4/14 (29%) to HD MPA. For previously treated pts, 10/47 (21%) responded to LD and 11/44 (25%) to HD MPA. Two of 4 premenopausal pts responded to LD and 3/6 to HD (total 50% response). Both treatments were in general well tolerated, but toxicity was greater for HD than LD including in particular fluid retention 26% *vs.* 6%) and weight gain (10% *vs.* 2%); and for HD treatment had to be altered for 18% of pts (dose reduction 9%; treatment stopped 9%) compared with treatment stopped for only 2% of LD pts ($P < 0.01$). LD is as effective as HD oral MPA for advanced breast cancer, and is cheaper and less toxic.

**A randomised trial of short course (9 weeks) mitoxantrone *versus* continuous therapy in advanced breast cancer**

A.L. Harris, B.M.J. Cantwell, S. Ghani, P. Dawes, R.G.B. Evans, H. Lucraft, R. Wilson & J. Farndon

*University Departments of Clinical Oncology, Surgery and Radiotherapy, University of Newcastle-upon-Tyne, Newcastle-upon-Tyne, UK.*

Mitoxantrone (M) 14 mg m$^{-2}$ every 3 weeks was used as first-line chemotherapy for 80 patients with advanced breast cancer. After 4 courses (9 weeks), responders were randomised to continue (C) until relapse or stop (S). Patients on the short course (S) were re-treated with M on relapse. On relapse, C and the non-responders were treated with adriamycin (A) 40 mg m$^{-2}$. Seventy-one patients had previous hormone therapy (median 2 types, range 1–4) and 23% had responded. Sixty-four patients had prior radiotherapy. Ages ranged from 28–78 years, median 57. Years from LMP ranged from 0–40 years (median 6). Tumour-free interval ranged from 0–181 months (median 11 months). Thirty-three patients were performance status 2, 45 PS 1, 2 PS 0.

The response to M was 26% partial response, 8% stable disease for $> 3$ months. Twelve patients were randomised to (C) and 13 to (S). There was no difference in age, years from LMP, tumour-free interval, weight, time from first recurrence to start of M or performance status in the 2 arms. The median response duration was 7 *vs.* 8 months (S *vs.* C), the median survival from start of treatment was 10 *vs.* 9 months (S *vs.* C) and survival from first recurrence 31 *vs.* 29 months (S *vs.* C). Toxicity was mild. This study shows short course therapy is as effective as continuous therapy and therefore chemotherapy should be stopped once response has occurred. Drug resistance must emerge within the first 9 weeks or be present before treatment starts.

**The prognostic significance of dual parameter flow cytometric analysis of primary and metastatic breast cancer**

J. Lawry[1] & K. Rogers[2]

[1]*Department of Virology and* [2]*Department of Surgery, The University of Sheffield Medical School, Beech Hill Road, Sheffield S10 2RX, UK.*

The use of dual parameter correlated (DPC) flow cytometry enables the simultaneous analysis of DNA cell cycle and monoclonal antibody (MoAB) binding on the same sample, providing valuable information on tumour heterogeneity. Using tissue and cell specific markers to cytokeratin, human milk fat globulin and infiltrating T-cells, in conjunction with the DNA fluorochrome propidium iodide, samples can be subdivided into cells which are MoAB positive or negative, diploid or aneuploid, and proliferating or non-proliferating simultaneously.

Results of these multiparameter analyses for 28 primary breast tumours, 15 lymph node metastases, and 4 samples of normal breast tissue taken at mastectomy, correlated to clinical stage and histological grade, infer a role for DPC flow cytometric analysis in assessing disease status, not achievable by single parameter DNA analysis. Furthermore, due to the ability to sub-classify a heterogeneous tumour sample into several populations of cells, the identification of possible sites of metastatic spread may be made more accurate, and clinically relevant.

### Phase I

### Preclinical screening of new anticancer agents using panels of human continuous cell lines

W.E.A. Jenkins[1], R.H. Shoemaker[2] & J.R.W. Masters[1]

[1]*Department of Histopathology, Institute of Urology, St Paul's Hospital, 24 Endell Street, London WC2H 9AE,* [2]*Division of Cancer Treatment, National Cancer Institute, Bethesda, Maryland 20892, USA.*

The use of continuous cell lines as a model for the *in vitro* pre-clinical screening of new anticancer agents was investigated. We compared the *in vitro* sensitivities to 12 novel agents (obtained from the NCI) of 5 lines derived from transitional cell carcinomas of the human bladder (RT4, RT112, T24, HT1376, HT1197), 4 from non-seminomatous testicular germ cell tumours (SuSa, 833K, 1618K, GH) and 2 from normal human urothelium (HU609, HS0767). Cytotoxicity was assessed by inhibition of colony forming ability during continuous exposure to a range of drug concentrations. Parallel assessments are being made of these agents *in vitro* using the human tumour stem cell assay (HTCSA) and *in vivo* using the transplantable murine P388 tumour and 2 human tumour xenografts, and where appropriate in clinical trial. In this study, 3 agents displayed no cytotoxicity up to a maximum concentration of $40\,\mu g\,ml^{-1}$. Seven drugs were active in the concentration range 1–8 $\mu g\,ml^{-1}$, one between 0.3 and $2.0\,\mu g\,ml^{-1}$ and the most cytotoxic agent inhibited colony development in the range 30–100 $ng\,ml^{-1}$. In keeping with clinical experience, the testicular tumour cells were generally more sensitive than the bladder cancer cell lines, with the exception of one drug to which the pattern was reversed. The 'normal' cell lines were, on average, more sensitive to the agents than the bladder cancer cell lines, and thus this comparison failed to demonstrate differential cytotoxicity to tumour cells. In terms of relative cytotoxicities, the data obtained using the cell lines and the HTCSA were similar, and for this purpose cell lines are more reproducible and cost-effective.

### A novel strategy for dose escalation in phase I clinical trials of chemotherapeutic agents

D.B. Smith[1] & C. Ewen[2]

[1]*Department of Medical Oncology and* [2]*Paterson Institute for Cancer Research, Christie Hospital and Holt Radium Institute, Manchester M20 9BX, UK.*

Dose escalation in Phase I clinical trials must strike a balance between the risk of excessive toxicity due to rapid increases and treating many patients at ineffective doses through over-conservative increments. The following data were obtained from a review of 61 Phase I trials published since 1970:

Escalation: Fibonacci 41 (67%) steps to 1st toxicity median, range 3 (0–7).
50–100% 12 (20%) steps to MTD median, range 7 (0–15).

Starting dose: mouse 23; dog 23; monkey 2; unspecified 13. Forty-three per cent of patients overall received less than the dose recommended for Phase II, and toxic deaths occurred in 12 trials (20%). In only 5 trials was the MTD $<5\times$ the starting dose ($5n$) and in each case toxicity was seen at the starting dose. Thus in all 61 trials it would have been safe to escalate to $5n$ if the starting dose was found to be non-toxic. Subsequent escalations may be guided by pharmacokinetic data. Collins *et al.* (*Cancer Treat. Rep.*, **70,** 73, 1986) have shown a close correlation between the AUC at the mouse LD10 and the AUC in the MTD in man. Comparison of the AUC at $5n$ with mouse data will allow rational selection of the next escalation step. This should not normally exceed 100% since the AUC may rise more rapidly than expected in drugs exhibiting non-linear kinetics. A Phase I trial of the new spindle poison ICI 134,154 has been carried out using this scheme. The AUC at the mouse LD10 was $313\,\mu g\,l^{-1}\,h^{-1}$. The starting dose was $40\,mg\,m^{-2}$ (1/10 LD10 mouse). The AUC at $200\,mg\,m^{-2}$ was $11\,\mu g\,l^{-1}\,h^{-1}$ and at $400\,mg\,m^{-2}$ was $39\,\mu g\,l^{-1}\,h^{-1}$. Two patients have received $800\,mg\,m^{-2}$. In one the AUC was $116\,\mu g\,l^{-1}\,h^{-1}$ and in the other $228\,\mu g\,l^{-1}\,h^{-1}$. The latter patient developed alopecia and a WBC nadir of $1.4\times10^{9}\,l^{-1}$. This, therefore, appears to be close to the MTD. Using the methods outlined the numbers of patients treated at ineffective doses and the duration of Phase I trials may be reduced by up to 50%.

### A Phase I study of meta-azidopyrimethamine ethane sulphonate (MZPES) – a new dihydrofolate reductase inhibitor

N.S.A. Stuart[1], M. Crawford[2], G.R.P. Blackledge[1], E.S. Newlands[2], J. Slack[3], R. Hoffman[2], J. Kavanagh[1], M. MacKenzie[1] & D.S. Northcott[2]

[1]*Queen Elizabeth Hospital, Birmingham,* [2]*Charing Cross Hospital, London and* [3]*University of Aston, Birmingham, UK.*

MZPES is a new dihydrofolate reductase (DHFR) inhibitor of the diaminopyrimethamine group first synthesised in 1978. It has the potential advantage of methotrexate, the standard DHFR inhibitor, of being lipophilic thus being able to cross the blood–brain barrier and also possibly overcome transport-mediated drug resistance by passive diffusion. It also has a relatively short half-life compared to other similar compounds raising the possibility of reduced toxicity to normal tissues. Animal studies have shown activity against L1210 leukaemia (++), B16 melanoma (++), M5076 reticulum cell sarcoma (++), P388 leukaemia (+) and TLX5 lymphoma (+).

MZP is undergoing Phase I assessment in advanced malignancies unresponsive to standard treatment being administered in 5% dextrose as a 1 h i.v. infusion. Starting dose was 10% of the LD10 in mice, i.e. $5.4\,mg\,m^{-2}$ with dose increments to 11, 18, 27, 38, 50, 67, 83, 105, 125, 150, 180, 210, 250, 300, 360 and $400\,mg\,m^{-2}$. Sixty-two patients have received 128 courses. Toxicity has comprised (a) nausea and vomiting (WHO grade 3/4) at doses of $180\,mg\,m^{-2}$ or more; (b) light-headedness, dizziness, incoordination and malaise at doses of $300\,mg\,m^{-2}$ or more; (c) Leucopenia (WBC $<3.5\times10^{9}\,l^{-1}$) in 4/35 courses and thrombocytopenia (platelets $<150\times10^{9}\,l^{-1}$) in 4/35 courses above $210\,mg\,m^{-2}$. One patient who had brain metastases developed generalised convulsions immediately following $210\,mg\,m^{-2}$. EEG recordings during and after infusion in subsequent patients receiving 210 and $250\,mg\,m^{-2}$ showed no abnormalities. One patient suddenly became unconscious 20 h after $400\,mg\,m^{-2}$. He had clinical signs of an intra-cranial bleed, remained comatose and died. Post-mortem was refused. Pharmacokinetics studies at all doses except $5.4\,mg\,m^{-2}$ indicate the drug obeys 2-compartment kinetics with an elimination half-life of 37 h. No responses have been seen.

MZPES is a novel DHFR inhibitor with interesting chemical properties and good anti-tumour activity in murine tumour models. This study suggests that maximum tolerated dose when administered by 1 h i.v. infusion will be in the range 400–500 $mg\,m^{-2}$ with dose limiting toxicity being neurological symptoms and nausea and vomiting. Further study is required to determine whether this drug has anti-tumour activity in man.

**Phase I clinical and pharmacokinetic study of flavone acetic acid**

C. Bradley[1], S.B. Kaye[1], D.J. Kerr[1], A. Setanoians[1], J. Cassidy[1], L. Adams[1], E.M. Rankin[1], T. Young[2], G. Forrest[2] & M. Soukop[2]

*Departments of Medical Oncology, [1]Glasgow University and [2]Glasgow Royal Infirmary, UK.*

Flavone acetic acid, a synthetic flavonoid aglycone, is active in a range of murine tumours refractory to conventional cytotoxic agents. A therapeutic plasma window of 100–500 $\mu g\,ml^{-1}$ was described in mice and it was noted that cytotoxic efficacy was related to the duration of drug exposure above the therapeutic threshold. Forty-three patients (median age, 55 years, median performance status, 1) with predominantly metastatic colorectal carcinoma were entered in the Phase I trial. Three patients were entered at each dose level and escalation was by a modified Fibonacci regime. Drug was administered weekly ($\times 3$) with bicarbonate (1.26%) by a 1 h infusion initially and subsequently 3 and 6 h infusions. Dose limiting toxicity for 1 h infusion (at 6.4 $g\,m^{-2}$) consisted of intolerable flushing and a sensation of warmth. The peak drug level obtained (with 1 h infusion) was 1,225 $\mu g\,ml^{-1}$. The drug demonstrated non-linear pharmacokinetics over the dose range of 500 mg to 6.4 $g\,m^{-2}$. Further dose increments using a 6 h infusion were performed and the current entry dose is 10 $g\,m^{-2}$. Dose limiting toxicity using a 6 h infusion has not yet been reached, although one episode of hypotension has been noted. The peak drug level with a 6 h infusion (at 8.6 $g\,m^{-2}$) is 650 $\mu g\,ml^{-1}$, but calculations of AUC ($>100\,\mu g\,ml^{-1}$) confirm that greater drug exposure is obtained using longer infusion. No responses have yet been seen, but since murine therapeutic levels are clinically achievable, Phase II trials are planned through the Cancer Research Campaign Phase II Committee using the 6 h infusion schedule.

**Site dependant sensitivity of a transplantable mouse colon tumour to flavone acetic acid (LM975)**

J.A. Double, M.C. Bibby & R.M. Phillips

*Clinical Oncology Unit, University of Bradford, Bradford, West Yorkshire BD7 1DP, UK.*

Previous studies with flavone acetic acid, LM975, (Bibby *et al., Br. J. Cancer,* in press, 1987) have demonstrated spectacular responses of established s.c. solid transplantable colon tumours in mice (MAC tumours). The plasma profiles of LM975 associated with this activity have now been attained in man (Kerr *et al.*, 1986 Proceedings of the Fifth NCI/EORTC Symposium on New Drugs in Cancer Therapy, Amsterdam) but no responses have yet been seen. The present study attempts to explain this by examining the chemosensitivity to LM975 of transplantable adenocarcinomas of the mouse colon grown in different sites. MAC 15A is an ascitic variant of MAC 15 and i.v. administration of the ascites cells produces systemic disease. Responses to chemotherapy are measured by a survival assay and are essentially the same for standard agents as those seen with the solid and ascitic variants. The best responses to date are seen with the nitrosoureas. Subcutaneous inoculation of ascites tumour cells produces a poorly differentiated tumour which is sensitive to intraperitoneal treatment with LM975. Neither the ascites tumour nor the systemic disease responds to LM975. The lack of response of the ascites tumour may be explained by rapid peritoneal clearance whereas it is difficult to envisage a pharmacokinetic explanation for the insensitivity of the systemic model. Preliminary *in vitro* studies with MAC 15A suggest that plateau phase cells are 4 times more sensitive than log phase cells to LM975 indicating that cell kinetics may be an important factor in determining response. These results have demonstrated the importance of tumour site in determining chemosensitivity to LM975 and this fact should be taken into account in the assessment of its clinical activity.

**Continuous infusion chemotherapy with ifosfamide (IFX) – pharmacokinetics, toxicity, efficacy**

L.D. Lewis, J. Warin[2] & C.G. Rowland[2]

*[1]Department of Clinical Pharmacology, United Medical and Dental Schools, Guy's Hospital, London SE1 9RT and [2]Department of Radiotherapy, Royal Devon and Exeter Hospital, Exeter, UK.*

IFX is an alkylating agent which has proved effective in the treatment of a number of neoplastic disorders. Certain chemotherapeutic agents e.g. vinblastine and bleomycin (Samuels *et al., Cancer Chem. Rep.,* **59,** 563, 1978), have proven more effective without increased toxicity when given by long term i.v. infusions.

We studied 6 patients (3 male), median age 64.5 (range 55–72) yrs, median Karnofsky score 90 (range 90–100), 4 had inoperable bronchial ca., 1 adeno ca. ovary, 1 transitional ca. bladder. They received home based treatment (3 cycles) of IFX 1 g and mesna 800 mg infused i.v. (by a battery driven syringe pump attached to a CVP line) every 24 h for 10 days at monthly intervals.

We performed pharmacokinetic studies on 8 courses (2 patients were studied on 2 occasions) prior to therapy and at timed intervals at the end of the infusion blood samples were taken. Plasma IFX concentrations and alkylating activity were measured using published GLC and spectrophotometric methods respectively.

Pharmacokinetic results ($n=8$) median (range): plasma IFX elimination half-life = 6.0 (3.9–8.7) h; AUC 1,907.7 (1,056.2–2,2929.1) $\mu g\,ml^{-1}$; Css IFX 7.95 (4.4–12.2) $\mu g\,ml^{-1}$; IFX clearance = 87.5 (56.9–157.8) $ml\,min^{-1}$; Vd = 619.9 (471.1–996.6) $ml\,kg^{-1}$. No plasma alkylating activity was detected. In several of the patients analysing intermittently obtained urine samples alkylating activity was qualitatively detected.

Three of 6 patients had slight nausea, 4/6 mild alopecia, 1/6 had proteinuria, no haematological toxicity or haematuria was noted. Three of 6 had a partial response.

This treatment regime produces no detectable plasma IFX metabolites, very good tolerance and a good tumour response.

**Pharmacokinetics of intravenous and oral mesna**

C.A. James, T.G.K. Mant, H.J. Rogers & P.G. Harper

*Department of Clinical Pharmacology, United Medical and Dental Schools, Guy's Hospital, London SE1 9RT, UK.*

Mesna (sodium 2-mercaptoethane sulphonate) prevents the urotoxic side effects of the oxazaphosphorines. We have investigated its pharmacokinetics in 6 healthy volunteers (3 male) who received 800 mg of mesna by i.v. and oral routes.

Mesna and dimesna (the sole metabolite) were measured in plasma and urine by reverse-phase ion-pair HPLC followed by electrochemical detection (James & Rogers, *J. Chromatogr.*, **382,** 394, 1986).

Following i.v. injection plasma mesna concentrations were fitted by a one compartment model with mean + s.d. $t_{1/2}$ $22.0 \pm 3.1$ min. The terminal $t_{1/2}$ for dimesna was $1.17 \pm 0.32$ h. Following oral mesna the lag time before appearance of mesna varied from 0.33h to 2.5h and for dimesna from 0.25 to 0.5h with late peaks of mesna and dimesna concentrations (little free mesna was detected in the plasma of 2 subjects).

After i.v. administration most of the urinary mesna excretion occurred in the first 4h. After oral dosing mesna excretion was more prolonged over 8h, although generally more was excreted in the first 4h. A mean of $31.8 \pm 11\%$ of the dose was excreted as mesna after the i.v. dose and $17.6 \pm 9.2\%$ after p.o. Total urinary excretion (mesna+dimesna) over 24h was $65.1 \pm 11.4\%$ after i.v. and $49.3 \pm 13.7\%$ following p.o. dosing.

The urinary bioavailability of free mesna was $53.0 \pm 19.5\%$. The formal oral systemic bioavailability based on the renal excretions of mesna and dimesna was $76.1 \pm 16.1\%$.

These results suggest that oral doses of mesna should be double those used for i.v. and that either the first oral dose should be given 2h before the oxazaphosphorine, or an initial i.v. dose should be given with the oral dose.

### Mitozolamide: Phase II studies in melanoma, lung and ovarian cancer

M. Harding[1], S.B. Kaye[1], A. Dorward[3], R. Mackie[2], J. Smythe[4] & G. Blackledge[5]

*Departments of [1]Medical Oncology and [2]Dermatology, University of Glasgow, [3]Royal Alexandra Infirmary, Paisley, [4]Department of Medical Oncology, University of Edinburgh and [5]Department of Medicine, University of Birmingham, UK.*

Mitozolamide, a novel cytotoxic agent structurally similar to the chlorethyl-nitrosoureas, has shown significant preclinical activity in murine tumours and human tumour xenografts. The initial dose in these Phase II trials was $115\,mg\,m^{-2}$ orally, with retreatment planned for 6 weekly intervals, in 71 patients. Severe thrombocytopenia (median nadir $29 \times 10^9\,l^{-1}$) occurred in the first 11 lung cancer patients. The dose was reduced to $90\,mg\,m^{-2}$ or $70\,mg\,m^{-2}$ for heavily pretreated patients. Gastrointestinal toxicity was mild (grade 0–2). Unpredictable myelosuppression occurred despite dose reduction: median nadir values $2.6–4.8 \times 10^9\,l^{-1}$ WBC; $60–131 \times 10^9\,l^{-1}$ platelets, but Grade IV thrombocytopenia was still seen. This contributed to 5 patient deaths within 7 weeks of treatment. Only 31 patients received 2 or more cycles at 6–8 week intervals because of delayed myelosuppression (nadir WBC 5–6 weeks).

| *Tumour* | *Number* | *Ineval/ too early* | *PR* | *SD* | *PD* |
|---|---|---|---|---|---|
| Lung (non small cell) | 15 | 3 | – | 4 | 8 |
| Lung (small cell) | 20 | 2 | 5 | 2 | 11 |
| Melanoma | 16 | – | 2 | 2 | 12 |
| Ovary | 20 | 6 | – | 3 | 11 |

These preliminary data indicate activity in small cell lung cancer (3 PR in 11 pretreated, 2 PR in 9 previously untreated patients) but response duration was short (6–26 weeks). Activity in melanoma requires confirmation, and resistance of ovarian cancer may reflect extensive prior therapy. Clinical application of mitozolamide, particularly in combinations, would be difficult because of unpredictable and frequently cumulative myelosuppression. The ovarian cancer study was performed by the Cancer Research Campaign Phase II Committee.

### Phase I study of recombinant human tumour necrosis factor

P. Selby, K. Fearon, H. Calvert, S. Hobbs, C. Viner, J. Hall & T.J. McElwain

*Sections of Medicine, Nursing and Biomedical Pharmacology, Institute of Cancer Research, Sutton and Department of Medical Oncology, Glasgow Royal Infirmary, UK.*

Tumour Necrosis Factor is a protein produced by mammals after challenge with bacterial endotoxins. It is cytotoxic *in vitro* for human tumour cell lines and produced necrosis and regression of experimental murine tumours. It is also a potent inhibitor of lipoprotein lipase synthesis *in vitro* (cachectin). Large quantities of pure protein are available as a result of expression of the gene in *E. coli*.

A Phase I study of human recombinant TNF is reported here. The drug was given by a 1h infusion repeated every 2 weekly period for 3 doses with one dose escalation for the third treatment. Three patients at each dose level. Treatment began at a dose of 1/1,000th of the murine LD10 equivalent ($9 \times 10^3\,U\,m^{-2}$) and was escalated to $1.2 \times 10^6\,U\,m^{-2}$. Eighteen patients with malignancies unresponsive to conventional therapy were treated.

All patients experienced fever with rigors but this reaction was significantly reduced by pretreatments with steroids and indomethacin. At doses of $6–12 \times 10^5\,U\,m^{-2}$ patients developed abnormal liver function tests, transient hypotension, transient leucopenia and changes in serum creatinine. The maximum safe dose appears to be $\sim 1 \times 10^6\,U\,m^{-2}$. Lipid tolerance tests revealed no inhibition of triglyceride clearance. Pharmacokinetic studies showed rapid clearance ($t_{1/2}$ $\sim$20min). Two patients, both with lymphoma, showed evidence of response to therapy.

### Ovarian and gonadal tumours

### Randomised trial comparing abdomino-pelvic radiotherapy with *cis*-platinum in patients with ovarian cancer with no macroscopic disease after primary surgery

C.W.E. Redman, F.D. Lawton, D. Luesley, C. Hilton, J. Mould, T. Latief, K.K. Chan & G. Blackledge

*CRC Clinical Trials Unit, Queen Elizabeth Hospital, Edgbaston, Birmingham, UK.*

The objective of this study was to compare radiotherapy, as described by Dembo *et al.* (*J. Rad. Oncol. Biol. Phys.*, **5**, 1933, 1978) with single agent *cis*-platinum in the management of patients with no macroscopic disease after primary surgery. Between October 1981 and October 1986 37 patients were randomised to receive either pelvic plus abdomino-pelvic irradiation, using the moving strip technique (2250cGy midplane; total dose to pelvis 4,500cGy) or 5 courses of single agent *cis*-platinum ($100\,mg\,m^{-2}$ i.v. every 21 days). Data on the first 28 patients is available for analysis. Fourteen patients were randomised into each treatment group. The two groups were comparable for age, tumour histology and differentiation, and FIGO staging (Ic to III). Three of 14 patients, all in the chemotherapy arm, did not receive full protocol therapy (2 patients received only 4 courses because of toxicity; 1 patient received 5 courses but at reduced dosage).

The study group has had a median follow-up of 37 months and 71% are alive at 48 months. There have been 4 disease-related deaths and 1 unrelated death in the radiotherapy group; there was progressive disease noted at the end treatment in 3/4 of the disease related deaths. There have been 3 deaths in the chemotherapy group. There is no significant difference in survival between the two groups (log rank chi square (1 df) = 0.900; $P = 0.34$). Two patients, one from each group, have evidence of recurrent disease. Three patients in the radiotherapy arm have experienced radiation induced enteritis requiring surgical management. These preliminary results attest to the favourable prognosis of patients with no macroscopic disease following primary surgery. At this stage, there is no significant survival difference between the two groups, although there is long term morbidity from significant radiation induced enteritis. Further follow-up is required to determine whether *cis*-platinum will continue to give results comparable with those achieved by radiotherapy in the study of Dembo *et al.*

### GR38032F, a selective 5-HT3 receptor antagonist, is anti-emetic in patients receiving cytotoxic drugs

D. Cunningham[1], J. Hawthorn[2], A. Pople[3], J.-C. Gazet[4], H.T. Ford[4], T. Challoner[5] & R.C. Coombes[1,3]

[1]*Medical Oncology Unit,* [2]*Department of Physiology,* [3]*Ludwig Institute for Cancer Research (London – St George's Group), St George's Hospital Medical School, London SW17 0RE,* [4]*St George's Hospital, London SW17 0QT and* [5]*Glaxo Group Research Ltd, Ware, Herts, UK.*

GR38032F (Glaxo Group Research, Ware) is a novel highly selective 5HT3 antagonist which has been shown to be capable of abolishing *cis*-platin induced emesis in the ferret. This is the first report of the use of this class of compound to treat emesis in patients receiving chemotherapy.

We have treated 15 patients receiving a variety of cytotoxic drug combinations (excluding *cis*-platin) which had produced nausea and vomiting refractory to first-line anti-emetics. GR38032F, 4 mg i.v. and 4 mg p.o., was given immediately prior to chemotherapy, the oral dose was then repeated 5 and 10 h later. Nausea, vomiting and side effects were recorded for the following 24 h. The 15 patients received a total of 31 courses of chemotherapy. During this time only 1 patient experienced vomiting. The only noticeable side effects were dryness of the mouth in one patient and mild sedation in one other, which were not clearly drug related.

Results from this open study indicate that GR38032F successfully controls refractory vomiting caused by cytotoxic drugs with negligible side effects.

### Carboplatin and cyclophosphamide chemotherapy in advanced ovarian cancer

M. Teeling & D.N. Carney

*Department of Oncology, Mater Hospital, Dublin 7, Eire*

A Phase II study of carboplatin (300 mg m$^{-2}$ i.v.) and cyclophosphamide (600 mg m$^{-2}$ i.v.) was carried out in 26 patients (pts) with newly diagnosed advanced stage ovarian cancer. Courses were administered at 4-weekly intervals for a total of 6 cycles. Therapy was administered as an out-patient without prehydration or forced diuresis. Antiemetic therapy consisted of oral high dose dexamethasone (24 mg d$^{-1}$) and metoclopramide (60 mg d$^{-1}$) commencing 12 h prior to cytotoxic therapy. Patients were staged with CT scans before, during and after 6 cycles of chemotherapy (CT). To date 26 pts with FIGO stage II (2 pts with residual disease), III & IV have received 105 cycles of chemotherapy. Treatment was well tolerated by most patients with minimal nausea and vomiting (WHO grade 1–2) observed during 35% of the cycles. Two patients developed moderate alopecia. No clinical or biochemical evidence of nephrotoxicity was seen. Tinnitus, deafness or peripheral neuropathy have not been observed to date. Myelosuppression was acceptable with mean nadir WCC of 4.3 (range 1.4–9.0) and mean nadir platelet count of 273 (range 39–536) observed on day 21. There were no therapy related infective episodes. Twenty pts are evaluable for response including 7 pts with residual post-operative bulky tumour. The response rate (CR = 13 + PR = 2) was 75%. Six of the 7 patients who failed to achieve CR with treatment had residual bulky tumour following initial surgery. Although preliminary, these results suggest (1) carboplatin and cyclophosphamide in the above dose is an active out-patient regimen that is well tolerated in pts with newly diagnosed ovarian cancer, (2) toxicities, including haematological renal and auditory are minimal.

### Phase II studies of mitozantrone and platinum in advanced epithelial ovarian cancer (EOC); an active regimen with minimal toxicity by intravenous or intraperitoneal route

F.F. Lawton[1], C. Redman[2], D. Luesley[1], J.J. Mould[3], D. Spooner[3], A.D. Chetiyawardana[3] & G. Blackledge[2]

*Departments of* [1]*Obstetrics and Gynaecology,* [2]*Medicine and* [3]*Radiotherapy, Birmingham, UK.*

Mitozantrone (M) can achieve 25% responses in patients with EOC previously treated with platinum. As a result of this activity and relative lack of toxicity, it has been incorporated into a first-line regimen with *cis*-platinum (D). Twenty-three patients (pts), 18 previously untreated, median age 61 years, all with FIGO stage III or IV disease received D 75–100 mg m$^{-2}$ and M 12–14 mg m$^{-2}$ i.v. q. 3 weeks for 6 cycles. Twenty pts had evaluable disease and 16 (80%) showed at least a partial response (WHO criteria). Median duration of remission has not yet been reached (median follow-up 11 months). Subjective toxicity was minimal. Sixteen pts had greater than WHO grade I emesis and 4 pts had greater than grade II alopecia. Eleven pts had greater than WHO grade I myelotoxicity and 11 pts required transfusion. Three cycles of i.v.-MD could be given with minimal myelosuppression but cumulative marrow suppression produced treatment delay by 1 to 4 weeks in 12 pts (4 at cycle 3, 5 at cycle 4, 3 at cycle 5) and in 8 pts treatment was discontinued after 5 cycles i.v.-MD because of myelosuppression. Twelve patients with sub-optimal primary surgery, were scheduled to undergo secondary surgical debulking (IDS) after 2–3 courses of chemotherapy. Eight of 12 had optimal resection; 1 remained inoperable, 1 had had insufficient response to MD to consider further surgery; 1 had medical contraindications, and there was 1 early disease related death. These data suggest that the majority of patients with EOC can be rendered optimally debulked by MD and early secondary surgery. Intraperitoneal therapy is now being used in these patients, (1) to optimise drug concentration at the site of possible residual tumour and (2) to minimise subsequent systemic cumulative toxicity.

Treatment plan for pts with sub-optimal primary surgery now consists of: primary laparotomy; i.v.-MD × 3; second surgery with insertion of indwelling i.p. catheter; i.p.-MD × 5 q. 3 wks with the following dose escalation: D 100 to 200 mg m$^{-2}$, M 15 to 25 mg m$^{-2}$ with i.v. sodium thiosulphate infusion during the i.p. cycles. Preliminary results suggest that these doses can be safely administered with little myelosuppression and minimal reduction in renal function.

Combination i.v.-MD is an active regimen in EOC with dose limiting myelosuppression. Over 75% of pts can be rendered to less than 2 cm residual disease with a combination of primary laparotomy, i.v.-MD × 3 and IDS and would therefore be candidates for i.p.-MD. Such combined approaches need further evaluation in EOC.

### Antigen expression in human ovarian adenocarcinoma cell lines

S.P. Langdon[1], F.G. Hay[2], M.M. Hawkes[1], S.S. Lawrie[1], R.C.F. Leonard[2], J.F. Smyth[1,2], D. Schol[3] & J. Hilgers[3]

[1]*Imperial Cancer Research Fund Medical Oncology Unit,* [2]*University Department of Clinical Oncology, Western General Hospital, Edinburgh, UK and* [3]*Netherlands Cancer Research Institute, Amsterdam, The Netherlands.*

The immunological characterisation of 9 ovarian carcinoma cell lines was undertaken to determine if antigen expression might correlate with differentiation status in this disease. Six lines derived from poorly differentiated adenocarcinomas (PD) were compared with 3 lines derived from a patient with a well differentiated serous adenocarcinoma (WD).

Cells were air dried onto multispot slides and acetone/methanol fixed. Mouse anti-human monoclonal antibodies (MoAB) were applied followed by immunoperoxidase staining (PAP staining).

In the PD group, expression of the neuroendocrine antigen 123C3 and vimentin was low (<5% and <14% cells +ve respectively) but increased in the WD lines (>19% and >51% cells +ve). Conversely expression of OC125 was high in the PD lines (>51% cells +ve) and reduced in the WD lines (<33% cells +ve). PE016 (a PD cell line) was exceptional and possessed properties similar to the WD lines. The MoAbs AUA1 (anti-epithelial) and LE61 and CAM5.2 (anti-cytokeratin) reacted with almost all cells in all cell lines (except for PE016) as did anti-CEA. HMFG1 and HMFG2 were present in all cell lines (% varying between 1% and 87%).

MHC class I expression (MoAb 92.1) was present in 2/9 cell lines and Class II expression (MoAb CR343) in 6/9 lines. Anti-placental alkaline phosphatase MoAbs (H17E2, 3F6, 11F7 and 8B6) reacted with 6/9 cell lines (1–9% cells +ve).

Therefore expression of 123C3 and vimentin appear to correlate directly, and OC125 inversely, with serous differentiation in these cell lines.

### The clinical role of Ca 125 in ovarian cancer

J. Cassidy[1], M. Kerr[1], J. Roulston[2] & R.C.F. Leonard[1]

[1]*University Department of Clinical Oncology, Western General Hospital, Edinburgh and* [2]*University Department of Clinical Chemistry, Royal Infirmary of Edinburgh, UK.*

Two hundred and thirty-six serum samples from 61 patients with histologically proven ovarian carcinoma were analysed for Ca 125 level using a commercial IRMA assay (Centocor Inc., Malvern, Pennsylvania, USA). The results of these assays were correlated with clinical and radiological (including ultrasound) assessments of disease status during and after chemotherapy. Twenty-nine patients in the study had a second-look laparotomy; in 21 patients pre-operative levels of Ca 125 are available. The upper limit of normal Ca 125 was taken as 35 U ml$^{-1}$. In patients who did not have second-look surgery, a true negative result was defined as a period of 6 months off therapy without evidence of recurrence. Overall our results give 60.5% sensitivity, 91.3% specificity and 66.5% accuracy. The levels of Ca 125 showed a correlation with disease progression or response in 64.7%. Of the 21 evaluable patients who had a second-look laparotomy; no patient had a positive Ca 125 result with a subsequent negative laparotomy. Second-look surgery could, therefore, have been avoided in 6/21 cases (28.6%) on the basis of elevated Ca 125. In 21 patients assays were inconsistent over time due to false negatives. In 11 the fluctuations were wide, but in 10 could be explained by the inherent imprecision of the assay at antigen concentrations of <100 U ml$^{-1}$. It is our conclusion that serum Ca 125 assay is a useful marker in ovarian carcinoma, and may be of value in the avoidance of unnecessary second-look surgery.

### An assessment of urinary polyamine excretion in the management of patients with epithelial ovarian cancer

F.G. Lawton[1], M. Griffin[2], J.A. Slack[2] & G. Blackledge[3]

[1]*Departments of Obstetrics and Gynaecology and* [3]*Medicine, University of Birmingham and* [2]*Institute of Pharmaceutical Sciences, University of Aston, UK.*

Urinary polyamine (UPA) excretion patterns have been shown to correlate with a number of clinical parameters including disease bulk, remission status, clinical course of the disease and prediction of relapse in a variety of malignancies, but little data are available relating their role in the management of patients with epithelial ovarian cancer.

The polyamines (PA) (putrescine, spermidine, spermine (put, spd, spm)), their acetylated derivatives (ACPA) (acput, acspd, acspm) and total UPA (totput, totspd, totspm, totupa) levels were measured in random urine samples by HPLC (Spragg & Hutching, *J. Chromatogr.*, **258,** 289, 1983) in 43 control subjects (group A), 15 patients (pts) with benign pelvic masses (B), 11 pts with FIGO stage I disease (C) and 57 pts with advanced EOC (D).

Depending on the UPA specified, between 1 and 7 (median 2) of 58 normal pts (A and B combined) had levels above the upper limit of normal (false positive rate 2 to 13%) while true positive rates varied from 11 to 30%. Overall 46% of pts with EOC had at least one raised UPA level.

There were significant differences in pre-laparotomy levels of ACPA in patients with benign and malignant pelvic masses.

A single UPA was raised in only 1 of 5 pts with well differentiated EOC compared with 4 of 10 pts with moderate differentiation and 12 of 15 with poorly differentiated tumours.

Comparison of paired UPA levels over a 5 to 6 month period showed significant increases in put, acput and totput for pts with progressive disease and significant falls for pts with responding disease.

Chemotherapy (CT) produced rises in UPA excretion in 49% (for totspd) to 79% (for put) of pts within 48 hours of treatment.

The ratio UPA 48 hrs post-CT/UPA pre-CT was significantly higher for pts who would eventually respond to CT compared with those who would not for spd, acput, acspd, totput, totspd and totupa.

UPA excretion can aid the differential diagnosis of pelvic masses, reflects tumour bulk and differentiation and can predict ultimate response to chemotherapy in pts with EOC.

### Tamoxifen in a loading dose schedule in refractory ovarian cancer

R.J. Osborne[1], M.L. Slevin[1], V.J. Harvey[2], J. Shepherd[3], S. Malik[1] & C.J. Williams[4]

[1]*ICRF Department of Medical Oncology, St Bartholomew's and Homerton Hospitals, London, UK,* [2]*Department of Clinical Oncology, Auckland Hospital, New Zealand,* [3]*Department of Gynaecology and Oncology, St Bartholomew's Hospital, London and* [4]*CRC Medical Oncology Unit, Southampton, UK.*

Reports of substantial activity for tamoxifen in refractory ovarian cancer have led to widespread use, particularly because of the drug's low toxicity. However, despite initial enthusiasm, several studies have failed to observe any activity. It is possible that the delayed achievement of therapeutic levels of tamoxifen might permit early tumour progression. The activity of a loading dose schedule known to rapidly achieve stable therapeutic levels of tamoxifen has therefore been investigated in refractory ovarian cancer. Forty-six previously treated patients (mean age 61 yrs, median Karnofsky performance 80%) with stage III or IV measurable ovarian cancer were treated with tamoxifen, $100\,mg\,m^{-2}$ day 1 and $40\,mg\,day^{-1}$ thereafter. Treatment was continued until there was unequivocal evidence of progressive disease. Forty-four patients were assessable for response. There was one partial remission lasting 3 months. Forty patients progressed within 4 months of treatment, 3 patients progressed within 6 months of treatment. Median survival from commencement of tamoxifen was 4 months. No significant toxicity occurred. It is concluded that the activity of tamoxifen in refractory ovarian cancer is minimal.

**Age as an independent prognostic variable in cervical cancer: Survival analysis of 10,018 cases**

C. Meanwell, S. Wilson, K. Kelly, C. Roginski & G. Blackledge

*West Midlands CRC Clinical Trials Unit and Birmingham and West Midlands Region Cancer Registry, UK.*

Recent regional and national data indicate that there has been an increase in invasive cervical cancer registration and death rates in young women (<40 years). Evidence from a number of sources suggest that this group carries a worse prognosis than older women with the disease. The aim of this study was to determine whether youth is an independent and unfavourable prognostic factor, particularly in women diagnosed since the early 1970s. Data were taken from the case-notes and from computerised records of 10,018 women with invasive cervical carcinoma resident within the West Midlands region at the time of diagnosis between 1957 and 1981. Detailed manual and computerised data validation was performed before analyses to ensure completeness and precision of data. Univariate and multivariate survival analyses were performed using a number of patient variables including year of diagnosis, age, duration of symptoms, site of primary tumour, size of primary tumour, clinical stage, histology, presence and site of lymph node and metastatic disease and treatment. Cox regression analysis of survival identified clinical stage, lymph node status, age group and histology as independent prognostic variables. After controlling for other variables, there was a positive association between age and hazard of death from cervical cancer. Using the model, a selection of estimated survival functions were computed. These indicate that in all years, youth was a significant and favourable prognostic factor.

**Active chemotherapy in the treatment of advanced and recurrent cervical cancer**

G. Constantine, C. Meanwell, G. Blackledge, J. Mould, A. Chetiyawardana, D. Spooner, T. Latief, M. Patterson, M. Sokal, C. Alcock, F. Lawton & J. Kavanagh

*Clinical Trials Unit, Queen Elizabeth Hospital, Birmingham B15 2TH, UK.*

Forty-four patients with recurrent or disseminated cervical cancer have been entered into a prospective trial of bleomycin, ifosfamide and *cis*-platinum (BIP). Twelve further patients have received this regimen prior to radiotherapy (RT) in a neoadjuvant setting. In the group with recurrent disease 32 have completed treatment and are evaluable for response. Three of 32 were adeno- and 29/32 squamous carcinomas. Sixteen of 32 had previously received RT alone, 6/32 radical surgery, 9/32 both, and 1/32 no previous treatment. In the neoadjuvant group 6/12 had stage 2, and 6/12 stage 3 disease. Patients were treated with bleomycin (30 mg infused over 24 h), followed by *cis*-platinum ($50\,mg\,m^{-2}$ bolus) and ifosfamide ($5\,g\,m^{-2}$ infused over 24 h), with concomitant hydration (total 10 litres over 3 days) and mesna ($8\,g\,m^{-2}$ given during and for 12 h following ifosfamide) for between 1 and 8 courses (mean = 4).

In the group with recurrent disease 23 (72%) objective responses were seen. Seven women had a WHO complete response, 6 confirmed by CAT scan. At least 70% of patients noted a subjective improvement in disease related symptoms. Seven patients went onto further RT to consolidate the response. Response duration varied between 2 and 11 months with chemotherapy alone (median 4 months). In the neoadjuvant group 9/12 (75%) had at least a 50% reduction in tumour bulk prior to RT. All patients experienced alopecia and nausea/vomiting. Of the evaluable patients 15 (34%) needed one or more blood transfusions and 6 (14%) developed septicaemia. Eight (18%) developed ifosfamide related confusion/disorientation but none were severe. Only one of these was not predictable by the use of a nomogram devised concurrently. Two patients developed *cis*-platinum associated renal damage and there was 1 death partially treatment related from septicaemia.

These data indicate that the BIP combination is highly active in cervical cancer, and may be used for effective palliation and tumour debulking in around 70% of patients with advanced or recurrent disease. Based on these results a multi-centre randomised trial has been launched to determine whether neoadjuvant BIP improves survival in patients with inoperable cancer.

**Relapse following POMB/ACE chemotherapy: Importance of treatment intensity**

S.M. Crawford, E.S. Newlands, G.J.S. Rustin, R.H.J. Begent & K.D. Bagshawe

*Cancer Research Campaign Labs, Department of Medical Oncology, Charing Cross Hospital, London W6 8RF, UK.*

More than 90% of patients with germ cell tumours treated initially with POMB/ACE (*cis-P*latin, *O*ncovin, *M*ethotrexate, *B*leomycin, *A*ctinomycin D, *C*yclophosphamide, *E*toposide) achieve long term remission (Newlands *et al.*, *Br. J. Urology*, **58**, 307, 1986). However, a small number relapse. In order to investigate the effect of variation in the intensity of treatment on the likelihood of relapse, we have conducted a case control study.

Each of 15 patients who received POMB/ACE as their initial chemotherapy and who eventually relapsed was paired with a control who had comparable serum concentrations of alpha fetoprotein and chorionic gonadotrophin and who was treated at about the same time. Indices of chemotherapy were calculated from the doses per $m^2$ given divided by a standard dose. Indices $\alpha$ and $\beta$ describe the drugs whose dose shows most individual variation, $\gamma$ and $\delta$ the remaining drugs. A patient receiving full dose POMB-POMB-ACE-POMB-ACE-POMB-ACE-OMB in the first 4 months has indices $\alpha = 1$ and $\gamma = 1$. Indices $\beta$ and $\delta$ were arbitrarily derived from the mean dose received by the control patients.

$\alpha$ (treatment in first 4 months)

$= (P/480 + M/1500 + E/1500)/3$

$\beta$ (subsequent treatment)
$= (P/68 + M/390 + E/820)/3$

$\gamma$ (treatment in first 4 months)
$= (O/5 + B/150 + A/4.5 + C/1500)/4$

$\delta$ (subsequent treatment)

$= (O/1.8 + B/54 + A/1.8 + C/867)/4$

Using the paired *t*-test, cases *vs.* controls, $\alpha$ ($P=0.015$, median ratio case: control $=0.9$) and $\beta$ ($P=0.035$, median ratio $=0.7$) were significantly less in the cases; the trend was not significant in $\gamma$ ($P=0.568$) or $\delta$ ($P=0.617$).

These findings are consistent with the view that sub-optimum delivery of chemotherapy is a cause of failure.

### An open randomized trial comparing zoladex 3.6 mg depot with stilboestrol 3 mg day$^{-1}$ in advanced prostate cancer: Patient characteristics, response and treatment failures

L.A. Emtage, C. Trethowan, C. Hilton & G.R.P. Blackledge

*For the West Midlands Urology Research Group and at the CRC Clinical Trials Unit, Queen Elizabeth Hospital, Edgbaston, Birmingham, UK.*

A large multicentre randomised study comparing zoladex 3.6 mg depot with stilboestrol (DES) 3 mg day$^{-1}$ in patients with locally advanced or metastatic carcinoma of the prostate is reported. One hundred and twenty-eight patients with previously untreated, histologically confirmed prostate cancer T3 or greater, or any T, M1 have been entered to the study. Prior radiotherapy, other previous malignancy, and incipient spinal cord compression were exclusions. Thirty-nine of 128 (30.5%) patients had a prior history of cardiovascular disease. An analysis of baseline patient characteristics was done to ensure that there was no bias in the randomisation, and no significant difference was found in the two groups when analysed by age, history of cardio-vascular disease, time of diagnosis to time of treatment, symptom status, T status, M status, abnormal haematology or abnormal biochemistry, including acid phosphatase levels.

Follow-up on the first 77 patients at three months reveals a partial objective response rate of 50% ($\pm 16\%$) for zoladex and 52% ($\pm 20\%$) for DES. Subjective responses are also similar for both groups. However 20 patients were withdrawn from DES therapy compared to 4 from the zoladex group due to adverse reaction. This difference is statistically significant ($\chi^2=12.5$, $P<0.001$). Two minor and three major increases in bone pain were found after the first zoladex treatment, compared to none on DES, but none of these required treatment withdrawal. Two patients developed spinal cord compression, one in each treatment group. Both of these were successfully treated with radical radiotherapy and did not require withdrawal from treatment. Insufficient data is available at this stage for proper comparison of duration of response and survival. From this group of patients we show that zoladex is as effective as DES in the treatment of advanced prostate cancer, but is better tolerated than DES. Thirty per cent of these patients had a history of cardiovascular disease, and zoladex is a useful alternative therapy in this group of patients, who tolerate DES poorly.

### Drug resistance and metabolism

### Genomic rearrangements and differential expression in radiation resistant L5178Y murine lymphoma cells

J. Lunec* & D.J. Sherratt

*Institute of Genetics, University of Glasgow, Church Street, Glasgow G11 5JS, UK.*
**Current address: Cancer Research Unit, University of Newcastle-upon-Tyne, Royal Victoria Infirmary, Newcastle-upon-Tyne NE1 4LP, UK.*

Isolates of genomic DNA and polyadenylated mRNA from radiation resistant (L5178YR) murine lymphoma cell lines and the sensitive parental line (L5178YS) from which they were derived, were analysed for evidence of differential expression and possible gene amplification. SDS-polyacrylamide gel electrophoretic analysis of *in vitro* translation products from mRNA preparations gave evidence of differences in the pattern of gene expression between the resistant lines and the sensitive parental line. In particular, a polypeptide of $\sim$50 K was synthesised at an elevated level with mRNA samples from the L5178YR cell lines. Also, when duplicate Southern transfers of genomic DNA digests were hybridised with cDNA probes made by reverse transcription from mRNA derived from L5178YS and L5178YR$_3$ (the most resistant line in the series), differences in the banding pattern were obtained, in which identified DNA fragments from genes transcribed at an elevated level in the resistant line.

A further observation was that a transcribed repetitive element, detected as a 4.9 kb BglII band on blots of genomic digests probed with total cDNA, was reduced in copy number in the resistant lines compared with the sensitive parental line. This reduction was also observed with independently derived resistant lines. The possibility that this is an inducible provirus in this system is being investigated.

The cloning of differentially expressed genes and those involved in observed genomic rearrangements in this system has been initiated.

### A variant of the human lung carcinoma cell line A549 selected for resistance to hydroxyurea

N.C.H. Kerr & A.L. Harris

*Cancer Research Unit, University of Newcastle-upon-Tyne, Royal Victoria Infirmary, Newcastle-upon-Tyne NE1 4LP, UK.*

Non-mutagenised A549 cells were chronically exposed to the ribonucleotide reductase inhibitor hydroxyurea (HU) at stepwise increasing concentrations of drug over a period of 18 mo. Cells routinely cultured in 2 mM HU were cloned and 5 that grew through 2.2 mM HU were selected and grown up, being designated A549 NK-1 to -5. Clone NK-1 cells have doubling times of approximately 33 h in undrugged medium compared with $\sim$22 h for parental cells. In a standard 3 days growth inhibition assay, the $ED_{50}$ for NK-1 cells was 2.4 mM compared to a parental value of 120 $\mu$M HU, i.e. 20-fold resistance to HU. Doubling times in drugged medium for the NK-1 cells were 33 h for up to 1 mM-, 40 h in 1.5 mM- and 52 h in 3 mM-HU. In clonogenic assays, the plating efficiencies in undrugged medium were 0.6% for NK-1 cells compared with 54% for parental cells. In drugged medium, NK-1 cells gave a biphasic response with plating efficiencies of 4% at 0.3–1 mM HU, indicating a dependence on drug for optimal growth. Clone NK-1 cells are far less cross-resistant to two other ribonucleotide reductase inhibitors, 3,4-dihydroxybenzohydroxamic acid (Didox) and 3,4-dihydroxy-benzoamidoxine.HCl (Amidox), being respectively 2.8- and 2-fold resistant. This low level of cross-resistance suggests a changed conformation in the M2 subunit of the ribo-nucleotide reductase in the HU-resistant cells. Karyotype analysis of the NK-1 cells revealed a decrease in chromosome numbers compared to parental cells, with a marker chromosome possibly of homogeneously staining region type, and an absence of double minutes. This is the first report of a human cancer cell line selected for resistance to hydroxyurea.

**Possible induction of pleiotropic drug resistance (PDR) by single agent ifosfamide (Ifos) in children with untreated advanced neuroblastoma (AN)**

S.J. Kellie, J. Pritchard & J. de Kraker

*European Neuroblastoma Study Group (ENSG), The Hospital for Sick Children, Great Ormond Street, London WC1N 3JH, UK.*

Neuroblastoma is an uncommon malignancy and patients (pts) with advanced disease have a relatively poor prognosis. New agents are urgently needed but Phase III studies can take a long time to complete because (a) 'experimental therapy' is often not appropriate in children with recurrent disease and (b) response rates in heavily pretreated pts are often low. In 1984, therefore, the ENSG decided to measure the response rate to single agents at diagnosis, moving on to standard combination chemotherapy (CT) (OPEC; vincristine, *cis*-platinum, etoposide, cyclophosphamide or a variant) after 4–6 weeks. In ENSG study 3A, 35 treatment courses of Ifos ($3\,gm^{-2} \times 2$ with mesna) were administered i.v. to 18 consecutively diagnosed pts (10 male) median age 2.8 yrs, range 1.3–5.3 yrs. The overall response to Ifos was 44% (8/18) using the response definitions of ENSG. Nine pts (50%) had >50% reduction in the volume of primary tumour. Of 15 pts with raised pre-treatment VMA excretion, 6 (40%) responded with >50% reduction. Ten pts were evaluable for bone marrow response. Two pts (20%) had complete clearance of bone marrow involvement. Toxicity was mild in all patients.

Although the initial response rate to Ifos was encouraging, only 4 pts (22%) were in GPR or CR at the completion of 'first line' therapy. Median survival was 9 months. In an earlier study (ENSG 1) pts with AN having similar pre-treatment characteristics who were treated with OPEC from diagnosis had a median survival of 16 months. The difference in median survival between the two studies cannot be explained by dissimilar pts, staging procedures or combination CT. Our data indicates Ifos is an active drug in untreated AN and is associated with mild toxicity. The shortened median survival compared with ENSG 1 is unexplained, however, the induction of PDR with Ifos cannot be excluded.

**Extra-hepatic metabolism of cyclophosphamide: A mechanism of differential tumour cell sensitivity?**

M.C. Walker[1], I.C. Shaw[2], & R.W. Masters[1]

[1]*Institute of Urology, St Paul's Hospital, London WC2, and* [2]*Toxicology Laboratory, University College London, London WC1, UK.*

Using cyclophosphamide (CP) as a model compound we have investigated extra-hepatic metabolism *in vitro*. The differential sensitivity of histologically distinct types of tumour to anticancer prodrugs might be due to differences in their ability, or that of surrounding normal tissue, to metabolically activate these compounds. Traditionally, the liver has been regarded as the site of prodrug activation.

Testicular tumour cells were more sensitive to the cytotoxic effects of CP than bladder tumour cells, following continuous exposure in a colony-forming assay. For example, 1 mM CP killed ~99% of clonogenic cells in a testicular tumour cell line (833 K) studied, compared with 10% cell kill in a bladder cancer cell line (RT112). The metabolites generated by incubation of normal rat tissue homogenates with 1 mM CP were analysed by HPLC. Phosphoramide mustard (PM) (a cytotoxic metabolite of CP) was generated by bladder and testis, in quantities comparable with that produced by liver. Carboxyphosphamide (a non-cytotoxic excretory product) was generated by liver and bladder, but not by testis.

These findings support the hypothesis that differential tumour sensitivity to CP may have a metabolic basis, and also that extrahepatic metabolism is a possible mechanism of prodrug activation.

**Metabolic activation of the 8-(N,N-dimethylcarboxamide) analogue of mitozolomide**

K.R. Horspool[1], A. Gescher[1], C.P. Quaterman[1], M.F.G. Stevens[1], & E. Lunt[2]

[1]*CRC Experimental Chemotherapy Group, Pharmaceutical Sciences Institute, Aston University, Birmingham and* [2]*May & Baker Ltd, Dagenham, Essex, UK.*

Structure-activity studies on various derivatives of the experimental anticancer agent mitozolomide (1) substituted in the 8-carboxamide moiety have shown the requirement of a N-H in this position for maximum cytotoxicity. For example, the monomethyl (2) and dimethyl (3) derivatives of (1) are equiactive with (1) against the TLX5 lymphoma in the mouse *in vivo*. However, (3) is less cytotoxic than (2) or (1) when incubated with TLX5 lymphoma cells *in vitro* ($IC_{50}$: (3) 14.6, (2) 3.0, (1) 2.3 $\mu$M). In order to explain this discrepancy between the high antitumour activity and the low cytotoxicity of (3) its metabolism was studied *in vitro*. The hypothesis for this investigation was that (3) undergoes metabolic N-demethylation to the highly cytotoxic compounds (2) and/or (1). Incubation of (3) ($10\,\mu g\,ml^{-1}$) with mouse liver microsomes and TLX5 cells produced a significant increase in the cytotoxicity of (3). The activation was dependent on the presence of an NADPH-generating system. HPLC analysis of the incubation mixture of (3) with microsomes afforded a peak which co-chromatographed with (2). The amount of (3) converted to (2) within 45 min was 30%. This result suggests that the metabolic production of (2) might account for the high antitumour activity of (3) observed *in vivo*, in spite of its moderate cytotoxicity observed *in vitro*.

1. $R_1 = R_2 = H$
2. $R_1 = Me, R_2 = H$
3. $R_1 = Me, R_2 = Me$

**The metabolic basis for the protective effect of feeding the antioxidant, ethoxyquin, on the hepatocarcinogenic action of aflatoxin $B_1$ in the rat**

H.G. Mandel, G.E. Neal, M.M. Manson & D.J. Judah

*MRC Toxicology Unit, Woodmansterne Road, Carshalton, Surrey SM5 4EF, UK*

The dietary administration of 0.5% ethoxyquin (EQ) has been found to be strongly protective against the hepatocarcinogenic effect of the simultaneous dietary administration of aflatoxin $B_1$ ($AFB_1$). EQ increases the microsomal cytochrome P-450, resulting in an increased rate of *in vitro* $AFB_1$ metabolism and formation of the less toxic $AFB_1$ metabolites, $AFQ_1$ and $AFM_1$. The formation of the active $AFB_1$-epoxide is increased to a lesser extent. These changes in metabolism are paralleled by the pronounced induction of cytochrome P-450 of the P.B.-inducible form.

The cytosol-catalysed conjugation of $AFB_1$-epoxide with GSH is also greatly increased and the binding of [$^{13}$H]-$AFB_1$ to rat liver DNA *in vivo* reduced by EQ (70% inhibition 2 h after dosing with 1 mg $AFB_1$ $kg^{-1}$ body wt.). It is suggested that the reduced DNA binding and hepatocarcinogenesis by EQ treatment results at least in part from increased detoxification in the endoplasmic reticulum and cytosol compartments of the hepatocytes.

### The adverse β-oxidation pathway is markedly inhibited with novel β and γ substituted analogues of chlorambucil

P. Workman[1], M. Oppitz[1], J. Donaldson & P. Coe[2]

[1]*MRC Clinical Oncology Unit, Cambridge and* [2]*Department of Chemistry, Birmingham University, Birmingham, UK.*

The antitumour selectivity of chlorambucil, 4-[4-*bis*-(2-chlorethyl)amino-phenyl]butyric acid (I), is thought to be limited by metabolism through the β-oxidation pathway, resulting in the accumulation of phenylacetic mustard (PAAM). Thus inhibition of this pathway should lead to an improved therapeutic index. We previously found support for this hypothesis with the β,β-difluoro analogue (II) (Lee *et al., Cancer Chemother. Pharmacol.*, **17**, 21, 1986). We now show that metabolism to PAAM can be reduced further by appropriate β and γ substitutions. Drugs were administered i.p. to C3H/He male mice and plasma analysed by a sensitive and specific isocratic, paired-ion reverse-phased HPLC assay with u.v. analysis at 254 and 280 nm.

I

| Compound | Structure β | Structure γ | Plasma (PAAM) 30 min | (μg $ml^{-1}$) 120 min |
|---|---|---|---|---|
| I | H,H | H,H | 14.7 | 8.9 |
| II | F,F | H,H | 6.7 | 4.3 |
| III | F,F | F,F | <0.6 | <0.6 |
| IV | $CH_3$,H | H,H | <0.3 | not determined |
| V | $CH_3$,$CH_3$ | H,H | <0.2 | <0.2 |
| VI | $CF_3$,H | H,H | <0.2 | <0.2 |

The results show clearly that PAAM formation is markedly reduced in all cases. These novel agents may afford a further improvement over analogue II, and antitumour testing is now under way.

### Further evidence that flow cytoenzymological assay of cellular esterase inhibition measures intracellular carbamoylation by chloroethylnitrosourea-derived isocyanates

C. Dive, P. Workman & J. Watson

*MRC Clinical Oncology and Radiotherapeutics Unit, MRC Centre, Hills Road, Cambridge, UK.*

Chloroethylnitrosoureas (CNUs) decompose in an aqueous environment yielding alkylating fragments and isocyanates. While the former cross-link DNA, the latter carbamoylate cellular proteins, a reaction implicated in DNA repair inhibition. We recently reported a novel flow cyto-enzymological technique for the measurement of intracellular carbamoylation based on inhibition of cellular esterases by CNUs. Intrinsic enzyme inhibition and cellular penetration were shown to contribute to extent of inhibition. We now report further evidence that this assay measures intracellular carbamoylation by CNU-derived isocyanates. EMT6 mouse mammary tumour cells were harvested in log phase and exposed to test agents for 1 h, before analysis of esterase kinetics on the Cambridge flow cytometer. Chloroethyliso-cyanate, the decomposition product of BCNU, exhibited inhibitory potency similar to the parent drug. The I50 value (concentration to produce 50% inhibition) was $1.2 \times 10^{-4}$ M, compared to $2.0 \times 10^{-4}$ M for BCNU. Values for CCNU and its metabonate cyclohexylisocyanate were $7.0 \times 10^{-4}$ M and $5.4 \times 10^{-5}$ M respectively. Using conventional spectro-fluorimetry with simple alkyl isocyanates, inhibitory potency was in the order butyl > propyl > ethyl > methyl, demonstrating a clear effect of alkyl chain length. The alkylating agents nitrogen mustard and melphalan failed to inhibit intracellular esterases at concentrations up to $10^{-3}$ M. Thus carbamoylating but not alkylating species cause inhibition of the esterase reaction. Using this assay with novel antitumour agents, we have obtained I50s of $8 \times 10^{-4}$ M for TCNU (similar to CCNU) and $1.5 \times 10^{-3}$ M for temozolomide (slightly more potent than the related mitozolomide), but minimal inhibition for clomesone and cyclodisone.

### Purification, photoaffinity labelling and characterisation of a 4-5S rat cytosolic binding protein specific for 3-methylcholanthrene

P.S. Arnold, R.C. Garner & B. Tierney

*Cancer Research Unit, University of York, Heslington, York Y01 5DD, UK.*

Rat hepatic proteins which sediment at 4 to 5S on sucrose gradients exhibit high affinity (2.5 nM), saturable (700 fmol $mg^{-1}$) binding for the carcinogen 3-methyl-cholanthrene (3MC). A rat liver protein of Stokes radius 3 nm, molecular weight by SDS PAGE of 39,000 and with specific 3MC-binding activity sedimenting at 4.5S, has been purified 315-fold to apparent homogeneity using affinity chromatography on a column of 1-hydroxy-3-methyl-cholanthrene (1-OH-3MC) coupled to epoxy-activated sepharose 6B, in conjunction with two gel filtration steps. The protein purified by this technique was shown to be associated with the observed specific 3MC binding activity by photoactivation using 3-methylcholanthrene-1-one (3MC-1-one). This purified specific 3MC binding protein appears to consist of two or more isozymes (pI 6.1 and 6.2) on 2-dimensional SDS PAGE, and has been characterised with respect to its amino acid composition, its specificity and capacity for binding 3MC. This binding activity has been associated with a regulatory disulfide group on the protein. In addition to characterisation, several monoclonal antibodies have been prepared against this protein.

### Immunocytochemical localisation of VP16 in normal and malignant tissue

H.P. Henneberry & G.W. Aherne

*Department of Biochemistry, University of Surrey, Guildford, Surrey GU2 5XH, UK.*

The peroxidase anti-peroxidase (PAP) technique of immuno-cytochemistry has been used to visualise the distribution of the cytotoxic drug VP16 in fresh-frozen and fixed tissues from normal and tumour-bearing animals. Tissues were obtained from normal mice at various times after high and low dose VP16. Semi-quantitative measurement of intensity and distribution of immunostaining correlated with tissue and plasma concentrations of VP16 measured with ELISA.

The immunostaining method is sensitive enough to detect

VP16 at 1 hr after a 1 mg kg$^{-1}$ dose. Corresponding tissue levels of VP16 were 7.8, 5.7, 2.4, 2.0 $\mu$g g$^{-1}$ in kidney, heart, small intestine and liver respectively and 2.0 $\mu$g ml$^{-1}$ in plasma. The highest dose of VP16 tested (80 mg kg$^{-1}$) produced very intense immunostaining in all organs, corresponding, at 1 hr after dosing, to tissue levels of 828, 678 and 136 $\mu$g g$^{-1}$ in small intestine, kidney and liver respectively. Inter animal variations in tissue concentrations and in immunostaining were minimal.

L1210 leukaemic mice showed increased hepatic accumulation of VP16 and different renal and small intestinal distributions of VP16 compared to normal mice. VP16 immunostaining was also demonstrated in sensitive and multi-drug resistant murine mammary tumour (EMT6) sections (supplied by Dr P. Twentyman). Three of 4 parent tumours examined had positive VP16 immunostaining. Two of 4 resistant tumours also showed some positive immunostaining but this was less intense than in the parent tumour sections. Compared to uptake of VP16 in organs, drug uptake in this tumour type was poor. These results suggest that immunocytochemistry may be a useful technique for studying the distribution of drugs given singly or in combination, specific organ toxicities and mechanisms of resistance.

### Induction of glutathione S-transferase P in liver and kidney

M.M. Manson[1], J.A. Green[1], S.A. Griffiths[1], J.L. Simpson[1] & C.A. Power[2]

[1]*MRC Toxicology Unit, Woodmansterne Road, Carshalton, Surrey SM5 4EF, UK. and* [2]*Ciba Geigy Ltd, Postfach CH-4002, Basel, Switzerland.*

Glutathione S-transferase P (GST-P, 7-7 form) is normally present in rat kidney but not in hepatocytes. Its induction has been described in preneoplastic lesions and tumours where it is considered by some to be a more specific marker for carcinogenesis than glutamyl transpeptidase, which can be induced by many non-carcinogens in a non-focal manner. Using a cloned probe for GST-P (Sugioka *et al.*, *Nucleic Acid Res.*, **13,** 6049, 1985), we have shown induction of mRNA, not only in $AFB_1$-treated liver, but also in a normal liver-derived cell line (BL8) and in primary hepatocytes after 48 h in culture. BL8 cells showed a greatly enhanced induction of GST-P after treatment with $AFB_1$ or transfection with an active *ras* oncogene (Sinha *et al.*, *Cancer Res.* **46,** 1440, 1986). Using polyclonal antiserum (donated by Dr T. Rushmore, University of Toronto) we have shown immunohistochemically that $AFB_1$ treatment (1 ppm, 17 wk) induced 2.5× as many GST-P positive foci as were visualised by GGT histochemistry. Ethoxyquin (0.5% in diet) in combination with $AFB_1$ prevented focal formation, but induced GST-P periportally. In addition to cytoplasmic enzyme activity, many nuclei were also stained. EQ, with or without $AFB_1$, induced GST-P in kidney, but in damaged, hyperplastic or preneoplastic tubules the activity was sometimes low or absent. Variable levels of GST-P were also found in preneoplastic tubules and adenomas resulting from a single injection of dimethylnitrosamine (40 mg kg$^{-1}$). Thus while high levels of induction of GST-P appear to be associated with transformation in the liver, this is not necessarily the case in kidney.

### Differential expression of glutathione transferases in lung tumour samples

J. Carmichael[1], A. Lewis[1], J.D. Hayes[2], D. Lamb[3] & C.R. Wolf[1]

[1]*ICRF, Laboratory of Molecular Pharmacology and Drug Metabolism, Edinburgh,* [2]*Department of Clinical Chemistry, Royal Infirmary, Edinburgh and* [3]*University Department of Pathology, Edinburgh, UK.*

The glutathione S-transferases play a central role in the detoxification of electrophilic chemicals and drugs and there is a growing body of evidence which indicates that they play an important role in resistance of tumour cells to a variety of anticancer drugs. As part of an interest in the regulation of glutathione-dependent enzymes in tumours we have investigated glutathione transferase (GST) expression in a range of normal and tumour lung specimens. Samples of normal and lung tissue were taken from 10 patients undergoing thoracotomy. GST activity was estimated using 3 substrates, CDNB, ethacrynic acid and trans-stilbene oxide. Large variation in GST activity was observed between both lung and tumour samples. GST activity in tumour samples was expressed as a percentage of normal lung activity in each patient. It was extremely interesting that the ratio of GST activity of tumour to normal tissue reflected the tumour type. CDNB activity was elevated 10 fold in the one adenocarcinoma sample and 3-fold in 5 squamous carcinoma samples compared to normal lung. No increase was observed in small cell or large cell anaplastic carcinomas, however. Differences in activity using ethacrynic acid as a substrate were also observed, although these differences did not reflect tumour type. Western blotting showed that all the samples tested contained a considerable amount of the placental (acidic) FST, although the level of this sub-unit did not explain the differences in overall tumour activity. Initial studies indicate the changes in tumour GST expression was due to altered expression of the neutral sub-unit. The above data indicate that study of GST isoenzymes could prove to be a valuable tumour and prognostic marker in cancer therapy.

### Lung cancer and lymphoma

### Prognostic factors in small cell lung cancer: A simple prognostic index is better than conventional staging

M.D. Vincent, S.E. Ashley & I.E. Smith

*Lung Unit, Royal Marsden Hospital, Sutton, UK.*

Conventional staging (CS) in small cell lung cancer (SCLC) is costly, complex, invasive and imperfectly prognostic. We have therefore compared CS with a simple system using presentation features identified by uni- and multi-variate analysis as prognostically important.

Univariate analysis; prognostic significance in 333 patients (1978–1985): $P<0.00005$, plasma albumin, marrow biopsy, Performance Status (PS); $P<0.001$, plasma bilirubin, liver scan (U/S or isotope); $P<0.01$, plasma Na+, gamma GT, alanine transaminase (ala T.) alk. phos.; $P<0.05$, haemoglobin, supraclavicular node, mediastinum, contralateral lung.

Non-significant factors included SVC obstruction, pleural involvement, age sex, WBC and platelets. Multivariate analysis (proportional hazards) identified only plasma albumin, ala. T, liver scan and PS as independently prognostic ($P<0.05$). For simplicity, liver scan was excluded with little effect on prognosis and a prognostic index (PI) was constructed as follows: Group 1 ('good'): albumin

$>36\,g\,l^{-1}$; normal ala T.; PS=0/1 (37% patients). Group 2 ('medium'): Up to 2 of: albumin=30–35 $g\,l^{-1}$; PS=2; abnormal ala T. (41% patients). Group 3 ('bad'): The rest (22% patients).

One year survival (50% 'good'; 27% 'medium'; 3% 'poor') compares well with CS data of 48% for limited disease (LD) and 18% for extensive (ED). Twenty-six per cent of ED patients are in our 'good' prognosis group and 8% of LD patients are in our 'bad' group. Our index gives more detailed prognostic information than CS; it is simpler, cheaper and less invasive.

### Increased detection of micrometastases in the bone marrow of small cell lung cancer (SCLC) patients

F.G. Hay[1], A. Ford[2] & R.C.F. Leonard[1]

[1]*Department of Clinical Oncology and* [2]*Department of Haematology, Western General Hospital, Edinburgh, EH4 2XU, UK.*

Rapid and accurate detection of metastases in the bone marrow of patients with SCLC has important therapeutic implications. Analysis of bone marrow is done routinely by conventional histological examination of marrow smears which has a good detection rate where foci of tumour cells are present or large numbers of cells infiltrate the marrow but is less satisfactory where single cells are sparsely distributed throughout the marrow. We report on a series of 18 bone marrows from SCLC patients that were assessed for tumour infiltration in several different ways – routine histology; cytospin preparations of red cell depleted marrow with immunohistology using 2 MoAbs ($HMFG_2$, CAM5.2); detailed examination of multiple air dried preparations of red cell depleted bone marrow with a panel of 10 SCLC-associated MoAbs and capacity to grow in serum-free defined media (Hites). In 4/18 cases the routine marrow examination revealed tumour infiltration and in 6/18 of the cytospin preparations tumour cells were seen. Using the larger panel of MoAbs a further 9 cases were thought suspicious of tumour involvement although only isolated cells were detected. In 8/9 of these we sustained growth of cells morphologically similar to the putative tumour infiltrate in Hites medium for periods of 1–18 wks and 2 remain viable as established cell lines. These results indicate that detailed analysis of bone marrow using a panel of MoAbs may be worthwhile particularly in programmes utilising autologous marrow rescue as intensification therapy.

### Randomised comparison of treatment duration in small cell lung cancer (SCLC)

H. Earl[1], R.L. Souhami[1], C.M. Ash[1], S.G. Spiro[2], D. Geddes[3], P.G. Harper[4] & J.S. Tobias[1]

[1]*Department of Radiotherapy and Oncology, University College Hospital, London WC1 6AU,* [2]*Brompton Hospital, London SW3 6HP,* [3]*London Chest Hospital, London E2 9JX and* [4]*Guy's Hospital, London SE1 9RT, UK.*

Six hundred and fourteen patients with SCLC entered a randomised trial comparing different initial treatment durations, and the value of chemotherapy on relapse. Patients were stratified according to stage (limited or extensive), and randomised to receive either 4 or 8 courses of chemotherapy (cyclophosphamide $1\,g\,m^{-2}$ day 1, vincristine 2 mg day 1, etoposide 100 mg tds days 1–3), 3 weekly. At presentation patients were also randomised for treatment at relapse, either to receive further chemotherapy (adriamycin $50\,mg\,m^{-2}$, and methotrexate $50\,mg\,m^{-2}$, every 3 weeks) or symptomatic treatment alone.

Response rates to short (S) and long (L) initial chemotherapy were the same (S=64%, L=64%), as were response rates to relapse chemotherapy (S=26%, L=22%). Overall median survival (MS) was slightly better in patients receiving 8 rather than 4 courses of chemotherapy (MS 39 *vs.* 33 weeks, $P=0.05$). Progression free interval (PFI) was longer in patients receiving 8 courses (median PFI 8 *vs.* 4 courses, 31 *vs.* 23 weeks, $P=0.0001$). Survival from relapse was longer for patients receiving relapse chemotherapy than for those receiving symptomatic treatment alone (MS 17 *vs.* 12 weeks, $P=0.00001$). The only treatment strategy associated with a significantly worse survival was short initial chemotherapy and no chemotherapy on relapse (MS 30 *vs.* 39 weeks, $P=0.008$). Other treatment strategies gave equal results.

### Randomised trial of planned *versus* as required chemotherapy in small cell lung cancer (SCLC)

R.L. Souhami[1], H. Earl[1], C.M. Ash[1], S.G. Spiro[2], D. Geddes[3] & P.G. Harper[4]

[1]*Department of Radiotherapy and Oncology, University College Hospital, London WC1 6AU,* [2]*Brompton Hospital, London SW3 6HP,* [3]*London Chest Hospital, London E2 9JX and* [4]*Guy's Hospital, London SE1 9RT, UK.*

Patients with SCLC with poor prognostic features (Souhami *et al., Cancer Res.*, **45**, 2878, 1985) have been treated in a continuing trial of chemotherapy employing 2 different approaches to treatment. Following an initial cycle of chemotherapy (cyclophosphamide $1\,g\,m^{-2}$ day 1, vincristine 2 mg day 1, etoposide 100 mg bd days 1–3) patients with responding or stable disease are treated with the same chemotherapy in one of two ways. One group receives chemotherapy in planned 3 weekly cycles, the other receives chemotherapy only when there is disease progression: patients with responding or stable disease who are asymptomatic are not treated but are seen every 3 weeks and reassessed. One hundred and five patients have been randomised up to November 1986. In the 'as required' arm the median treatment-free interval between course 1 and course 2 was 47 days; course 2 and 3: 44 days; course 3 and 4: 46 days; course 4 and 5: 44 days. These intervals are over twice as long as the 'planned' chemotherapy. So far there is no difference in survival in the two arms. Quality of life assessments of the two forms of treatment are in progress. This trial represents a new approach to palliative treatment of SCLC.

### A randomised trial of short courses of intravenous chemotherapy *versus* oral out-patient chemotherapy for small cell lung cancer (SCLC)

B. Cantwell[1], P. Corris[2], J. Bozzino[1] & A.L. Harris[1]

*North East Lung Cancer Group,* [1]*University Department of Clinical Oncology, Newcastle General Hospital and* [2]*Chest Unit, Freeman Hospital, Newcastle on Tyne, UK.*

A regional trial comparing short courses of i.v. chemotherapy given in hospital with out-patient chemotherapy for SCLC was activated in March 1984 and 215 patients have now been entered. Eligibility criteria included histologically and/or cytologically proven SCLC, assessable disease and WHO performance status $\leqq 2$. Patients were stratified by extent of disease. Course 1 i.v. chemotherapy was doxorubicin $40\,mg\,m^{-2}$, vincristine 2 mg, VP16 $100\,mg\,m^{-2}$ i.v. and 300 mg p.o. on days 2 and 3. Courses 2, 3 and 4 were doxorubicin $30\,mg\,m^{-2}$, vincristine 2 mg, VP16 $200\,mg\,m^{-2}$, plus infusional ifosfamide and mesna

$5\,g\,m^{-2}$. Oral chemotherapy was chlorambucil $6\,mg\,m^{-2}$ daily, procarbazine 50 mg t.d.s. and E.C. prednisolone 10 mg b.d., all for 10 days with VP16 300 mg daily on days 1, 2 and 3. Both i.v. and oral chemotherapy were given every 3 weeks to a total of 4 courses, after which prophylactic cranial and consolidation chest radiotherapy was given to good responders, in both arms. Analysis of survival up to a maximum of 32 months in the first 150 patients entered shows highly significant survival advantage to 103 limited stage *vs.* 47 extensive stage patients ($P<0.01$, log rank test). There were no significant differences in survival between 76 i.v. and 74 orally treated patients overall or when subdivided by stage. Interim analysis indicates that oral out-patient chemotherapy confers similar early survival benefit to more expensive hospital based i.v. chemotherapy.

**First-line chemotherapy re-challenge after relapse in small cell lung cancer**

M.D. Vincent, B.D. Evans & I.E. Smith

*Lung unit, Royal Marsden Hospital, Sutton, Surrey, UK.*

Conventionally, relapsed SCLC patients are offered alternative, allegedly non cross-resistant chemotherapy (CT). To question the assumption that these cancers are resistant to their initial CT, we re-challenged 15 SCLC patients who had relapsed off treatment with the identical regimens used at induction. Presentation mean age was 63 years (yrs) (57–73 yrs) and 7 had extensive disease (ED). At re-challenge, 13 had ED and 3 were ECOG performance status (PS) 1, 7 were PS 2 and 5 were PS 3. Thirteen were male. Three CT regimens were used as follows:

Regimen 1 (10 patients): carboplatin (C) $300\,mg\,m^{-2}$ i.v. day 1, etoposide (VP16) $100\,mg\,m^{-2}$ i.v. days 1–3, q. 28 days × 4 cycles.

Regimen 2 (3 patients): adriamycin (A) $40\,mg\,m^{-2}$ i.v. and vincristine (V) 2 mg i.v. day 1; VP16 $100\,mg\,m^{-2}$ i.v. days 1–3, q. 21–28 days × 4 cycles.

Regimen 3 (2 patients): C $400\,mg\,m^{-2}$ q. 28 days × 5 cycles (1 patient) and C $800\,mg\,m^{-2}$ q. 28 days × 2 cycles (1 patient).

Ten of 15 patients (66%) achieved a second response as follows:

| *Regimen* | *Drugs* | *Patients* | *First responses* | *Second responses* |
|---|---|---|---|---|
| 1 | C, VPI6 | 10 | 4 DR, 6 PR | 6 PR |
| 2 | A, VP16, V | 3 | 2 CR, 1 PR | 2 PR |
| 3 | C | 2 | 0 CR, 2 PR | 2 PR |

Median response duration was 3 months (2–4). Responses were more durable if first responses had lasted >8 months; extent of first response had no influence on length of second response. Relapsed SCLC has a good chance of responding to first-line re-challenge CT, although responses are temporary and incomplete. This indicates that relapse is not entirely a consequence of emergent drug resistance, and questions the concept of non-cross resistant CT.

**Do some patients never require treatment for advanced low grade non-Hodgkin's lymphoma?**

M.E.R. O'Brien[1], P. Easterbrooke[2], I.C.M. MacLennan[3], J.M. Holt[4], G.R.P. Blackledge[1] & R.C.F. Leonard[5]

[1]*CRC Clinical Trials Unit, Queen Elizabeth Hospital, Birmingham,* [2]*Department of Medicine, St George's Hospital, London,* [3]*Department of Immunology, University of Birmingham,* [4]*Lymphoma Clinic, Churchill Hospital, Oxford and* [5]*Department of Clinical Oncology, Edinburgh, UK.*

One hundred and eighty-two patients with low grade non-Hodgkin's lymphoma (NHL) were analysed to study the natural history of the disease. Median follow up 70 mths (range 18–199).

Sixty-nine patients had been allocated treatment according to the presence or absence of B symptoms (Stage I excluded) in a randomised trial. The remaining 113 were managed using a watch and wait policy in the absence of symptoms or rapidly progressing disease (Stage II – 18, Stage III – 44, Stage IV – 92).

Fifty-six patients received no treatment at the time of diagnosis. Of these 23 were allocated the no treatment arm of a 'no treatment *vs.* chlorambucil' randomisation (B symptoms excluded), the other 33 received no treatment by attending physicians' choice.

Eleven of 23 and 11/33 have not yet required systemic treatment, median follow up 70 mths (range 18–199).

This group of never-treated patients had no special characteristics in terms of age, sex, presenting symptom, pathology or sites of involvement and did not differ from the patients who later required treatment except Stage IV disease was more common in the latter group $P=0.03$. Median time until treatment was required 39 mths (range 4–108 mths) patients. The treatment free interval is similar to that reported by the Stanford group (1984) but survival is significantly different, 49% 5 year survival (confidence intervals 34–64%).

From this we conclude there are no established prognostic variables which will characterise low grade NHL patients who will never need treatment but by adopting a policy of initial no treatment in this group, at least 14% (22/154) of patients may never require treatment.

**Phase II and Phase III studies of weekly chemotherapy for high-grade non-Hodgkin's lymphoma – A new regime (CAPOMEt)**

N.S.A. Stuart, J.A. Child, G.R.P. Blackledge, A.V. Simmons, J. Fletcher, D.L. Barnard, M.H. Cullen & L.A. Parapia

*For the Central Lymphoma Group (Co-ordinating Centre, CRC Clinical Trials Unit, Queen Elizabeth Hospital, Birmingham, UK).*

Recent studies have suggested that short-course weekly chemotherapy (CT) may produce a higher complete response (CR) rate in high-grade non-Hodgkin's lymphoma (HG-NHL) than standard cyclical CT and may lead to increased long-term survival. We have treated 28 patients (pts) with HG-NHL and 5 with 'aggressive' low-grade NHL with a weekly CT regime (CAPOMEt) comprised thus: Week 1, CA = cyclophosphamide $400\,mg\,m^{-2}$ + adriamycin $50\,mg\,m^{-2}$ both i.v. day 1. Week 2, PO1 = vincristine 2 mg i.v. day 1 + prednisolone $60\,mg\,m^{-2}$ + ranitidine 150 mg b.d. both p.o. days 1–5. Week 3, MEt = methotrexate $250\,mg\,m^{-2}$ + etoposide $100\,mg\,m^{-2}$ both i.v. day 1 + etoposide 150 mg p.o. days 2 and 3 + oral folinic acid 15 mg qds p.o. days 2–5. Week 4, PO2 as week 2. The regime repeats for 13 treatments, or if CR is not achieved by 4 weeks to 17 treatments in each case ending with CA. Whenever possible CT continues weekly with dose reduction for myelosuppression rather than delay. Pt characteristics were as follows: mean age 54 (15–74), Stage IV 19/30, bone marrow involved 5/32.

Mean doses given were as follows: C, 365 mg m$^{-2}$; A, 45 mg m$^{-2}$; P, 58 mg day$^{-1}$; O, 1.9 mg; M, 226 mg m$^{-2}$; Et, 400 mg (i.v. + p.o.). M and Et were the drugs most often modified; number of times on which less than 75% of protocol dose was given were: M, 15/75; Et 22/74; C, 15/88; A, 14/88; P, 9/141; O, 7/141. In all 82/608 (13%) of treatments were modified. The commonest reasons for dose modifications were leucopenia (18/56) and the patients' age or poor general condition (13/46). Mean days delay for each part of the cycle were CA, 1.3; PO1, 0.4; MEt, 1.2; PO2, 0.2. In all 32/276 (11%) of treatments were delayed. The commonest reason for delay was leucopaenia (11/32). Mean WBC (neutrophils) $\times 10^9 l^{-1}$ prior to each phase of treatment were: CA, 8.1 (5.3); PO1, 4.7 (3.1); MEt, 6.1 (3.6); PO2, 4.7 (3.3) Grade 4 thrombocytopaenia was seen on only 4 occasions. Other toxicities experienced were: severe neuropathy, 2/31; mucositis, 5/31; septicaemia, 3/31; chest infections, 8/31; blood transfusion for anaemia, 8/31. There was one death to which treatment contributed. CT was continued to 16 weeks in 10 pts, to 12 weeks in 10 pts and was stopped before 12 weeks because of toxicity in one and because of death or disease progression in 10. CR rate for HG-NHL was 67% (95% C.I. 48%–86%).

Toxicity of this regime is acceptable and mainly comprises neutropaenia. CT can be given weekly on the majority of occasions. MEt was the treatment most often modified but in view of the blood counts 7 and 14 days later many of these modifications may have been unnecessary. We have begun a multi-centre, prospective, randomised trial comparing CAPOMEt with standard cyclical CT (CHOP-MTX). Major end-points will be toxicity, response rates, and long-term survival. Forty pts have been randomised in the first 6 months of the study which should end in 3 years.

### Antiemetics

### A randomised trial of oral nabilone and prochlorperazine compared to intravenous metoclopramide and dexamethasone in treatment of emesis induced by chemotherapy regimens containing *cis*-platin of *cis*-platin analogues

D. Cunningham[1,2], C.J. Bradley[1], C.J. Forrest[1], A.W. Hutcheon[3], L. Adams[1], M. Sneddon[2], M. Harding[1], D.J. Kerr[1], S.B. Kaye[1] & M. Soukop[2]

[1]*Glasgow University, Department of Medical Oncology,* [2]*Department of Medical Oncology, Glasgow Royal Infirmary and* [3]*Woodend General Hospital, Aberdeen, UK.*

Seventy-nine patients entered this randomised, open, cross-over trial. All were receiving their first course of chemotherapy with regimens containing *cis*-platin of *cis*-platin analogues. Nabilone (N) 2 mg and prochlorperazine (P) 5 mg were given orally beginning 6 h before chemotherapy and continued 12 hourly for 3 or 4 doses. Metoclopramide (2 mg kg$^{-1}$) (M) was given as i.v. loading dose, followed by i.v. infusion (3 mg kg$^{-1}$) over 8 h after chemotherapy, combined with dexamethasone (D) 20 mg i.v. over 3–5 min at the time of chemotherapy.

With N and S there was complete control of vomiting in 23 patients (31.5%) (median number of episodes of vomiting 3.45 ± 0.78) compared to complete control of vomiting in 40 patients (55.6%) treated with M and D (median number of episodes 3.92 ± 0.54) ($P<0.05$). Eighteen patients (25.4%) on N and S did not feel nauseous, compared to 26 patients (36.1%) on M and D. The score of nausea on a linear analogue scale was significantly better ($P=0.018$) with M and D. With regard to toxicity, sedation was more frequent with N and P ($P<0.01$) as was dryness of mouth ($P<0.001$) and dizziness ($P<0.001$). Extrapyramidal reactions were more common with M and D ($P<0.001$). Dysphoria occurred in 20% of patients given N and S (but this was severe in only 4.1%) and in 2.6% of patients given M and D. There was no overall patient preference for either antiemetic. (N and P = 27; M and D = 29; 12 no preference). For patients receiving *cis*-platin, M and D is a more effective antiemetic than N and P and is associated with fewer side effects.

### Comparison of the antiemetic efficacy of domperidone, metoclopramide and dexamethasone in out-patients receiving chemotherapy

D. Cunningham[1], C. Evans[1], A. Pople[1,2], J. Dearling[1], D. Chappell[1,2] & R.C. Coombes[1,2]

[1]*Medical Oncology Unit and* [2]*Ludwig Institute for Cancer Research, St George's Hospital Medical School, London SW17 0RE, UK.*

Sixty consecutive patients receiving out-patient chemotherapy regimens without *cis*-platin entered the trial. Five had received previous chemotherapy. There was pre-randomisation stratification into regimens of high and low emetogenic potential, before patients were allocated to 1 of 3 antiemetics. Domperidone (D) 20 mg tds or metoclopramide (M) 20 mg tds were given orally on the day before chemotherapy and continued for 3 days with 20 mg bd in day 4 and 20 mg on day 5. On the day of chemotherapy the second dose of M was given as an i.v. bolus. Dexamethasone (Dex) was given orally 4 mg tds on the day before chemotherapy and continued for 3 days with 4 mg bd on day 4 and 4 mg od on day 5. On the day of chemotherapy the second dose was given as an i.v. bolus. If the first regimen failed to control nausea and vomiting, patients were randomly allocated to one of the remaining regimens, and if that failed they were allocated to the third regimen.

There was complete control of nausea (N) and vomiting (V) in 5 (25%) of the patients given M compared to 8 (40%) with D and 12 (60%) with Dex. V was significantly less with Dex compared to M ($P=0.005$), but other comparisons were not statistically significant. After failing first-line antiemetic treatment 3 patients were given M as a second option but all experienced N and V. Also, 3 patients given D as a second option experienced V. No patient was given M or D as a third option. Six patients were given Dex as a second option and only 1 experienced N and V. Of 2 patients given Dex as a third option 1 experienced N and V. Side effects profiles were similar for each antiemetic including dyspepsia. There were no recorded extra pyramidal reactions. Dex is a superior antiemetic to M and may also be effective for patients who fail to respond to D.

### β endorphine – a modulator of emesis in patients receiving chemotherapy?

G.J. Forrest[2], D. Wallace[2], D. Cunningham[2], T. Young[1], Y. Perry[2], C. Gray[2], F. Ballantyne[2], G. Beastall[2] & M. Soukop[1]

[1]*Department of Medical Oncology, Glasgow Royal Infirmary and* [2]*Department of Biochemistry, Glasgow Royal Infirmary, Glasgow, UK.*

The pathogenesis of cytotoxic induced emesis is still poorly understood. Harris *et al.* (*Lancet*, **i**, 714, 1982) have suggested that cytotoxic drugs interfere with the production of enzymes which degrade enkephalins, resulting in a rise in their level in the blood and CSF, stimulating the vomiting centre and inducing emesis. We have therefore measured the levels of $\beta$ endorphine and (met) enkephalin in peripheral blood in six patients receiving combination chemotherapy, just prior to and for 12 h after the i.v. administration of cytotoxic agents. $\beta$ endorphine and (met)enkephalin were measured using a radio-immunoassay technique with sensitivities of 5 pmol l$^{-1}$ and 2 pmol l$^{-1}$ respectively.

In 2 patients who experienced no nausea or vomiting, there was no rise in $\beta$ endorphine or (met)enkephalin with a main peak level of 3.85 pmol l$^{-1}$ $\pm$0.97 (normal range 4–6 pmol l$^{-1}$). The remaining 4 patients all experienced nausea and vomiting after cytotoxic administration. In all patients, the level of $\beta$ endorphine in the peripheral blood rose $\sim$30 min prior to the onset of nausea and vomiting. The mean peak level of endorphine reached was 22.2 pmol l$^{-1}$ $\pm$4.68. The level of (met)enkephalin did not significantly change in any of the patients, remaining within the normal range (20–200 pmol l$^{-1}$) before or after chemotherapy.

These observations seem to suggest that changes in $\beta$ endorphine levels may be important in the physiological mechanisms of nausea and vomiting. The mechanism of action remains unclear and requires further study. However, this information may be used to provide a more rational antiemetic approach in the future.

### Anti-emetic activity of BRL 43694, a novel $5HT_3$-receptor antagonist

E.A. Boyle, W.D. Miner & G.J. Sanger

*Beecham Pharmaceuticals, The Pinnacles, Harlow CM19 5AD, UK.*

We have suggested (Miner & Sanger, *Br. J. Pharmac.*, **88,** 497, 1986) that the anti-emetic activity of high dose metoclopramide (Mcp; Maxolon, Beecham) is due to $5HT_3$-receptor antagonism. Using male ferrets, we now describe the anti-emetic actions of a much more potent and selective $5HT_3$-receptor antagonist, BRL 43694. Routinely, compounds were given by venous cannulae and the ferrets were observed for 120 min (post X-ray) or 240 min (post cytotoxic drugs).

BRL 43694 or Mcp were given by divided dose 30 min before and 45 min after *cis*-platin (7 or 10 mg kg$^{-1}$). Mcp dose-dependently reduced the number of emetic episodes (89% reduction at 2 x 2.5 mg kg$^{-1}$). BRL 43694 reduced emesis by 93%, 96% and 100% at 2 x 0.005, 2 x 0.05 and 2 x 0.5 mg kg$^{-1}$ respectively. BRL 43694 2 x 0.5 mg kg$^{-1}$ also protected against the emesis evoked by doxorubicin/cyclophosphamide (6/80 mg kg$^{-1}$), being more effective (96% inhibition) than 2 x 2.5 mg kg$^{-1}$ Mcp (48% inhibition). A single injection of BRL 43694 0.5 mg kg$^{-1}$ 10 min before exposure to an X-ray source ($\sim$3 Gy min$^{-1}$; Machlett model OEG–50; 50 kV/20 mA, tungsten anode) abolished emesis. An oral dose of BRL 43694 (0.5 mg kg$^{-1}$) reduced radiation-evoked emesis by 94% when given 60 min before exposure. For all emetic stimuli tested, a single dose of BRL 43694 (0.5 mg kg$^{-1}$ i.v.) arrested vomiting within 15 to 60 sec when given during an emetic episode.

BRL 43694 is well tolerated in the ferret, does not cause sedation and shows potent anti-emetic activity by either the i.v. or oral routes.

### The effect of metoclopramide on the bioavailability of TCNU

P.S. Warrington, J.S. Macpherson & J.F. Smyth

*Medical Oncology Unit, Western General Hospital, Edinburgh EH4 2XU, UK.*

TCNU (T) is a new, orally administered nitrosourea which causes dose-related nausea and vomiting. The anti-emetic metoclopramide (M) alters the bioavailability of T. Nine patients were entered into the study. Anti-emetics were not prescribed with the first course of T but all patients received M (30 mg i.v. bolus) 20 min before the administration of their second treatment course. Plasma samples analysed by HPLC showed a significant increase in the Cpmax of T when given with M and the Tmax was significantly reduced. These results could have clinical significance if activity or toxicity are related to peak concentration and highlight the need for standardising anti-emetic treatment in multicentre trials.

| | *Cpmax ng ml$^{-1}$* | | *Tmax (min)* | |
|---|---|---|---|---|
| *Patients* | *T* | *T+M* | *T* | *T+M* |
| 1 | 957 | 1865 | 60 | 15 |
| 2 | 2913 | 2493 | 30 | 30 |
| 3 | 668 | 1389 | 60 | 45 |
| 4 | 861 | 1841 | 120 | 45 |
| 5 | 1339 | 1388 | 30 | 15 |
| 6 | 1092 | 1356 | 30 | 45 |
| 7 | 1202 | 2534 | 30 | 30 |
| 8 | 934 | 1219 | 60 | 30 |
| 9 | 890 | 1827.5 | 60 | 30 |

$P<0.05$ Wilcoxon signed rank test

### The pharmacokinetics of morphine and morphine glucuronides in normal volunteers after 4 routes of administration

R.J. Osborne, S.P. Joel & M.L. Slevin

*ICRF Department of Medical Oncology, St Bartholomew's and Homerton Hospitals, London, UK.*

Morphine is the most important analgesic used to treat cancer pain. The metabolite morphine-6-glucuronide (M6G) is known to have pharmacologic activity and, in animals, its potency is several times *greater* than that of morphine. Investigation of the pharmacokinetics of this metabolite may cast some light on the relative contribution of morphine and M6G to the clinical effects of morphine treatment. The pharmacokinetics of morphine and morphine glucuronides (M6G, M3G), have been determined in 5 volunteers after administration of morphine intravenously (i.v.), and as oral (p.o.), buccal (B) and sublingual (SL) tablets. A specific HPLC assay was used, with fluorescence and electrochemical detection (*J. Chromatogr.* **375,** 174, 1986). The table shows mean AUC (ng ml$^{-1}$ . h mg$^{-1}$ dose, corrected to 70 kg), mean time to peak concentration (t.max) (h), and *median* terminant elimination half life (t.$\frac{1}{2}$) (h).

| | *Morphine* | | | *M6G* | | | *M3G* | | |
|---|---|---|---|---|---|---|---|---|---|
| *Route* | *AUC* | *t.max* | *t.$\frac{1}{2}$* | *AUC* | *t.max* | *t.$\frac{1}{2}$* | *AUC* | *t.max* | *t.$\frac{1}{2}$* |
| i.v. | 8.5 | – | 1.6 | 17.7 | 0.8 | 2.7 | 99.3 | 0.2 | 4.8 |
| p.o. | 1.8 | 0.85 | 1.5 | 14.1 | 1.4 | – | 89.7 | 1.1 | – |
| SL | 1.9 | 1.5 | 1.9 | 15.3 | 2.2 | – | 89.8 | 2.1 | – |
| B | 1.8 | 2.0 | 2.4 | 12.7 | 2.9 | – | 61.3 | 2.9 | – |

M6G is more abundant than morphine and persists longer than morphine (t.$\frac{1}{2}$ for M6G and M3G showed considerable variability – *range* 1.3–17.8 h). If data from animal studies for analgesic activity of M6G can be substantiated in man, these results suggest that M6G is the major active component of non-parental morphine (M6G:M AUC ratio = 7.6) and that it contributes substantially to the duration of activity of both i.v. and non-parenteral morphine.

### An easy (ESI) approach to assessment and quality of life in cancer patients

S. Bindemann[1] & M. Soukop[2]

[1]*Phoenix Foundation, Ross Hall Hospital, Glasgow and* [2]*Department of Medical Oncology, Glasgow Royal Infirmary, Glasgow, UK.*

Anxiety and depression are 'markers' to psychological and psychiatric morbidity, of universal acceptance and value. Accurate assessment of these states should therefore possess predictive value, so far as quality of life in cancer patients is concerned. In order to achieve precise measurement in large populations, a questionnaire, capable of providing an unambiguous score indicating reactive (appropriate) anxious or depressed state, through adjustment disorder, to evidence of major psychopathology, would be required. Items diagnostically associated with anxiety and/or depression, but describing physical symptoms of advanced malignancy, would be excluded. The Effects of Serious Illness (ESI) (pronounced 'easy') Scale is a self-report questionnaire, which incorporates subscales for the measurement of Anxiety and Depression (Form A) and Physical Status (Form B). Individual items (32 in all) are rated on 'frequency' and 'severity/intensity' criteria and the ESI Scale incorporates a readily applied method for scoring and the assessment of internal reliability and consistency. A readily applied scoring stencil has also been developed. Early data shows a high correlation (+0.8) when compared with scores obtained from the same population via the Leeds Anxiety and Depression Scales (LSA, LSD). Normative data relating to 'normal range', 'appropriate reactive state' and 'evidence of pathology' will also be reported. The ESI Scales show considerable promise as a quick and accurate screening instrument for psychological morbidity in diverse patient populations.

### Reversal of cachexia induced by a transplantable colon tumour in mice by flavone acetic acid (LM 975)

M.C. Bibby & J.A. Double

*Clinical Oncology Unit, University of Bradford, West Yorkshire BD7 1DP, UK.*

MAC 16 is one of a panel of tumours initially induced by 1,2-dimethyl hydrazine. Like the majority of the other tumours in this series it is a slow growing adenocarcinoma, but unlike the others it has large areas of necrosis and its growth is accompanied by severe body wasting in the host. Initial chemotherapy studies showed MAC 16 to be only poorly responsive to standard agents. These observations suggest MAC 16 may be the most clinically relevant of the MAC series. LM 975 has been shown to be highly active in established solid transplantable tumours (Bibby *et al.*, *Br. J. Cancer*, **55**, 159, 1987). This study examines the chemosensitivity of MAC 16 to a series of cytotoxic agents including LM 975. Tumours are transplanted by implantation of trocar fragments in the flank. Chemotherapy commences when tumours can be reliably measured and anti-tumour effects are assessed by serial twice weekly caliper measurements. The tumour was unresponsive to cyclophosphamide, 5-fluorouracil and ThioTEPA contrary to our previous findings (Ali *et al.*, *Br. J. Cancer*, **52**, 452, 1985) and also to chlorambucil, DTIC, methylCCNU and mitozolomide. LM 975 proved more toxic to mice bearing the MAC 16 tumour than those bearing other MAC lines, however significant anti-tumour activity was seen. Responses were accompanied by reversal of the cachexia similar to that seen following surgical excision. The alteration in chemosensitivity to standard agents might be explained because serial passage was biased by selection for the cachexia. The resultant tumours are also more ischaemic and necrotic and this may influence drug bioavailability. These results would suggest that LM 975 has a novel mechanism of action which is possibly dependent on specific biological properties of the tumours.

### Posters

### Enhanced recognition of colorectal tumours using combinations of monoclonal antibodies

L.G. Durrant[1], K.C. Ballantyne[3], V. Byers[2], J.D. Hardcastle[3] & R.W. Baldwin[1]

[1]*Cancer Research Campaign Laboratories, University of Nottingham, Nottingham, UK,* [2]*XOMA Corporation, Berkeley, CA, USA and* [3]*Department of Surgery, Queens Medical Centre, Nottingham, UK.*

Several monoclonal antibodies (Mabs) recognising tumour associated antigens are currently being evaluated for therapy of human cancer. Variations in antigen expression on tumour cells could produce difficulties in recognition of cell tumours. One approach to this problem is to use a combination of Mabs which recognise several distinct surface epitopes.

Immunophenotypes of 50 primary colorectal tumours have been analysed to select an appropriate combination of Mabs. The optimum combination of Mabs would consist of C14 recognising Y haptenic blood group, 791T/36 recognising 791T p72 antigen, 365 recognising carcinoembryonic antigen and B14/B8 recognising normal cross reacting antigen. This combination would recognise all of the colorectal tumours, 60% of them binding in all 4, 18% any 3, 12% any 2 and 10% binding a single Mab.

Binding and cytotoxicity of monoclonal antibody-ricin A conjugates to MKN45 cells.

| *Antibody* | *Immunofluorescence* Mean fluorescence | *Immunofluorescence* % positive cells |
|---|---|---|
| 228 | 527 | 75 |
| B14/B8 | 427 | 77 |
| 791T/36 | 141 | 68 |
| 228 + B14/B8 | 1064 | 87 |
| 228 + 791T/36 | 717 | 76 |
| 791T/36 + B14/B8 | 738 | 76 |
| 791T/36 + B14/B8 + 228 | 1342 | 86 |

*Cytotoxicity*

| *Drug or conjugate* | $IC_{50}[RTA]$ $ng\,ml^{-1}$ |
|---|---|
| Free RTA | 1250 |
| 228-RTA | 175 |
| B14/B8-RTA | 40 |
| 791T/36-RTA | 483 |
| 228RTA + B14/B8-RTA | 50 |
| 228RTA + 791T/36RTA | 200 |
| 791T/36RTA + B14/B8RTA | 75 |
| 791T/36RTA + B14/B8RTA + 228RTA | 100 |

When cultured cells were stained with these Mabs the intensity of binding and the number of cells recognised was increased by using combinations of Mabs. When cells were treated with ricin A toxin (RTA) conjugated to 3 different antibodies, it was almost as cytotoxic as when it was conjugated to the most effective single antibody.

Inter and intra tumour heterogeneity can be reduced by using combinations of Mabs recognising distinct epitopes.

### Red cell filterability in cancer patients

M.L. Slevin, S.P. Joel, A. Bramley, M. Schofield, D. Trew, C. Lewis, W. Gregory & P.F.M. Wrigley

*ICRF Department of Medical Oncology, St Bartholomew's and Homerton Hospitals, London, UK.*

It has been suggested that red cell filterability (RCF) may be reduced in cancer patients and this is related to stage of disease; also that priming with chemotherapy prior to treatment corrects RCF and that this improves treatment results. In order to investigate this, RCF has been studied in 100 cancer patients at a diagnosis and 100 age/sex and time matched controls (Study A). In a further study 15 small-cell lung cancer (SCLC) patients receiving single agent etoposide (total dose $500\,mg\,m^{-2}$) had RCF measured before, during and after cycle 1 of chemotherapy (Study B). RCF measurements were made using a filtration technique employing a 1% suspension of washed red cells and a $3\,\mu m$ polycarbonate membrane. Haematological and biochemical profiles and clinical data were recorded. In Study A Univariate analysis showed a significant difference ($P = 0.023$) in RCF between the patient (mean RCF = 0.219) and the control group (mean RCF = 0.235). However, when differences in other biochemical parameters, notably albumin, were taken into account using multivariate analysis there was no longer a significant difference in RCF. There was no significant relationship between RCF and stage of disease. In 45 patients on whom RCF measurements were repeated after 3–6 months there was no relationship between change in disease state and RCF. Variability in these repeat samples was much larger in the patient group than in controls. In the SCLC patients treated with etoposide (Study B), post-treatment RCF was significantly higher than pre-treatment values ($P = 0.0007$) and showed an improvement in 12 of 15 patients (median increase 16.3%). In conclusion RCF is not altered in cancer patients or by changing disease state, but may be improved by chemotherapy.

### A DNA cytophotometric study of neoplastic field change in the human urinary bladder

A. O'Brien[1], A. Dorman[2], M. Butler[1] & J.M. Fitzpatrick[1]

[1]*Department of Urological Research, Meath Hospital, Dublin and* [2]*Department of Pathology, St James's Hospital, Dublin, Eire.*

Hofstaedter *et al.* (*Br. J. Urol.*, **56**, 289, 1984) have shown that the Diploid Deviation Quotient (DDQ) is a reliable indicator of future behaviour of bladder tumours, irrespective of grade or stage, in that the survival of patients whose tumours had DDQ's < 1.2 did not differ from that of controls, whilst a DDQ > 1.2 was associated with a poor prognosis ($P < 0.05$). The aim of this study was to apply DNA cytophotometry to the assessment of neoplastic field change in the bladder. Cold cup biopsies of *endoscopically normal* mucosa were taken from 7 predetermined, disparate sites in each of 10 bladders containing a tumour, as well as a biopsy of the tumour itself. Similar biopsies from 10 benign bladders acted as controls. Adjacent cuts from each specimen were submitted for histopathology and cytophotometry using a scanning microdensitometer.

Histological examination revealed that 4 (6%) of field biopsies contained frank tumour, 6 (9%) carcinoma-in-situ (CIS), 13 (19%) varying types of cystitis, and 44 (66%) normal. Two of those with tumour, all of those with CIS and 4 of those with cystitis had a DDQ > 1.2. However, it was surprising to find that 20 (45%) of the *histologically normal* biopsies had a DDQ > 1.2 ($P < 0.01$). Moreover, in 10 of these cases, the associated tumour had a DDQ < 1.2. This study would indicate that this technique may be able to identify histologically normal mucosa already committed to neoplastic change, and that such mucosa may have even greater eventual prognostic implications than the associated overt tumour.

### *In vitro* and *in vivo* ectopic production of immunoreactive βhCG by neoplastic and normal bladder epithelium

R.K. Iles[1], R.T.D. Oliver[1], M. Kitau[2] & T. Chard[2]

[1]*Medical Oncology Unit, Department of Urology, The London Hospital Medical College, London E1 and* [2]*Department of Reproductive Physiology, St Bartholomew's Hospital Medical College, London EC1, UK.*

Beta chain human chorionic gonadotropin (βhCG) was demonstrated in the culture medium of 6 out of 8 neoplastic and 4 out of 5 'normal' human bladder epithelial cell lines by RIA Levels ranged between 34 and $2{,}376\,iu\,l^{-1}$, while controls ranged between 15 (detection limit) and $24\,iu\,l^{-1}$. In contrast, none of the 8 testicular germ cell tumour cell lines secreted detectable levels of βhCG.

Using a cDNA probe, it was shown that the βhCG gene cluster was not amplified in any of these cell lines.

The production of this protein was suppressed significantly by rIFN-γ but not IFN-α. The IFN-γ suppression was coincidental with induction of MHC class II antigens by these cells.

Urine samples from clinically normal individuals and those with active bladder cancer both demonstrated measurable levels of βhCG, although the highest levels were observed within the cancer group.

The experimental results indicate that immunoreactive βhCG can be produced by the urothelium, and this may prove a useful marker of tumour development and progression.

### Correlations between the clinical, haematological and immunological features of B-cell leukaemias

N.S.A. Stuart[1], P.R. Richardson[2], I.C.M. MacLennan[2] & N.R. Ling[2]

[1]*CRC Clinical Trials Unit and* [2]*Department of Immunology, Universtity of Birmingham, Birmingham, UK.*

The surface antigen expression by neoplastic B-cells from 109 patients with B-cell leukaemia (98 chronic lymphocytic leukaemia (CLL), 11 hairy cell leukaemia (HCL)) was investigated using a panel of B-cell antibodies. Correlations were sought between the surface phenotypes and the clinical, haematological and immunological features of the patients' disease.

*1. Differences in antigenic expression between CLL and HCL*

Several phenotypic differences were found between CLL and HCL. Antigens CD24, CD21, and CD23 showed less expression in HCL than CLL while CD22, HD55, 109-3C2, KiB3 and to a lesser extent 2-7 showed more expression in HCL than CLL.

*2. Correlations between expression of different antigens*

Some antigens were expressed on only a proportion of cases of CLL. Correlations were sought between the expression of these antigens to look for patterns of co-expression. Antigens CD21, NUB-1, CDw40, and CD39 were closely correlated usually being expressed together as were CD21, CD9 and CD23.

*3. Correlations between antigenic expression and clinical features of CLL*

Analysis of the phenotype of CLL cells in relation to clinical, haematological and immunological features of patients' disease did not identify any clear disease groups. However younger patients had significantly less cells expressing mouse red-cell receptors ($P<0.001$) and more cells expressing CD20 ($P=0.03$). Patients whose neoplastic B-cells expressed Ki-B3 had lymphadenopathy significantly more often ($P=0.002$) than those whose neoplastic cells did not and also had more advanced stage of disease ($P=0.01$). Serum IgG and IgM were higher in patients with B-cell clones expressing SHCL-2 a difference not attributable to the presence of a paraprotein. Patients whose malignant B-cells expressed both sIgM and sIgD had significantly lower expression of M-RBC ($P=0.006$), CD23 ($P=0.0001$) and NUB-1 ($P=0.03$) than those expressing either sIgM or sIgD alone. B-cell clones expressing kappa light-chain had higher expression of CD20 ($P=0.01$) and CD21 ($P=0.05$). There were no correlations with overall survival or treatment-free interval though follow-up is at present relatively short.

**Treatment of small cell lung cancer (SCLC) with late intensification following a standard induction chemotherapy regime**

P.D.J. Hardman, J.A. Green, R.D. Errington & H.M. Warenius

*Department of Radiation Oncology, Clatterbridge Hospital, Bebington, Wirral, Merseyside L63 4JY, UK.*

Seventy-seven consecutive, previously untreated patients with biopsy proven SCLC received initial treatment with CAV chemotherapy (cyclophosphamide $1\,g\,m^{-2}$, adriamycin $40\,mg\,m^{-2}$, vincristine $1.4\,mg\,m^{-2}$ i.v. q. 21 days) to CR plus 2 cycles. The 43 men and 34 women had a median age of 62 years (range 33–71 years). Twenty-eight patients had extensive disease and 49 patients had limited disease. Results were as follows: 29 CR (39%), 27 PR (36%). 14 SD (19%) 4 PD and 3 NE. Thirty-seven patients went on to receive 2 cycles of IMVP-16 chemotherapy (ifosfamide $5\,g\,m^{-2}$, mesna $7\,g\,m^{-2}$, methotrexate $30\,mg\,m^{-2}$ on day 1, VP16 $100\,mg\,m^{-2}$ i.v. for 3 days i.v. q. 28 days). Five patients were converted from PR to CR by IMVP-16, giving a total of 34 CR (44%) rate. The CAV induction schedule caused only 2 episodes of neutropenic fever. There were 3 deaths on IMVP-16 and 5 further episodes of neutropenic fever, (13.5%). Of this group, 20 have undergone interval bronchoscopy of which 14 (70%) showed no evidence of tumour. Thirteen out of the 34 patients in CR went on to receive planned radiotherapy to the brain (30 Gy) and to the involved lung field (45 Gy) and a further 5 required local radiotherapy on relapse. The median survival figures were: overall 9 months, CR patients 11.5 months, LIM 12 months, EXT 5 months. This sequential treatment regime is feasible and gives results comparable to those in other large series. There is no evidence to date that intensification with IMVP-16 chemotherapy, or local radiotherapy are associated with a prolongation in survival.

**Carboplatin (Cp), VP16 (V) and ifosfamide (I) intensive chemotherapy (CT) for small cell lung carcinoma (SCLC)**

T.J. Perren, M.D. Vincent, M.E. Gore, J.R. Yarnold & I.E. Smith

*Lung Unit, Royal Marsden Hospital, Sutton, Surrey, UK.*

Cp + V has proved very active against SCLC (85% response) but with short duration. A more intensive full dose 3-drug combination of Cp $400\,mg\,m^{-2}$ i.v. day 1, V $100\,mg\,m^{-2}$ i.v. days 1–3, and I $5\,g\,m^{-2}$ day 1 (24 h infusion) with mesna, q. 28 days × 6 courses has therefore been used in 31 fit (ECOG performance status 0–2) previously untreated SCLC pts. Limited disease (LD) patients also received hyperfractionated thoracic radiotherapy concurrently with the first 2 CT courses (15 Gy × 15# × 5 days × 2 courses). Twenty-seven pts (18 males) are evaluable (≧2 courses CT), median age 60 (30–70) yrs. Response is as follows:

| | *Patients* | *CR* | *PR* | *Overall response* |
|---|---|---|---|---|
| LD | 13 | 7 (54%) | 5 | 12 (92%) |
| ED | 14 | 4 (29%) | 10 | 14 (100%) |

Six ED and 2 LD patients (1 CNS alone ) have relapsed; median follow-up is <1 year. Main toxicity was myelosuppression: 15 (56%) patients required dose reduction, 1 (4%) treatment delay, and 2 (7%) had treatment deaths. Main toxicity details are as follows:

| | *WHO Grade* | | |
|---|---|---|---|
| | 0 | 1–2 | 3–4 |
| WBC suppression | 1 | 0 | 26 (96%) |
| Platelet suppression | 3 | 2 (7%) | 22 (81%) |
| Infection | 8 | 10 (37%) | 9 (33%) |
| Nausea/vomiting | 0 | 14 (50%) | 13 (50%) |
| Alopecia | 0 | 1 (4%) | 26 (96%) |

There was no significant neuro-, nephro- or other toxicity. This intensive combination is feasible and highly active but causes severe marrow suppression, and would only be of clinical value if it can achieve good long term control.

**Acute phase reaction in patients with small cell lung cancer undergoing chemotherapy**

R. Milroy, D. Shapiro, A. Shenkin & S.W. Banham

*Departments of Respiratory Medicine and Biochemistry, Royal Infirmary, Glasgow G31 2ES, UK.*

The level of acute phase reactant C-reactive protein (CRP) is raised in inflammatory reactions and tissue destruction and in malignant disease (Raynes & Cooper, *J. Clin. Pathol.*, **36**, 798, 1983). We have measured CRP levels in patients with small cell lung cancer undergoing their first pulse of induction chemotherapy to assess if there is evidence of an acute phase response to chemotherapy and to relate this to subsequent tumour regression. CRP levels were measured on at least 3 consecutive days in 20 patients with small cell lung cancer during their first pulse of chemotherapy. Restaging after 4 cycles showed 7 patients had a complete, 4 a partial and 4 no response to treatment. (Five patients were nonevaluable.) Mean baseline CRP level was elevated ($24.7\,mg\,l^{-1}$) and a dramatic rise in CRP was seen in 14 of 20 patients with levels more than doubling some 24–48 h after induction therapy commenced. Four of the 7 patients who entered complete remission showed this type of acute phase response (CRP peaks $>40\,mg\,l^{-1}$). Two of the remaining 3 patients had low baseline CRP levels with no rise during chemotherapy, possibly reflecting their documented low tumour burden at outset. These data indicate that there is a previously undescribed quantifiable acute phase reaction following chemotherapy the significance of which merits further investigation.

### Neoadjuvant chemotherapy and radiotherapy in non-small cell lung cancer (NSCLC)

S.W. Watkin, R.D. Errington, J.A. Green & H.M. Warenius

*CRC Department of Radiation Oncology, Clatterbridge Hospital, Wirral, Merseyside L63 4JY, UK.*

This pilot study examines the feasibility of the sequential combination of a 4 drug combination regime followed by lung irradiation in NSCLC. Twenty-three patients (16 male, 7 female) with NSCLC have been entered into the study. The mean age was 53.8 years (range 35–71 years) and histology was squamous 12, adenocarcinoma 4, large cell 2, undifferentiated 4, and alveolar cell carcinoma 1. Patients were given adriamycin $40\,mg\,m^{-2}$, vindesine $3\,mg\,m^{-2}$, ifosfamide $5\,gm\,m^{-2}$ with mesna and *cis*-platin $60\,mg\,m^{-2}$, q. 28 days for 3 cycles. Of 17 evaluable patients, there have been 2 CR, 7 PR, 5 SD and 3 PD, giving an overall response rate of 53% to chemotherapy. Seven have died and the median survival is 6 months ($1\frac{1}{2}$ to 18 months), with 2 patients alive at 15 and 18 months. Blood transfusion has been required in 5 and there have been 3 episodes of neutropenic fever and no treatment related deaths. Radiotherapy to a total dose of 4,800 cGy in 16 fractions has been given to 15 patients and was not associated with untoward side effects.

This study has demonstrated that this 4 drug intensive chemotherapeutic regime produces favourable response rates in NSCLC with acceptable toxicity. It remains to be shown whether the combination of chemotherapy and radiotherapy leads to increased survival in this disease.

### Immunohistochemical phenotype related to prognosis in small cell lung cancer (SCLC)

L. Morittu[1], M.N. Sheppard[2], L.G. Bobrow[2] & R.L. Souhami[1]

[1]*Department of Radiotherapy and Oncology and* [2]*Department of Pathology, University College Hospital, London WC1 6AU, UK.*

It has been suggested that those forms of SCLC showing a 'variant' phenotype in culture may be associated with a more malignant clinical course.

We have retrospectively examined initial fibre-optic bronchoscopy specimens of 370 patients treated in a single clinical trial of SCLC. The aim was to determine if there was an immunohistochemically defined phenotype which identified short or long survivors. Twenty-six specimens from short survivors (survival time <4 months) and 36 from long survivors (survival time >12 months) were examined. Of these 12 and 17 were discarded from the short and long groups respectively because of traction artefact or insufficient material. Using the avidin–biotin and the PAP (peroxidase–anti-peroxidase) methods, we have utilised a panel of antibodies which detect neuroendocrine markers (neuron specific enolase (NSE)), and protein gene product (PGP); epithelial antigens ($HMFG_2$ and $AuA_1$); cytokeratins (5.2 and $LP_{34}$) and natural killer cells (Leu 7).

Tumours were scored as either negative or positive, and the percentage of positives in short (S) and long (L) survivors were: NSE 100% (S), 100% (L); PGP 100% (S), 84.2% (L); $HMFG_2$ 92.8% (S), 73.6% (L); $AuA_1$ 85.7% (S), 63.1% (L); 5.2 92.8% (S), 63.1% (L); $LP_{34}$ 7.1% (S), 0% (L), and Leu 7 30.7% (S), 50% (L). The results so far do not suggest that the prognosis of SCLC can be related to immunohistochemical phenotype using markers which recognise epithelial and neuroendocrine antigens. The study is being extended to include more specimens to determine if the small difference in positivity with Leu 7 is genuine.

### Measurements of *in vitro* drug sensitivity to human myeloma and chronic lymphocytic leukaemia

A.G. Hall, A.M. Dickinson, E.A. Jacobs & S.J. Proctor

*Department of Haematology, Royal Victoria Infirmary, Newcastle-upon-Tyne NE1 4LP, UK.*

Assessment of drug sensitivity to treatment is multiple myeloma would be of great clinical benefit. We have been attempting to measure drug sensitivity of myeloma plasma cells to melphalan using 2 independent *in vitro* techniques. One is a recently described cytotoxic assay (Bird *et al.*, *Hematol. Oncol.*, **3**, 1, 1985) using supra-vital staining with fast green and nigrosan and the other an *in vitro* colony assay using 0.9% methylcellulose (Durie *et al.*, *Blood*, **61**, 5, 1983). Separation of plasma cells using Percoll Density gradients (Atherton *et al.*, **55**, 271, 1985) has enabled us to concentrate plasma cells from bone marrow aspirates containing as few as 10% tumour cells. Preliminary results using these *in vitro* methods have measured drug sensitivity of plasma cells down to $0.1\,\mu g\,ml^{-1}$ melphalan.

Further studies involving *in vitro* drug sensitivity of chronic lymphocytic lymphoma (CLL) to chlorambucil and melphalan are in progress.

### The relative value of bone marrow aspirate and trephine in staging non-Hodgkin's lymphoma

R.S. Evely & B.W. Hancock

*Department of Medicine, Royal Hallamshire Hospital, Sheffield, UK.*

A prospective study in 123 patients with non-Hodgkin's lymphoma, compared the relative value of performing both bone marrow aspirate and trephine in routine staging. The original reports for both procedures were classified into one of three categories:

+ = Unequivocal marrow involvement with lymphoma
− = No evidence for marrow involvement with lymphoma
± = Suspicious of marrow involvement with lymphoma

The results are tabulated below:

| *Aspirate* | *Trephine* | *% Cases* |
|---|---|---|
| − | − | 64.0 |
| − | + | 7.3 |
| + | − | 7.3 |
| + | + | 2.4 |
| ± | − | 18.0 |
| ± | + | 4.0 |

Trephine biopsy was valuable in distinguishing reactive changes from lymphoma infiltration. Aspiration failed to detect infiltration in 7% of cases with positive trephine biopsies; however the converse was also true. Six of the 9 patients with positive aspirate/negative trephine died of lymphoma within 18 months of presentation, the majority with obvious evidence of marrow involvement. Aspiration seems to be complementary to trephine biopsy in the staging of patients with non-Hodgkin's lymphoma.

### Appetite and tryptophan in cancer patients

M.L. Slevin[1], S.P. Joel[1], W. Gregory[1], H. Plant[1], L. Stubbs[1], A. Johnston[2], M. Smythe[2], D. Perrett[3] & T. Silverstone[4]

*Departments of [1]Medical Oncology, [2]Clinical Pharmacology, [4]Psychiatric Medicine and [3]Medical Professorial Unit, St Bartholomew's and Homerton Hospitals, London, UK.*

It has been suggested that an increase in brain serotonin results in decreased appetite, a common problem in cancer patients, and that circulating free tryptophan (TRY) correlates well with brain serotonin levels. Brain TRY levels are influenced by 3 factors; other neutral amino acids which compete for blood–brain transport, circulating albumin to which it is bound and free fatty acids (FFA) which also bind to albumin (*Cancer Treat. Rep.* **65**, 15, 1981). This hypothesis has been investigated by measuring all these factors in a group of 218 cancer patients and 100 controls. Food intake and appetite were also assessed.

| Results | Controls | Cancer patients: Normal food intake | Cancer patients: Poor food intake |
|---|---|---|---|
| Albumin | 42.6 | 38.0 | 34.3 ($P<0.001$) |
| FFA | 0.4 | 0.36 | 0.4 ($P=0.007$) |
| Total TRY | 59.5 | 51.7 | 44.9 ($P<0.001$) |
| Bound TRY | 46.2 | 38.3 | 30.6 ($P<0.001$) |
| Free TRY | 13.3 | 13.5 | 14.4 (NS) |
| % Free TRY | 22.3 | 27.0 | 33.7 ($P<0.001$) |

Cancer patients had significantly lower albumin and total bound TRY levels compared to controls, while % free TRY was significantly higher. However, FFA became more importantly, free TRY levels were unchanged. These differences became more pronounced in the patients with poor appetite, though free TRY levels were unaltered. These findings were not influenced by differences in weight loss between the patients.

### The effect of performance status on survival

L.A. Gumbrell, I.R. Judson, S.E. Ashley & A.H. Calvert

*Drug Development Section, Institute for Cancer Research, Sutton, Surrey, UK.*

The purpose of Phase I clinical trial is to evaluate a drug's toxicity and determine a safe dose and schedule for its use in Phase II studies. One of the usual entry criteria is a life expectancy of at least 3 months. Histology, disease stage and tumour bulk are all routinely used to help determine this, but can be unreliable prognostic factors. Is performance status a more reliable indicator? We studied 58 patients (pts) with ovarian cancer who were entered into a Phase I study, age range 30–73 (mean 55). We investigated the effects of age, performance status (PS), tumour volume, response to previous chemotherapy, stage at relapse, pre-treatment alkaline phosphatase, serum albumin, creatinine and FBC on survival. The effect of WHO PS was marked:

| *WHO grade* | *Number* | *Median survival (days)* |
|---|---|---|
| 0 | 11 | 316 |
| 1 | 30 | 146 |
| 2 | 12 | 102 |
| 3/4 | 5 | 50 |

Two other variables had an independent effect on survival, which was adversely influenced by Hb $<11\,g\,dl^{-1}$ or serum albumin $<30\,g\,l^{-1}$. Further analysis is required to define the effect of previous *cis*-platinum treatment on anaemia in these pts. Median survival in relation to albumin was 165d: $>35\,g\,l^{-1}$; 152d: $30–35\,g\,l^{-1}$; 50d: $<30\,g\,l^{-1}$. From these results we conclude that PS is a powerful prognostic indicator. In this group of refractory ovarian cancer pts poor survival was also predicted by a low albumin ($<30\,g\,l^{-1}$). These results strongly suggest that pts whose WHO PS is 3 or 4 should be excluded from Phase I clinical trials.

### Computer-matched bone scanning; increased sensitivity shown by clinical follow-up

D. Parker[1] & G. Hart[2]

*[1]Oncology Unit, Bradford Royal Infirmary, Duckworth Lane, Bradford BD9 6RJ and [2]Department of Nuclear Medicine, Bradford Royal Infirmary, Duckworth Lane, Bradford BD9 6RJ, UK.*

Bone scan data on 72 consecutive scans has been analysed by a series of computer programs to increase the sensitivity of the technique.

An IGE 400A/T gamma camera was interfaced to a Nodecrest data processor and scintiscans were acquired after i.v. injection of 99mTc-MDP. A profile was selected down the whole length of the spine and this was truncated to avoid bladder uptake. The profile was then interpolated to a standard length of 200 points to remove effects due to different patient height; it was then normalised to a standard count level. The profile was examined for statistically significant deviation from a standard curve, obtained from patients referred with benign conditions outside the spine.

Clinical and biochemical data were available on 68 of the 72 patients. Serum alkaline phosphatase was significantly correlated with total abnormal counts and the scaled total number of counts recorded. Forty-five of 68 (66%) of the computer-matched scans (CMS) showed abnormal peaks in the spine, compared with 18 of 68 (26%) of the visually assessed scans. There were a further 13 visually assessed scans which showed lesions outside the spine and which were positive by CMS. Six patients with positive CMS had equivocal visual scans and 8 with positive CMS had negative visual scans.

The clinical state of the patients was reassessed at 14 months after the scan. Of the 6 patients with equivocal visual scans and positive CMS, 5 had died of metastatic disease and 1 was alive but with metastases. In the 8 patients with negative visual scan and positive CMS, 5 have developed metastatic disease and one of these has died. All 5 patients in these 2 groups who had positive CMS and elevated alkaline phosphatase have died of metastatic disease. Computer analysis appears to substantially increase the sensitivity of bone scanning.

**Prospective validation of a simple formula for determining carboplatin dosage**

D.R. Newell, A.H. Calvert, S. O'Reilly, M. Burnell, L.A. Gumbrell, F.E. Boxall, M.E. Gore & E. Wiltshaw

*Drug Development Section and Section of Medicine, Institute for Cancer Research and Royal Marsden Hospital, London and Sutton, UK.*

Carboplatin has become established as a non-toxic platinum complex with activity against a variety of human cancers. Pharmacokinetic studies have shown that carboplatin is eliminated primarily by renal excretion via glomerular filtration (Harland *et al., Cancer Res.*, **44**, 1693, 1984). An equation has been derived for calculating the dose required to achieve a given area under the plasma carboplatin concentration *vs.* time curve (AUC) taking into account pretreatment glomerular filtration rate (GFR) (Calvert *et al., Cancer Treat. Rev.* **12** (supp. A), 51, 1985). Dose (mg) = AUC (mg ml $min^{-1}$) × [1.2 × GFR (ml $min^{-1}$) + 20]. This equation has been used to determine the dose of carboplatin required to treat 26 patients (35 courses) GFR 32–135 ml $min^{-1}$. Free carboplatin plasma levels were determined by atomic absorption spectrophotometry following ultrafiltration (MW < 50,000). Over the AUC range 3–7 mg ml $min^{-1}$ (dose 200–1,000 mg carboplatin), the ratio of the predicted to the observed AUC was constant at 0.89 (95% limits 0.85–0.93). The correlation between plasma free carboplatin clearance and GFR was linear ($r$ = 0.89) with a slope of 0.94 (95% limits 0.76–1.11) in this patient population. Non-renal clearance was 27 ml $min^{-1}$ (95% limits 13–41) and may be correlated with surface area. On the basis of these data we propose, therefore, a modified dose prediction equation for adults: Dose (mg) = AUC (mg $ml^{-1}$ $min^{-1}$) × [GFR (ml $min^{-1}$) + 25]. After 14 courses of carboplatin given to previously treated patients, thrombocytopenia (% pretreatment on days 14–23) correlated linearly ($r$ = 0.85) with AUC, an AUC of 5 mg $ml^{-1}$ $min^{-1}$ producing a drop of ~50%. Using the modified equation the carboplatin AUC required for optimal activity, with manageable toxicity, can now be determined.

**Glycoprotein synthesis in normal and malignant cervical tissue**

M.E.R. O'Brien[1], M.E. Cowan[2], B.E. Souberbielle[3], D. Luesley[4], J.J. Mould[5], A. Buchan[2], G.R.P. Blackledge[1] & G.R.B. Skinner[2]

[1]*CRC Clinical Trials Unit, Queen Elizabeth Hospital,* [2]*Department of Medical Microbiology, Medical School,* [3]*Selly Oak Hospital,* [4]*Department of Obstetrics and Gynaecology, Dudley Road Hospital and* [5]*Department of Radiotherapy, Queen Elizabeth Hospital, Birmingham, UK.*

Glycosylated proteins have important roles in both cancer and normal tissue e.g. cell surface membrane hormone receptors, immunoglobulins and HLA antigens.

To study these glycoproteins in cervical cancer and normal cervical tissue we have used an organ culture system adding radioactive labelled sugars (14C and 3H fucose, mannose, and glucosamine) and protein precursor (35S methionine). The labelled glycoproteins are then separated by SDS-page electrophoresis and autoradiographs analysed.

The incorporation of the sugar precursors appears not to be directly dependent or related to the degree of protein synthesis. To date 14 squamous cell carcinomas and 8 normals have been cultured with results available for 12 carcinomas and 8 normals. There is a large degree of correlation between the normals and the abnormals. Differences in favour of carcinoma appeared in the following molecular weight clusters: 143–147 kd (5/10 ca, 0/8 normals), 76–80 kd (11/12 ca, 1/8 normals), 44–47 kd (10/12 ca, 0/6 normals), 34 kd (7/8 ca, 0/7 normals). Some of these correlate with bands already described in different carcinomas.

Organ culture is a useful technique for studying synthesis *in vitro* in cervical cancer. We have highlighted an area of interest (mol wt 76–80 kd) for antibody formation.

**Renal function following *cis*-platin (CDDP) and carboplatin (JM8) chemotherapy**

M. Teeling, R. O'Regan & D.N. Carney

*Medical Oncology Unit, Mater Hospital, Dublin and Department of Pharmacology, UCD, Dublin, Eire.*

Acute evidence of renal tubular damage (RTD) as shown by a significant rise in urinary BNAG is frequently observed in patients receiving CDDP of JM8. However, the relationship between acute changes in BNAG excretion seen with each administration of CDDP and JM8 and long-term RTD remains uncertain. We have calculated long-term renal function in patients who have completed chemotherapy with CDDP ($n$ = 8) or JM8 ($n$ = 4) and who were at least 3 months (range 3–18 months) from end of therapy. At the time of study, CDDP was administered with prehydration and forced diuresis while JM8 was administered as a bolus infusion over 30 min. Renal function was assessed using plasma creatinine and urea, Cr* EDTA clearance, proteinuria, glycosuria, enzymuria (BNAG) and urinary concentration and acidification. Plasma magnesium (Mg) and urinary Mg excretion were also studied. The mean total dose of CDDP administered was 856 mg (range 540–1,505 mg) and JM8 2,758 mg (range 2,725–2,850 mg). Plasma creatinine and urea levels were within the normal range for all 12 patients studied. Only 1/8 patients who received CDDP showed evidence of RTD (poor concentration and acidification with urinary Mg wasting and hypomagnesaemia). None of the JM8 patients showed RTD. Among 8 patients, followed prospectively from the time of diagnosis, and who had shown a 2–20 fold rise in urinary BNAG excretion with each cycle of chemotherapy, no evidence of permanent RTD as assessed was observed at the time of study. These results suggest (1) long-term RTD in patients receiving either CDDP or JM8 is minimal as currently administered (2) a poor correlation exists between acute elevations of urinary BNAG with therapy and long-term RTD suggesting that the value of BNAG, as a predictor of long-term RTD in patients receiving CDDP or JM8 is of questionable value.

**CA 125 and survival in ovarian cancer**

D. Parker, K. Patel, J. Alred, P. Harnden-Mayor & B. Naylor

*Oncology Unit and Department of Pathology, Bradford Royal Infirmary, Duckworth Lane, Bradford BD9 6RJ, UK.*

CA 125 is an epithelial membrane antigen which is of value in monitoring patients with ovarian cancer. We have been able to show that the initial level of CA 125 is also correlated with survival in ovarian cancer.

CA 125 was assayed in 42 patients with epithelial ovarian cancer using the Centocor 125 kit (CIS UK Ltd). The patients presented after surgical diagnosis and/or debulking. Using an arbitrary level of 250 U $ml^{-1}$, it was found that 13 patients had more than this level on presentation and 29 patients showed lower values. Median follow-up was 10 months. Median survival for patients with CA 125 greater than 250 U $ml^{-1}$ was 310 days, but for values less than

250 U ml$^{-1}$, the projected probability of survival is 0.91. Logrank analysis shows a significant difference between the survival of these 2 groups ($\chi^2 = 7.79$, 1 df, $P = 0.003$).

These results add to the evidence validating CA 125 as a marker for common epithelial cancer of the ovary. It may be possible to use the presentation CA 125 level with clinical and other biochemical measurements to stratify patients for treatment.

**Familial teratoma: Case report and review**

T. Young[1], N. Affarra[2], G. Forrest[1] & M. Soukop[1]

[1]*Department of Medical Oncology, Glasgow Royal Infirmary and* [2]*Department of Medical Genetics, Royal Hospital for Sick Children, Glasgow, UK.*

The familial occurrence of certain cancers including family cancer syndromes are well documented. Reports of familial testicular tumours are relatively rare, there being only 44 case reports occurring in twin and non-twin brothers. However, few have had the same histology. We here report the first description of three non-twin brothers affected by testicular tumours of similar histology. The brothers, PB, TB and AB presented in 1971, 1974 and 1986, respectively. All had been previously healthy with normal testicular development. Review of histology showed all three to have malignant teratoma of intermediate grade. Previous workers have shown some of these tumours to have chromosomal abnormalities. However, due to fixation of tissue at surgery this could not be studied in our patients. However, chromosomal analysis using *ras* gene probe in peripheral leucocytes revealed no chromosomal abnormality and all patients had normal karyotype. The absence of a chromosomal abnormality in peripheral leucocytes may be expected but if present would be a useful marker. We suggest that where possible fresh tumour tissue should be examined in all such familial cases for gene probing to attempt to improve our understanding of tumour development.

**Peritoneal lavage fluid CA 125: A means of increasing sensitivity for this tumour marker in epithelial ovarian cancer (EOC)**

C.W.E. Redman, F.G. Lawton, K.K. Chan & G. Blackledge

*West Midlands CRC Clinical Trials Unit, Queen Elizabeth Hospital, Birmingham, UK.*

Non-nvasive assessment of small volume residual disease in epithelial ovarian cancer remains difficult despite the recent advent of the serological tumour marker CA 125. Serum CA 125 levels correlate well with disease response but there is a false negative rate of 10–20% in bulky disease, and 40–50% in patients in apparent complete remission (Berek *et al.*, *Obstet. Gynecol.*, **67**, 685, 1986). Ovarian cancer remains intraperitoneal throughout most of its natural history, and therefore peritoneal lavage fluid (PLF) CA 125 may be a better indicator of minimal disease.

We are studying CA 125 levels in PLF and correlating these with serum levels. Ten patients with clinically evaluable EOC, 29 women undergoing surgery for gynaecological condition other than EOC (ovarian cyst 4; fibroids 2; endometriosis 2; dysfunctional uterine bleeding 9; cervical squamous cell carcinoma 5; misc. 4) and in 3 patients receiving chronic peritoneal ambulatory dialysis (CAPD) were studied in this way. Peritoneal lavage with 1 l normal saline was performed via a percutaneous cannula in 8 patients (ovarian cancer 5; CAPD 3), and during operation in the remainder. No problems were experienced during this procedure, which was carried out on an outpatient basis for those patients with a percutaneous catheter. CA 125 levels were measured using the Centacor CA 125™ RIA test.

Serum CA 125 levels for the cancer group were all elevated, 77–475 U ml$^{-1}$ (mean 195). PLF levels correlated with these, 84–499 U ml$^{-1}$ (mean 243). Both serum and PLF levels were significantly higher in the cancer group than the controls ($P = 0.01$). These results indicate that PLF can be safely obtained and that the CA 125 levels correlate with those in serum. Further studies are underway in patients with clinically inevaluable disease to determine whether PLF CA 125 levels may be a more sensitive indicator of disease residuum.

**A comparison of serum tumour markers in patients with ovarian carcinoma**

J.N. Gennings[1], A.E. Nelstrop[1], D.S. Northcott[1], G.J.S. Rustin[1], H.J. Lambert[2] & K.D. Bagshawe[1]

(for the N.W. Thames Ovary Group)

[1]*Department of Medical Oncology, Charing Cross Hospital, London W6 and* [2]*Department of Radiotherapy, Hammersmith Hospital, London W12, UK.*

Serum samples have been collected from patients with ovarian carcinoma, and records kept of stage of disease, treatment and clinical status.

Tumour marker CA 125 was measured in serum by ELISA (Abbott Labs kit).

Assays have been developed in the Department of Medical Oncology, Charing Cross Hospital using a 'family' of monoclonal antibodies which may bind to related antigenic epitopes. The following antibodies were investigated: HMFG-1, HMFG-2, M8, NCRC-11 and 115D8.

These assays were used to establish marker levels in serum from a normal population and in the ovarian cancer patients.

In an initial pilot study, serum was assayed from 20 patients with active disease. The percentage of patients with elevated levels of the markers was as follows: CA 125 95%; HMFG-1 5%; HMFG-2 60%; M8 60%; NCRC-11 55%; 115D8 55%.

In 100% (20/20) of patients at least one of the markers was elevated, and excluding CA 125 80% (16/20) expressed an abnormally high level of at least one marker.

Comparison of the different assays in terms of the relative numbers of patients who express these markers, and in the correlation of marker levels with the patients' clinical status has been undertaken.

**Expression of histocompatibility antigens on human ovarian tumour cells**

RCF Leonard & F.G. Hay

*University Department of Clinical Oncology, Western General Hospital, Edinburgh EH4 2XU, UK.*

Our previous experience of phenotyping the tumour cells in human ovarian carcinoma ascites indicated frequent anomalous expression of MHC class II antigen as defined by the pan reactive anti MHC class II antibody CR3.43. We have extended our original observations and have compared the pattern of expression in ascites from 18 individuals using a panel of anti MHC class II reagents. Our current interpretation of the data is that anomalous class II expression is especially strong in the less well differentiated adenocarcinomas but relatively less so in better differentiated serous adenocarcinomas. Loss of MHC class I antigens, defined by the anti $\beta_2$ microglobulin antibody $\beta BM_1$ and

HLA-A-B-C heavy chain anti class I antibody 92.1, is a frequent finding but does not correlate positively or negatively with anomalous expression of class II antigen. Using cell lines derived from such tumours we are examining in detail the extent of this anomalous expression and attempting to determine possible preferential expression of the sub-locus antigens DP, DR and DQ.

### Characterisation and properties of 9 human ovarian adenocarcinoma cell lines

S.S. Lawrie, S.P. Langdon, I.P. Hayward, A. McDonald, M. Hawkes & J.F. Smyth

*Imperial Cancer Research Fund Medical Oncology Unit, Western General Hospital, Edinburgh, UK.*

Several series of cell lines have been derived from patients with ovarian cancer. Nine cell lines have been established; 8 from malignant effusions; 1 from a solid metastasis. Tumour cells were washed (RBCs removed over Ficoll-hypaque), plated at $10^5 ml^{-1}$ in RPMI 1640+FCS with filtered non-autologous human ascitic fluid, refed weekly and subcultured 4 days to 6 months later.

Six lines were derived from poorly differentiated adenocarcinomas (PD): 3 lines (PEO1, PEO4, PEO6) from 1 patient, 2 lines from a second (PEA1, PEA2) and PEO16 from a third. Three lines (PEO14, TO14, PEO23) were derived from a well differentiated serous adenocarcinoma (WD). PEA1, PEO14 and TO14 were obtained prior to treatment; PEO1, PEO4, PEO6, PEA2, and PEO23 after chemotherapy and PEO16 after radiotherapy.

Each set of samples from individual patients produced morphologically distinct monolayer cultures. PEO1, PEO4, PEO6 grew as uniform polygonal cells while PEA1, PEA2 grew in elongated swirls. PEO14, TO14, PEO23 formed foci with small dense cells in the centre and large vacuolated cells at the edges. PEO16 cells were stellate. Doubling times varied between 36 and 120 h. CFEs on plastic were greater for the PD lines (CFE = 1–2%) (except PEO16) than for the WD lines (CFE < 0.04%). Equal or higher coefficients were observed in agar.

Histochemical staining revealed further differences. The WD cell lines contained > 90% cells positive for alkaline phosphatase while the PD lines contained < 10% cells positive (except PEO16).

We are using these models of human ovarian carcinoma to study chemosensitivity, mechanisms of drug resistance and cell differentiation in this disease.

### Oestrogen-receptor-related protein (P29), CEA, EMA, and DD9-E7 in fine needle breast aspirates

E. Heyderman[1], B.M.E. Brown[1], S.E. Larkin[1], S.R. Ebbs[2] & T. Bates[3]

[1]*Department of Histopathology, UMDS, St Thomas's Hospital, SE1 7EH and Departments of Surgery,* [2]*King's College Hospital SE5 9NU and* [3]*William Harvey Hospital, Ashford TN24 0L2, UK.*

The immunocytochemical localisation of oestrogen-receptor-related proteins and of other epithelial markers in fine needle aspirates of breast tumours is of potential value in the selection of treatment and the prediction of outcome. A prospective study of material from women with lesions suspicious of breast cancer has been undertaken. An indirect immunoperoxidase method and monoclonal antibodies directed against the oestrogen receptor related protein (P29) (antibody D5), carcinoembryonic antigen (CEA), epithelial membrane antigen (EMA), cytokeratin (CAM 5.2), and DD9-E7 – a novel epithelial, myeloid and macrophage marker – were used. While the other antigens survive a variety of fixatives, ethanol or methacarn fixation are required to demonstrate P29. Initially 52 sets of smears were made, fixed for 5 min in absolute ethanol, and allowed to dry before being sent by post for immunostaining. This technique was inappropriate in a busy outpatient clinic. The number of slides from a single case were sometimes too few, and the number of tumour cells varied widely. Fixation of the aspirate in methacarn-filled 1.5 ml microfuge tubes, double embedding in 5% agar and paraffin wax, and cutting of at least 10 sequential sections from each pellet was therefore substituted. Ninety-two cases in all were studied. Of the 52 smears 6 were P29 +ve, 4 were CEA +ve, 16 were EMA +ve and 22 were CAM 5.2 +ve (DD9 was not used on smears). The morphology of the H&E sections of the pellets was easy to interpret, as was the immunostaining. Of the 40 pellets, 2 were unsuitable, 15 were P29 +ve, 14 were CEA +ve, 37 were EMA +ve, 31 were CAM 5.2 +ve and 16 were DD9 +ve. The immunostaining results of the pellets showed encouraging prediction of response to endocrine therapy.

### Expression of differentiation antigens by human mammary tumours indicates endocrine responsiveness

A.D. Baildam, A. Howell, D.M. Barnes, L. Turnbull & R.A. Sellwood

*CRC Department of Medical Oncology and Department of Clinical Research, Christie Hospital and Holt Radium Institute, Manchester M20 9BX, UK.*

The monoclonal antibodies HMFG1 and HMFG2, raised against milk fat globule membranes, recognise carbohydrate epitopes on mucin line molecules in normal and malignant mammary epithelial cells. Inasmuch as the epitopes recognised by HMFG1 and HMFG2 are most highly expressed during lactation, they may be regarded as differentiation antigens. In this study we tested the hypothesis that endocrine response of human mammary tumours is related to tumour differentiation as determined by histological grade and HMFG antigen expression. Formalin-fixed paraffin embedded sections of the tumours from 168 patients with advanced, evaluable cancer of the breast were stained by the immunoperoxidase method using both antibodies. The proportion of cells which stained was estimated from counts of 400 or more and was reproducible to within 10%. In 56 tumours the percentage of cells stained was counted on 2 occasions: agreement to within 10% was seen in 45 of 56 (86%). There was a highly significant association between antigen expression, oestrogen (ER) and progesterone (PR) receptors and histological grade (HMFG1 *vs.* ER $P = 0.001$, *vs.* PR $P = 0.001$ grade $P = 0.009$. HMFG2 *vs.* ER $P = 0.001$, *vs.* PR $P = 0.001$ and grade $P = 0.001$). Response (R) to endocrine therapy was related to the number of cells stained (HFMG1 < 10% cells staining 16 of 61 (26%) responded, > 10% 70 of 107 (65%) responded. HMFG2 < 10% cells staining 10 of 47 (21%) responded, > 10% 74 of 119 (62%) responded. Median survival was 24 months greater when > 10% cells stained. A multivariate analysis of 42 possible prognostic factors for survival indicated that PR was the most important factor ($P = 0.00001$); when PR was omitted the percentage of cells stained with HMFG1 was the most important. We conclude that tumours that express these antigens are responsive to endocrine therapy and associated with a good prognosis. Their estimation gives similar information to steroid receptors with the advantage that fixed archival material may be used.

**The pharmacokinetics of medroxyprogesterone acetate 1 g daily following a loading dose of 4 g in 24 hours**

P.A. Canney[1], S. Virdee[2], T.J. Priestman[1], M. Dowsett[2] & T.N. Latief[1]

[1]*Department of Radiotherapy, Queen Elizabeth Hospital, Birmingham and* [2]*Department of Endocrinology, Chelsea Hospital for Women, London, UK.*

The average time to response of patients with advanced carcinoma of the breast treated with high-dose medroxyprogesterone acetate (MPA), 1 g daily in divided doses, is 9 weeks (Canney *et al.*, *Anticancer Res.*, **6(A)**, 391, 1986). Pharmacokinetic data suggest that at this dosage level it can take 4 to 8 weeks to achieve plateau serum levels in the range of $100\,ng\,ml^{-1}$ or higher. To evaluate the feasibility of reaching therapeutic levels sooner, and so possibly effecting a clinical response within a shorter period, we have performed a pharmacokinetic study of MPA, 1 g 6-hourly for 4 doses followed by 1 g daily in divided doses thereafter. Five patients have been studied to date. No acute toxicity was seen. Considerable inter-patient variability in serum levels of MPA occurred, with the peak serum level, after the first dose, ranging from $10\,ng\,ml^{-1}$ to $352\,ng\,ml^{-1}$ between individual patients. The times to achieve steady state serum levels of $100\,ng\,ml^{-1}$, or higher, were 42 h, 120 h and 10 days. In the remaining patient the serum level never exceeded $80\,ng\,ml^{-1}$ but plateaued at this level after 6 days.

**Immunohistochemical staining for OER, epidermal growth factors ER-D5, and P24 oestrogen regulated protein in breast cancer**

C. Wright[1], G. Horne[1], B. Angus[1], R. Sainsbury[2], G.K. Needham[2], S. Nicholson[2], A.L. Harris[3] & C.H.W. Horne[1]

[1]*Department of Pathology,* [2]*Department of Clinical Surgery and* [3]*Cancer Research Unit, University of Newcastle-upon-Tyne, Newcastle-upon-Tyne, UK.*

Proteins regulated by or related to the oestrogen receptor may prove to be more reliable indicators of prognosis and hormone sensitivity than the receptor itself. An inverse relationship between both tumour ER status and low tumour histological grade, and epidermal growth factor receptor content has been recently demonstrated. In a study of 60 breast cancers immunohistochemical staining was carried out by an indirect immunoperoxidase technique on acetone fixed freeze dried frozen sections using monoclonal antibodies against oestrogen receptor (ER) (ERICA – King & Green), epidermal growth factor receptor (EGFR1 – Waterfield), ER-D5 oestrogen receptor related protein (King), and P24 oestrogen regulated protein (Adams & McGuire). In addition tumour histological grade was determined, and radioligand binding assays for ER and EGFR carried out. Immunohistochemical staining grade was determined on a 5-point scale from zero by 2 independent observers, and scores 1 or less regarded as 'negative'. There was good correlation between staining for both ER and R1, and the corresponding biochemical assays thus providing validation of these antibodies as determinants of receptor status. Relating ER staining to EGFR status, very few ER positive cases were EGFR positive, although the relationship falls short of significance $P<0.1$. Seventy-one per cent and 43% of tumours stained for ERD5 and P24 respectively but no relationship was observed between staining for these and other parameters examined. The significance of staining using these antibodies must therefore await determination of clinical outcome for these patients. Tumours of high histological grade tended to be ER negative by immunocytochemistry ($P<0.05$) but this relationship was not confirmed by the biochemical assay.

**Epidermal growth factor receptor quantitative assays: Cut-off points of clinical relevance**

S. Nicholson, J.R.C. Sainsbury, J.R. Farndon, G.K. Needham & A.L. Harris

*Departments of Surgery and Cancer Research, University of Newcastle-upon-Tyne, Newcastle-upon-Tyne, UK.*

Since 1983 epidermal growth factor receptor (EGFr) assays have been performed on over 200 primary and metastatic human breast tumours by a combination of two point assays and Scatchard plots. Multipoint Scatchard analyses have revealed 2 binding sites. The high affinity site, thought to be of clinical relevance, was shown to have an affinity constant of around 1–2 nM (Sainsbury *et al.*, *Lancet*, **i**, 364, 1985). In an attempt to identify tumours with high affinity binding a concentration of 0.6 nM labelled EGF was used in 2 point assays. This is on the linear part of the binding curve. A cut-off point of $10\,fmol\,mg^{-1}$ membrane protein was chosen, as for oestrogen receptor. Follow-up data on over 140 patients has shown a strong inverse relationship to oestrogen receptor status and a positive correlation between EGFr, early recurrence and death. Recent work has shown that saturation of the high affinity site occurs in the majority of cases at a concentration of 1 nM labelled EGF (kd 0.3–0.6 nM). A 2 point saturation assay is now used with 1 nM labelled EGF (Sp. Act. $\sim 80–130\,\mu Ci\,\mu g^{-1}$), $100\,\mu g$ membrane protein and 100 nM unlabelled EGF for calculation of non-specific binding, made up to a volume of $400\,\mu l$ with buffer. At a cut-off of $10\,fmol\,mg^{-1}$ this assay is slightly more sensitive than before.

**EGF receptor status of histological sub types of breast cancer**

J.R.C. Sainsbury, G.K. Needham, S.R. Nicholson, P. Chambers, J.R. Farndon & A.L. Harris

*Departments of Surgery & Clinical Oncology, University of Newcastle-upon-Tyne, Newcastle-upon-Tyne, UK.*

There is an inverse correlation between epidermal growth factor (EGF) receptor status and oestrogen receptor (ER) on human breast cancer specimens and patients with EGF receptor +ve tumours have a poorer prognosis (Sainsbury *et al.*, *J. Clin. Path.*, **38**, 1225, 1985). Some histological subtypes of breast cancer have a better prognosis. An analysis of 191 breast cancer samples (assayed by a radioligand binding assay – Sainsbury *et al.*, *Lancet*, **i**, 364, 1985) has been made and the EGF receptor status correlated with histological findings.

One hundred and seventy-two invasive ductal tumours were assayed of which 62 (36%) were EGF receptor positive. The previously reported negative association with ER status was again found (8 tumours double +ve, 35 tumours double −ve, 57 ER +ve and EGFr −ve and 54 tumours EGFr +ve and ER −ve: $\chi^2=46.9$, $P<0.000001$).

There were 10 lobular carcinomas (5%) of which 3 were EGFr +ve. There was no clear cut relationship between EGF and ER status for this small group of tumours. The majority of the remaining 9 tumours were EGFr −ve (EGFr shown first). Clear cell carcinoma −/+, 2 mucinous carcinomas −/+ and 1 −/−, 1 carcinoid −/+, 1 fibrosarcoma −/−, 1 tubular −/+ and 2 cystosarcoma phylloides of which 1 was −/− and 1 +/−. Thus only one of the rarer tumours, with a better prognosis, was EGF receptor +ve and 5 were ER +ve. These results tend to support the hypothesis that EGF positivity is related to poor prognosis.

**Increased alkylating activity in patients with bronchogenic carcinoma treated with a fractionated dose of ifosfamide**

T. Cerny[1], J. Margison[2], M. Lind[1], P.M. Wilkinson[2] & N. Thatcher[1]

[1]*Department of Medical Oncology and* [2]*Clinical Pharmacology, Christie Hospital and Holt Radium Institute, Manchester M20 9BX, UK.*

Ifosfamide (Ifos) is a prodrug which requires hepatic activation. In 10 patients with bronchogenic carcinomas alkylating activity in serial serum samples taken after a fractionated daily oral or i.v. dose was assessed using a modified nitrobenzylpyridine spectrophotometric method. Serum Ifos levels were measured by an HPLC method (Margison *et al., Biomed. Chrom.*, **1**, 3, 1986). We have previously shown that the bioavailability of oral Ifos is 100%.

The serum alkylating activity (mean area under the curve) was as follows:

| *Patients (n)* | *Daily dose Ifos* | *Area under the curve* $\mu g\,l^{-1}\,h^{-1}$ *Day 1* | *Day 2* | *Day 3* | *Max increase* |
|---|---|---|---|---|---|
| 4 | 2 g i.v. | 105 | 140 | 99 | 33% day 2 |
| 6 | 2 g oral | 131.1 | 264 | 239 | 101% day 2 |

These findings suggest that a fractionated dose of oral Ifos may result in maximal serum alkylating activity, probably due to induction of metabolism and this should be evaluated further. Marked inter-individual variability, however, has been found.

**Non-invasive pharmacokinetics of misonidazole and chlorambucil analogues by $^{19}$F nuclear magnetic resonance spectroscopy**

P. Workman[1], R.J. Maxwell[2] & J.R. Griffiths[2]

[1]*MRC Clinical Oncology and Radiotherapeutics Unit, Cambridge and* [2]*CRC Biomedical Magnetic Resonance Research Group, Department of Biochemistry, St George's Hospital Medical School, London, UK.*

Nuclear magnetic resonance (NMR) offers exciting potential for the non-invasive investigation of antitumour agent pharmacokinetics in animals and man. The most favourable atomic nucleus is $^{19}$F which gives an intense signal with low endogenous background. Here we report the feasibility of studying the biodistribution in mice of (1) $\beta,\beta$-difluorochlorambucil, $\beta$-$F_2$CHL, a fluorinated analogue of the alkylating agent chlorambucil, and (2) Ro 07-0741, 1-(2-nitroimidazol-1-yl)-3-fluoropropan-2-ol, a fluorinated analogue of the hypoxic cell sensitiser misonidazole. Drugs were administered i.p. to C3H/He male mice bearing the KHT fibrosarcoma im in the leg, $\beta$-$F_2$CHL at 45 mg kg$^{-1}$ and Ro 07-0741 at 473 mg kg$^{-1}$. Mice were anaesthetised with urethane or sodium pentobarbitone. A 15 mm diameter radiofrequency surface coil was placed externally over the liver, tumour or skull. $^{19}$F spectra were recorded with a 1.9 Tesla Oxford Research Systems TMR-32 spectrometer using a frequency of 75.5 MHz, a 1 sec pulse repetition time, and a spectral width of 4 kHz. Spectra were obtained by averaging 480–960 pulses (8–16 min). Relative quantitation was by peak area. $\beta$-$F_2$CHL was identified reproducibly in liver for up to 3 h post-injection. It was not seen in brain or tumour, but amounts $<\frac{1}{3}$ of the liver level would not have been detectable. Ro 07-0741 was readily detected in all 3 tissues. Amounts at early times were similar and declined with a $t_{\frac{1}{2}}$ of $\sim$3 h. Interestingly, there was a clear trend towards preferential retention in liver and tumour compared to brain, possibly reflecting the degree of nitroreductive bioactivation.

**Pharmacokinetics of melphalan in isolated limb perfusion (ILP) for melanoma of the leg**

R.N. Scott[1], R. Blackie[2], D.J. Kerr[2], J. Hughes[1], R. Mackie[3], S.B. Kaye[2] & A.J. McKay[1]

*Departments of* [1]*Surgery and* [2]*Medical Oncology, Gartnavel General Hospital and* [3]*Department of Dermatology, Western Infirmary, Glasgow, UK.*

The rationale for use of ILP in treatment of melanoma of the leg depends on generation of high drug levels within the Isolated limb and low systemic levels with enhancement of the therapeutic ratio. The pharmacokinetics of melphalan (dose = 1.5 mg kg$^{-1}$) have been studied in 10 patients with lower limb melanoma who had external iliac ILP performed for 1 h. The perfusate was sampled from arterial and venous ports on the ILP pump and systemic blood specimens taken from an indwelling radial catheter. Melphalan levels were measured by a sensitive and specific HPLC assay. Peak drug levels ($44 \pm 12\,\mu$g ml$^{-1}$) in the perfusate were considerably higher than those achieved with maximal systemic therapy ($\sim$15–20 $\mu$g ml$^{-1}$), and declined bi-exponentially. Partial AUC analysis of patient and *in vitro* melphalan decay curves indicated that there was no further drug uptake by the limb after 30 min of the 1 h perfusion and that $\sim$30% of the administered melphalan dose distributed into the tissues of the leg. Melphalan was virtually undectable in the systemic circulation during the perfusion, but rose to a peak of $0.8 \pm 0.6\,\mu$g ml$^{-1}$ 30 min after cessation of ILP. Systemic clearance of melphalan was $0.18 \pm 0.04$ l min$^{-2}$ m$^{-2}$, which is lower than the expected value following systemic administration. Our kinetic results show that high concentrations of melphalan can be achieved within the vascular compartment of the perfused limb and that drug uptake by the limb seems to be saturated by mid perfusion.

**Use of low dose methotrexate bolus to determine dosage in high dose methotrexate therapy**

M. Lough[1], G.J. Forrest[2], N.L. Gilchrist[2], I.D. Watson[1], M. Soukop[2] & M.J. Stewart[1]

*Departments of* [1]*Pathological Biochemistry and* [2]*Medical Oncology, Royal Infirmary, Glasgow G4 0SF, UK.*

The aim of this study was to individualise dosage on a pharmacokinetic basis, utilising a low dose i.v. bolus of methotrexate (MTX) (10 mg) prior to high dose therapy (1.5 g m$^{-2}$ over 1 h).

Six patients with high grade non-Hodgkin's lymphoma were studied. Plasma MTX levels were followed for 24 h after low dose and high dose therapies. MTX levels were measured by fluorescence polarisation immunoassay. Comparison of high and low dose pharmacokinetics showed that calculation of high dose MTX from low dose data was no more effective than dosage based on body surface area (Table).

Comparison of predicted plasma MTX concentrations ($\mu mol\,l^{-1}$) with the actual concentrations after high dose infusion

| *Patient* | *EC* | *SJ* | *FW* | *HH* | *MB* | *EF* |
|---|---|---|---|---|---|---|
| Predicted concentration | 38.7 | 63.2 | 63.5 | 51.6 | 24.7 | 85.2 |
| Measured concentration | 118 | 100 | 100 | 45 | 100 | 160 |

### The disposition of vincristine, hepatic function and clinical toxicity in children receiving infusion therapy

B.J. McDermott[1], C.R. Pinkerton[2] & H.W. van den Berg[1]

[1]*Department of Therapeutics and Pharmacology, The Queen's University of Belfast, UK and* [2]*Centre Leon Berard, Lyon, France.*

A Phase II study in children with resistant tumours showed that the administration of a large dose of vincristine (VCR) by long-term i.v. infusion had greater efficacy than a lesser dose given as a bolus, but was associated with a larger incidence of neurological toxicity. To assess the correlation of these side-effects with pharmacokinetic parameters, specimens of plasma were obtained from 9 patients who received a loading of $0.5\,mg\,m^{-2}$ followed by a continuous infusion of $0.5\,mg\,m^{-2}$ daily for 5 days. Concentrations of VCR were determined using a radioimmunoassay of which the lower limit of sensitivity was $0.25\,ng\,ml^{-1}$. Biochemical tests of liver function were performed before therapy, 1–4 times during treatment and up to 25 days after the end of infusion.

The initial concentrations of VCR ranged from 1–11 $ng\,ml^{-1}$ and of 5 cases with values $>6\,ng\,ml^{-1}$, 3 were associated with abnormal neurological sensations during treatment. The mean steady-state concentration was 1.68 (range, 1.29–2.15) $ng\,ml^{-1}$ and delayed neuropathic symptoms occurred in 1 of the 2 patients who had values $>2\,ng\,ml^{-1}$. The total body clearance of VCR was not related to levels of liver enzymes. The drug, however, appeared to cause hepatocellular damage, indicated particularly by the increase of $\gamma$-glutamyltransferase to abnormal levels in the majority of cases and usually after completion of treatment.

### Tyrosinase activity and the cytotoxic effects of 4-hydroxyanisole in metastatic variants of the B16 melanoma

I. Oliver[1], A.J. Thody[1] & and G.V. Sherbet[2]

[1]*Department of Dermatology and* [2]*Cancer Research Unit, University of Newcastle-upon-Tyne, Newcastle-upon-Tyne, UK.*

It has been suggested that the melanocytotoxic effects and anti-tumour activity of 4-hydroxyanisole (4-OHA) are due to its conversion by tyrosinase to toxic intermediates. In the present study we have compared the cytotoxicity of 4-OHA with levels of tyrosinase in 3 variants of the B16 melanoma.

B16F1, B16F10 and B16BL6 melanoma cells were cultured in Eagles MEM with 10% fetal calf serum, antibiotics and non-essential amino acids for up to 4 days. Under these conditions, all cell lines expressed tyrosinase activity, although the F1 cells displayed twice as much activity as the other 2 lines. This difference was also seen in the amount of tyrosinase in the bound form. Lower concentrations ($10^{-6}$ and $10^{-5}$ M) of 4-OHA had little cytotoxic activity in the 3 cell lines. Cytotoxicity was evident at $10^{-4}$ M 4-OHA and most marked in the BL6. At $10^{-3}$ M, cytotoxicity was similar in all 3 cell lines. $\alpha$-MSH ($10^{-7}$ M) increased tyrosinase activity in all 3 cell lines but had no effect upon the cytotoxicity of 4-OHA.

These findings confirm that 4-OHA has cytotoxic properties in melanoma cells. However, they do not support the view that the cytotoxicity of 4-OHA is related to the tyrosinase activity of the cell.

### Phase I clinical pharmacokinetics of *m*-azidopyrimethamine (MZP)

S.M. Crawford[1], R. Hoffman[1], J.A. Slack[2], G.R.P. Blackledge[3], E.S. Newlands[1], N. Stuart[3], S.K. Wong[2], S.K. Pashley[2], C.J. Brindley[1] & M.F.G. Stevens[2]

[1]*Department of Medical Oncology, Charing Cross Hospital Medical School, London,* [2]*CRC Experimental Chemotherapy Group, Pharmaceutical Sciences Institute, Aston University, Birmingham and* [3]*CRC Clinical Trials Unit, Department of Medicine, Birmingham University, Birmingham, UK.*

Meta-Azidopyrimethamine ethanesulphonate (MZPES) is a new lipophilic DHFR inhibitor designed to both overcome transport-mediated resistance and possess a shorter biological half than metoprin or etoprin. MZPES has been shown to be active against P388(+), L1210(++), B16(++), TLX5(+) and M5076(++) but inactive against LL, $CD8F_1$ and Colon38. MZPES has a Ki of 2.4 nM against L1210 DHFR and 1.6 nM against rat liver DHFR.

MZPES was formulated for clinical use as a 10% solution in water and diluted into 500 ml of 5% dextrose prior to i.v. infusion over 1 h. The starting dose for Phase I evaluation was $5.4\,mg\,m^{-2}$ and has been escalated through 11, 18, 27, 38, 50, 67, 83, 105, 125, 150, 180, 210, 250, 300, 360 and $400\,mg\,m^{-2}$. Pharmacokinetic studies have been performed at all doses, except $5.4\,mg\,m^{-2}$ on a total of 34 patients with the drug apparently obeying 2-compartment kinetics with no evidence for dose dependency in the kinetics. The mean distribution and elimination half lives were 0.26 and 33.52 h respectively with an apparent volume of distribution of 1191 and a central compartment volume of 261.

### Disposition and anti-neoplastic efficacy of niosome-encapsulated adriamycin in nude mice bearing lung xenografts

J.G. Morrison[1], A. Rogerson[2], A.T. Florence[2], S.B. Kaye[1] & D.J. Kerr[1]

[1]*Department of Medical Oncology, University of Glasgow and* [2]*Department of Pharmacy, University of Strathclyde, Glasgow, UK.*

We have shown previously that the cellular uptake and cytotoxicity of adriamycin in human lung tumour cells in monolayer and spheroids is enhanced by concurrent incubation with surfactant. Adriamycin has been encapsulated in multilamellar, non-ionic, surfactant vesicles (niosomes), $\sim 1\,\mu m$ in diameter. Free adriamycin solution ($10\,mg\,kg^{-1}$) or an equimolar amount of niosome entrapped adriamycin was administered via a tail vein in nude mice bearing a squamous lung tumour xenograft (WIL). At intervals thereafter groups of 4 mice were sacrificed by exsanguination and the tumour and organs removed for subsequent drug analysis by HPLC. Tumour growth delay was assessed by thrice weekly bidimensional measurement of tumour size after intravenous treatment of 3 groups of 8 mice with free adriamycin ($7.5\,mg\,kg^{-1}$), niosomal adriamycin ($7.5\,mg\,kg^{-1}$) and normal saline as control. Respective mean peak adriamycin levels following administration of free niosomal adriamycin levels following administration of free or niosomal adriamycin were statistically similar in kidney (24.8 $\mu g g^{-1}$ *vs.* 11.3 $\mu g g^{-1}$), liver (27.9 $\mu g g^{-1}$ *vs.* 18.2 $\mu g g^{-1}$) and tumour (4.5 $\mu g g^{-1}$ *vs.* 7.5 $\mu g g^{-1}$), whereas peak plasma (0.12 $\mu g ml^{-1}$ *vs.* 0.075 $\mu g ml^{-1}$) and cardiac (11.5 $\mu g g^{-1}$ *vs.* 5 $\mu g g^{-1}$) concentrations were significantly lower in the niosome treated group. Tumour volume doubling time was significantly longer ($P<0.05$) for adriamycin (15 days) and niosomal adriamycin (11 days) than for control (5.8 days), although there was no difference between the treatment groups. Since antitumour efficacy of adriamycin encapsulated in adriamycin in niosomes is maintained, it is conceivable that this mode of administration will lead to an improved therapeutic index because of the potential reduction in toxicity resulting from lower plasma and cardiac drug levels.

### Intravesical chemotherapy: Comparison of *in vitro* cytotoxicities between monolayers and spheroids

R. Knuchel[1], F. Hofstaedter[1], W.E.A. Jenkins[2], J. Turton[3] & J.R.W. Masters[2]

[1]*Department of Pathology, 5100 Aachen, FRG,* [2]*Institute of Urology, London WC2H 9AE and* [3]*School of Pharmacy, London WC1N 1AX, UK.*

Drugs differ in their ability to penetrate tumours and this factor will contribute to their relative value, for example, in the treatment of superficial bladder cancer with intravesical administrations. Three-dimensional multicellular tumour spheroids provide an *in vitro* model for assessing drug penetration. We compared the *in vitro* cytotoxicities following a 1 h exposure to the 4 drugs most frequently used for intravesical chemotherapy (adriamycin, epodyl, mitomycin, thiotepa) and epirubicin using spheroids and 2-dimensional monolayer cultures. We used the continuous cell line MGH-U1, derived from a transitional cell carcinoma of the human bladder, treating the monolayers and spheroids (0.65–0.75 mm diam.) in an identical manner. The concentrations of drugs which reduced clonogenic cell survival by 50% are shown in the table.

| *Drug* | *IC50 monolayer* ($\mu g ml^{-1}$) | *IC50 spheroid* ($\mu g ml^{-1}$) | *Ratio* |
|---|---|---|---|
| Adriamycin | 0.20 | 2.90 | 14.50 |
| Epirubicin | 0.10 | 1.90 | 19.00 |
| Epodyl | 37.00 | 67.50 | 1.80 |
| Mitomycin-c | 0.52 | 0.96 | 1.80 |
| Thiotepa | 29.00 | 13.00 | 0.45 |

Thiotepa was unique in being more cytotoxic in 3 than in 2-dimensional culture, and this finding is consistent with clinical observations that this is the only drug penetrating the bladder and consistently producing systemic toxicity. Drug penetration is likely to have a major influence on tumour cell kill, but other factors such as pH, oxygenation and cell kinetics are important.

### Interferon/drug combinations as anticancer therapy: What is the optimum dosage schedule?

R.J. Ferguson, L.E. Anderson & J.F. Smyth

*Imperial Cancer Research Fund Medical Oncology Unit, Western General Hospital, Edinburgh EH4 2XU, UK.*

Human alpha interferon (IFN) has been shown to potentiate the activity of sub-optimal doses of certain cytotoxic agents in human cancers grown in mice (Balkwill *et al., Cancer Res.,* **44,** 904, 1984; Carmichael *et al., Cancer Res.,* **46,** 4916, 1986). Pilot clinical studies investigating the potential of these combinations are currently being performed but little is known about the optimum method of administering the agents in combination. We studied the effect of different dose schedules of *cis*-platinum (CDDP) and αIFN in combination on a human squamous lung cancer xenograft in immunodeprived mice. Groups of 8–10 tumours were allocated to either treatment or control. IFN $2\times10^4$ U day$^{-1}$ was given s.c. for 35 days in combination with the MTD of CDDP (7 mg kg$^{-1}$ i.p.) given as (a) 1.4 mg kg$^{-1}\times5$ weekly, (b) 3.5 mg kg$^{-1}\times2$, 10 days apart, (c) a single 7 mg kg$^{-1}$ injection. The median doubling time for each group was calculated and drug activity expressed in terms of the specific growth delay (SGD). IFN alone had no effect but potentiated both CDDP 1.4 mg kg$^{-1}\times5$ (SGD for CDDP alone = 0.88, for combination = 2.00) and 7 mg kg$^{-1}\times1$ (SGD = 2.8, CDDP + IFN = 3.1). The greatest effect was seen with 3.5 mg kg$^{-1}\times2$ where no tumours in the combination group doubled (CDDP alone SGD = 4.25). CDDP (1.4 mg kg$^{-1}\times5$) was then assessed with 3 doses of IFN ($5\times10^4$, $1\times10^5$, $2\times10^5$ U day$^{-1}$). Maximum activity (SGD = 7.0) was seen with the middle dose ($5\times10^4$, SGD = 4.3; $2\times10^5$, SGD = 5.0; CDDP alone, SGD = 0.75). The optimum dosage schedule for IFN/CDDP combinations in this model appears to involve chronic dosing at medium levels rather than high or low doses of either agent.

### Heat sensitivity of tumour tissue

K. Bowler[1], R. Manning[1] & C.J. Barker[2]

[1]*Department of Zoology, University of Durham, South Road, Durham, DH1 3LE and* [2]*Department of Biochemistry, University of Birmingham, PO Box 363, Birmingham B15 2TT, UK.*

The effect of hyperthermia on MC7 sarcoma and D23 hepatoma, transplanted into rat foot, was studied *in vivo* and *in vitro*. Heating *in vivo* delayed growth and in some cases eliminated the tumour. Combined treatment of heat and tetracine (a membrane fluidiser) caused a small potentiation in survival of rats bearing MC7 and a large potentiation of rats bearing the D23 tumour. Hyperthermia also affected a number of membrane-associated functions: (a) Tumour and liver slices were preincubated at a temperature between 37° and 53°C for 1 h, then $O_2$ uptake was monitored at 37°C. Tumour respiration was inhibited after 44–46°C and so was more thermosensitive than liver respiration. Inclusion of tetracaine during the preincubation period sensitised tumour respiration to heat. (b) Incubation of tumour tissue at 44°C caused a marked rise in $K^+$ leakage from cells compared with incubation at 37°C. The inclusion of tetracaine increased $K^+$ loss by 3 to 4-fold in tumour and 1.3-fold in liver. (c) $Mg^{2+}$-ATPase (plasma membrane bound enzyme) showed greater thermal sensitivity in tumour cells compared with normal cells. Inactivation of the tumour enzyme occurred in the range 42–50°C, whereas the liver enzyme was inactivated only above 50°C. The 'fluidity' of plasma membranes derived from the tumours and from normal liver was measured by fluorescence polarisation using 1,6-diphenyl 1,3,5-hexatriene. Tumour plasma membranes were more 'fluid' (decreased order) than liver membranes at all temperatures tested. Tetracaine decreased membrane order in both tumours and in liver. Collectively these data support the hypothesis that the plasma membrane is a primary target in heat injury and that the tumour cell membrane may be particularly susceptible to hyperthermia as a result of the lower order of its lipid moiety.

The principal sponsors of the meeting were: Boehringer Ingelheim Ltd; Bristol-Myers Pharmaceuticals; Ciba-Geigy Pharmaceuticals; Farmitalia Carlo Erba Ltd; ICI Pharmaceuticals PLC; and Lederle Laboratories.

Additional sponsorship was received from: Anachem Ltd; Arnold R. Horwell Ltd; Becton Dickinson Ltd; Beecham Pharmaceuticals Ltd; Bibby Science Products Ltd; Bio-Rad Ltd; Boehringer Corporation Ltd; Cambridge Research Chemicals; Du Pont Corporation (UK); Elkay; Envair UK Ltd; Flow Laboratories Ltd; Gibco BRL Ltd; Imperial Laboratories; Joyce Loebl; Kirby Warrick; LKB Instruments Ltd; Macarthy's Surgical Ltd; MDH Ltd; Meditec; Millipore UK Ltd; MSE Ltd; New Brunswick Scientific Ltd; Northumbria Biologicals Ltd; Nycomed (UK) Ltd; Perkin Elmer Ltd; Roche Products Ltd; Sera Lab Ltd; S.H. Scientific Ltd; Technogen Systems Ltd; Ultra-Violet Products Ltd; and Zeiss.